# Standardized synchronization and validation pipeline for physiological biomarkers across multiple devices

**DOI:** 10.3758/s13428-026-03097-8

**Published:** 2026-07-16

**Authors:** Selin Acan, Clàudia Valenzuela‐Pascual, Filippo Corponi, Bryan M. Li, Diego Hidalgo‐Mazzei, Dick Thijssen, Gideon Vos, Stefan Bogaerts, Erno Hermans, Mitra Baratchi, Martin Dresler, Peter De Looff

**Affiliations:** 1 Donders Institute for Brain, Cognition, and Behaviour, Radboud University, Kapittelweg 29, 6525 EN Nijmegen, The Netherlands; 2 Behavioural Science Institute, Radboud University, 6525 GD Nijmegen, The Netherlands; 3 Science and Treatment Innovation, Fivoor, 3014 AE Rotterdam, The Netherlands; 4 National Expert Centre Intellectual Disabilities and Severe Behavioral Problems, De Borg, 3723 MB Bilthoven, The Netherlands; 5 Department of Developmental Psychology, Tilburg University, 5037 AB Tilburg, The Netherlands; 6 Department of Psychiatry and Psychology, Hospital Clínic de Barcelona, Barcelona, Catalonia Spain; 7 Bipolar and Depressive Disorders Unit, IDIBAPS, Barcelona, Catalonia Spain; 8 Biomedical Research Networking Centre, Consortium on Mental Health (CIBERSAM), Instituto de Salud Carlos III, Madrid, Spain; 9 Department of Medicine, Institute of Neurosciences (UBNeuro), University of Barcelona, Barcelona, Catalonia Spain; 10 School of Informatics, University of Edinburgh, Dugald Stewart Building, 10 Crichton St, Edinburgh, EH8 9AB UK; 11 James Cook University, 18 Joan St, Mornington, QLD 4825 Australia; 12 Leiden University, Rapenburg 70, 2311 EZ Leiden, The Netherlands; 13 Department of Medical Biosciences, Radboud University Medical Center (Radboudumc), Geert Grooteplein 28, 6525 GA Nijmegen, The Netherlands; 14 Donders Center for Cognitive Neuroimaging, Radboud University, Kapittelweg 29, 6525 EN Nijmegen, the Netherlands

**Keywords:** Synchronization, Statistical analysis, Empatica E4, EmbracePlus, Wearables, Alignment, Cross-platform interoperability

## Abstract

Wearable devices enable continuous monitoring of physiological signals in real‐world settings, yet a standardized approach for synchronizing signals across devices remains lacking. We present a generalizable, user‐friendly pipeline that enables synchronization of any two devices capturing the same physiological signals, without requiring coding expertise. The pipeline performs resampling, dynamic time warping alignment, amplitude correction via wavelet transforms, and signal standardization, followed by agreement analyses at both waveform and feature levels. To demonstrate its validity, we applied the pipeline to a case study comparing two research‐grade devices, the Empatica E4 and the EmbracePlus, using up to 48 h of concurrent recordings from 31 participants. We compared signal‐level agreement across waveform similarity, amplitude distribution, spectral content, and extracted features between the two research‐grade devices to determine their interchangeability for longitudinal and multi‐site studies. Specifically, we aimed to determine how well these devices agree at the signal level and to identify which physiological signals are most robust to device‐specific variability. Four signals were examined (blood volume pulse, electrodermal activity, accelerometry, and temperature) using NeuroKit2 and FLIRT. We computed Pearson and concordance correlation coefficients, Bland–Altman bias and limits of agreement, root mean squared error (RMSE), KL divergence, spectral coherence, mutual information, and feature‐level correlations using NeuroKit2 and FLIRT. Results showed near‐perfect agreement for BVP (concordance correlation coefficient (CCC) ≈ 1.0; coherence ≤ 0.98) and phasic EDA features (CCC 0.85–0.99), whereas tonic EDA, temperature, and accelerometry exhibited systematic amplitude biases (EmbracePlus lower) and axis‐dependent variability (*Z*‐axis CCC = 0.85; *Y*‐axis = 0.19). Relative signal dynamics were preserved across devices despite differences in absolute levels. These findings support integration of BVP, EDA, and TEMP data across E4 and EmbracePlus with proper preprocessing, while highlighting calibration needs for movement signals.

**Supplementary Information:**

The online version contains supplementary material available at 10.3758/s13428-026-03097-8.

## Introduction

Wearable technologies are increasingly adopted for continuous, real-world monitoring of physiological signals across a range of domains, including health, stress, sleep, and affective computing (Chan et al., 2012; Marakhimov & Joo, 2017; Nasiri et al., 2020; Sultan, 2015; Zhang et al., 2017). Their growing adoption in both research and clinical practice has created new opportunities for longitudinal monitoring, particularly in psychiatric settings where objective, multimodal biomarkers can support diagnosis, treatment evaluation, and relapse detection. However, this potential is limited by a critical gap: the absence of standardized, accessible pipelines for synchronizing and validating physiological signals across devices. Without such protocols, clinicians and researchers, especially those without programming expertise, face substantial barriers when attempting to integrate data across platforms.

Importantly, the motivation for integrating data across platforms extends beyond technical convenience. While existing toolkits such as NeuroKit2 support multimodal analyses within a single recording system, they do not address the increasingly common scenario in which equivalent physiological signals must be compared or combined across different wearable platforms. This situation arises frequently in longitudinal and multi-site studies, where devices may be upgraded, discontinued, or replaced over time, as well as in collaborative or resource-limited settings where access to identical hardware cannot be guaranteed. In such contexts, the inability to synchronize and validate signals across platforms can limit data reuse, hinder replication, and disproportionately disadvantage smaller or less well-resourced research groups. By enabling systematic cross-platform synchronization and validation, the proposed framework supports continuity across device generations, facilitates integration of legacy and newly acquired datasets, and promotes more equitable and accessible physiological research independent of specific vendor ecosystems.

Several well-established open-source toolkits, such as NeuroKit2 (Makowski et al., 2021) and FLIRT (Föll et al., 2021), provide comprehensive preprocessing and feature extraction pipelines for individual physiological signals. While such tools were used where appropriate in this study, they are primarily designed for single-device analysis and do not address cross-device synchronization or validation when multiple wearable platforms are recorded concurrently. Prior work has highlighted the importance of temporal alignment in multimodal recordings (e.g., van Lier et al., 2020), yet a unified framework integrating synchronization with signal- and feature-level agreement across devices remains lacking. The present study addresses this gap by proposing a standardized approach for cross-device synchronization and validation across multiple physiological modalities.

While prior validation studies have examined the accuracy of wearable sensors compared to clinical gold standards (e.g., ECG for heart rate, skin conductance electrodes for EDA), there remains a gap in understanding how signals from different wearables compare with each other, as denoted by earlier studies (Ronca et al., 2023; Reithe et al., 2025; Schuurmans et al., 2020; Sinichi et al., 2025). This is particularly relevant when studies need to consolidate datasets from different devices, or when one device is replaced mid‐study due to hardware changes, cost, or availability. Moreover, device‐specific differences in sampling rate, sensor quality, signal processing pipelines, and physical placement may introduce biases or distortions in raw or derived data. Understanding the extent to which devices produce comparable physiological data and identifying which signals are more prone to variation is essential for robust analysis in both clinical and consumer applications (Alfonso et al., 2022; Schyvens et al., 2025). To address this gap, we present a standardized synchronization and validation pipeline designed to be generalizable across any two devices capturing the same signals. The pipeline performs resampling, temporal alignment, amplitude correction, and normalization, followed by robust agreement analyses. Importantly, it is designed to be accessible for clinicians and researchers without coding expertise, thereby lowering the barrier to applying advanced interoperability checks in psychiatric and medical contexts.

As a case study, we apply this pipeline to compare two widely used research‐grade wearables: the Empatica E4 and the EmbracePlus, using up to 48 h of concurrent recordings. Among the research‐grade wearable devices, the Empatica E4 and Empatica EmbracePlus (Embrace) are widely used in both academic research and applied settings (Coelli et al., 2024; Reithe et al., 2025; Schuurmans et al., 2020; Ronca et al., 2023). Despite their prevalence, few systematic studies have investigated the signal‐level agreement and interoperability between these platforms, a crucial step for ensuring data consistency, especially when devices are used interchangeably in longitudinal or multi‐site studies, or when device transitions occur due to hardware updates, discontinuation, or practical constraints.

We analyze participant‐specific results across all signals and identify patterns of agreement, bias, and spectral alignment, offering both granular insights and broader conclusions on device interchangeability. This study makes several important contributions:

- Presenting a robust signal preprocessing and alignment pipeline, including temporal synchronization and normalization to allow fair comparisons.
- Application of advanced agreement metrics, including Bland–Altman analysis, CCC, and spectral coherence, beyond traditional mean comparisons or correlations.
- Comprehensive multimodal signal comparison between two widely used wearable devices in a real‐world, synchronized setup.
- Identification of modality‐specific agreement trends.
- Insights into inter‐subject variability, highlighting how device agreement is influenced by individual physiology or wearability factors.

The manuscript has two primary objectives. First, it introduces a practical, end-to-end pipeline for synchronizing physiological signals recorded concurrently from different wearable platforms, requiring no prior preprocessing by the user. Second, it empirically validates this pipeline by quantifying signal- and feature-level agreement across multiple physiological modalities using real-world data.

### Materials and methods

#### Dataset

This study utilized a proprietary dataset collected as part of an internal research project conducted at the Hospital Clínic of Barcelona, Spain, under institutional ethical approval HCB/2021/104. Participants were recruited from hospital research staff through internal calls for volunteers. A total of 31 participants took part in this study, comprising 20 women and 11 men. Each participant wore two wearable devices, the EmbracePlus and the E4, simultaneously on their non‐dominant hand. The EmbracePlus device was worn above the wrist bone, while the E4 device was positioned next to it, more proximal to the torso. Participants were instructed to wear the devices continuously for up to 48 hours, constrained by E4 battery life. Devices were removed during water‐related activities such as bathing or swimming to prevent data loss and potential damage.

Each session was uniquely identified for both devices, and session start times were recorded. The dataset includes continuous multimodal physiological data (blood volume pulse, BVP, electrodermal activity, EDA, accelerometer, ACC, temperature, TEMP) from both devices, as well as associated demographic metadata such as date of birth, sex, gender identity, and ethnicity. The majority of participants self‐identified as Caucasian. Serial numbers and session codes are included for traceability and were used to align and synchronize recordings between the two devices in the alignment pipeline. This dataset is not currently publicly available due to privacy and institutional restrictions. However, access to the dataset is granted through options provided in the data availability statement.

#### Overview of the validation

The proposed pipeline evaluates cross-device consistency across four complementary levels, designed to progressively assess whether physiological signals recorded from different wearable platforms can be meaningfully integrated. First, temporal alignment assesses whether signals can be synchronized in time despite device-specific clocks, sampling rates, and delays. Second, signal-level agreement evaluates similarity in waveform structure and amplitude after synchronization using metrics such as correlation, RMSE, Bland–Altman analysis, and concordance. Third, feature-level consistency examines whether commonly used physiological features (e.g., HRV, SCR statistics, movement summaries) extracted from aligned signals yield comparable values across devices. Finally, frequency-domain coherence assesses whether signals share similar spectral content across relevant frequency bands. Together, these layers provide a structured and modality-agnostic framework for validating cross-platform physiological data.

While van Lier et al. (2020) proposed a validity protocol centered on temporal synchronization accuracy, the present work extends this framework by systematically evaluating whether synchronized signals also exhibit agreement in waveform structure, spectral content, and derived features across devices. In this sense, temporal alignment is treated as a necessary but not sufficient condition for cross-platform interoperability.

### Preprocessing of the signals

We developed a custom synchronization and preprocessing pipeline to ensure consistency and time alignment of physiological signals. This pipeline was implemented by our research team specifically for this study. Code used in this work is attached as supplementary material and the code will be made publicly available on GitHub at INTREPIBD/e-plus-integration upon publication. The synchronization pipeline involved signal preprocessing to ensure consistency and quality of data from both the Empatica E4 and Embrace Plus devices. These devices were selected to serve as a case study for demonstrating our generalizable synchronization pipeline. Although the pipeline was developed and tested on these two wearables, it is adaptable to any pair of devices capturing the same physiological signals. Device‐specific parameters (e.g., sampling frequency, sensor resolution, filtering options) can be modified as needed, making the pipeline flexible across diverse hardware configurations.

**Fig. 1:**
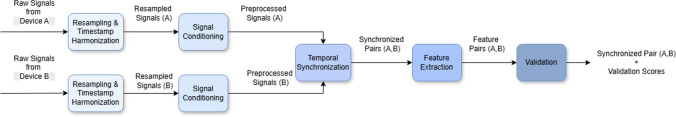
Provides an overview of the proposed synchronization and validation pipeline, illustrating the sequence of processing steps from raw sensor inputs to aligned signals and agreement metrics

The preprocessing pipeline, as shown in Fig. 1, included resampling, baseline alignment, normalization, detrending, and filtering. Importantly, the objective of this preprocessing was not to maximize signal cleanliness or denoise aggressively, but rather to enhance temporal synchronizability between devices while preserving the inherent characteristics and variability of the raw physiological signals. This was crucial to maintaining the integrity of device‐specific signal features that may be informative for downstream interoperability analysis, where device‐dependent differences in waveform shape, amplitude, or timing may carry meaningful information.

### Resampling

All raw signals were resampled to standardized target frequencies, provided by the Empatica, to enable downstream alignment and comparison. We selected common resampling frequencies based on previous studies (Empatica Inc., 2014; Meijer et al., 2023; Braithwaite et al., 2015; Geršak and Drnovšek, 2020; Boucsein, 2012). We used 32 Hz for the accelerometer (ACC) signal, 4 Hz for the electrodermal activity (EDA) and temperature (TEMP) signals, and 64 Hz for the blood volume pulse (BVP) signal.

The target frequencies were based on Empatica E4 specifications and previous literature (Empatica Inc., 2014; Meijer et al., 2023). For accelerometry, three orthogonal axes were retained. According to the Empatica device specifications, the *X*-axis corresponds to the lateral (left–right) direction of the device, the *Y*-axis corresponds to the longitudinal direction along the wrist/forearm, and the *Z*-axis corresponds to the dorsoventral axis, perpendicular to the skin surface (i.e., normal to the wrist). These axes are defined in the device coordinate frame and depend on the device's wrist orientation. Moreover, although skin temperature is a slow‐varying signal, a target sampling frequency of 4 Hz was selected to ensure temporal alignment across modalities and consistency with Empatica E4 data specifications. Importantly, this resampling does not imply increased physiological temporal resolution, as subsequent band‐pass filtering (0.02–0.4 Hz) constrains the signal to its relevant dynamic range. Thus, the higher sampling rate serves alignment purposes only and does not introduce artificial high‐frequency content. Table 1 shows the device specifications and Table 2 summarizes the original sampling rates of both devices and the resampling targets used in our preprocessing pipeline.

**Table 1:** Photoplethysmography (PPG) sensor specifications for EmbracePlus and E4 devices

Device	EmbracePlus	Empatica E4
Type	Custom‐made by Empatica (multi‐channel, multiwavelength)	Commercial de vice
Channels	4 acquisition channels (via 8 photodiodes)	1 channel (via 2 photodiodes)
Wavelengths	Red, infra-red, green (via 9 LEDs)	Green (2 LEDs), Red (2 LEDs)
Sampling rate	26–208 Hz	64 Hz (Non‐ customizable)
Photodiodes	8 photodiodes	2 photodiodes

Photoplethysmography (PPG) hardware layouts from device documentation. Despite substantial hardware differences—EmbracePlus uses 8 photodiodes, E4 uses 2—their BVP signal agreement remained high. (Empatica S.r.l., 2015; Empatica, 2021).

**Table 2:** Original and target sampling frequencies for each signal modality across devices

Signal	E4 original (Hz)	EmbracePlus original (Hz)	Resampled target (Hz)
Accelerometer (ACC)	32	64	32
Electrodermal activity(EDA)	4	4	4
Blood volume pulse (BVP)	64	128	64
Temperature (TEMP)	4	1	4

For resampling, signals were temporally reindexed using pandas’ time-based resampling. When upsampling lower-frequency signals (e.g., EmbracePlus temperature from 1 to 4 Hz), linear interpolation was applied to preserve smooth temporal transitions without introducing stepwise artifacts associated with zero-order hold methods. When downsampling higher-frequency signals, values were aggregated using time-bin averaging (mean), which provides implicit smoothing prior to decimation. No extrapolation beyond observed timestamps was performed. Any remaining high-frequency components were subsequently removed during modality-specific band-pass filtering (see Section 2.2.2), ensuring that signals were constrained to their physiologically relevant frequency ranges.

### Signal conditioning

After resampling, we applied a standardized set of signal conditioning steps uniformly across all four signal types (ACC, EDA, BVP, TEMP) for both Empatica E4 and EmbracePlus devices. The goal was to improve comparability and prepare the signals for alignment and statistical evaluation. The conditioning steps were as follows:

First, *baseline alignment* was performed. Each signal was centered by subtracting its mean value, resulting in zero-mean signals. This step is required to ensure valid sample-wise correlation and concordance analyses following temporal alignment. (Costantini et al., 2023; Xu et al., 2007; Liang & Lo, 2018). Subsequently*, z-score normalization* was applied by dividing each centered signal by its standard deviation, resulting in signals with zero mean and unit variance. This facilitates fair comparison of signal variability and ensures that correlation- and agreement-based metrics (e.g., Pearson correlation, CCC) are not biased by scale differences across devices. This is a common step in wearable and bio signal analysis to standardize amplitude and variability across sessions (Foltyn et al., 2024; Daza et al., 2023). Tanaka et al. (Tanaka et al., 2022) have shown that this improves comparability in sliding window analyses.

Next, *detrending* was performed to remove slow baseline drift while preserving physiologically meaningful dynamics. Long-term trends were removed using rolling mean subtraction. For each modality, we computed a centered moving average using a rectangular window and subtracted it from the signal (partial windows allowed at boundaries). Window lengths were specified in samples to match the resampled data streams: BVP: 600 samples, ACC: 300 samples, EDA: 300 samples, TEMP: 300 samples. Window lengths were selected to match modality-specific time scales: shorter windows (~10 s) for BVP and ACC to remove rapid baseline/offset drift while preserving fast cardiac and movement dynamics, and longer windows (~75 s) for EDA and temperature to remove slow tonic/thermal drift without attenuating phasic SCR events or short-term temperature fluctuations. For BVP in particular, this detrending step primarily stabilizes baseline wander prior to peak/feature extraction; importantly, subsequent band-pass filtering (0.5–4 Hz) removes low-frequency PPG amplitude modulations (e.g., respiratory/vasomotor components) by design, so detrending does not aim to preserve these slow variations. (Xu et al., 2007; Romero et al., 2018; Menghini et al., 2019; Haddadi et al., 2017).

Finally, a third‐order *Butterworth bandpass filter* was applied in a zero‐phase manner (using forward and reverse filtering) to avoid phase distortions and remove noise outside the physiological frequency bands. This is in line with prior work in wearable systems (Elgendi, 2012; Zong et al., 2003). Filter parameters were tuned per signal type:

- *ACC bandpass filtering parameters:* Sampling frequency = 32 Hz, Low cut‐off = 0.5 Hz, High cut‐off = 15 Hz (Föll et al., 2021). For accelerometry, a band-pass filter of 0.5–15 Hz was applied. Although the upper cutoff lies close to the Nyquist frequency (16 Hz at 32-Hz sampling), this choice preserves high-frequency wrist motion components while remaining within stable filter limits. In practice, most human movement energy is concentrated below 10–12 Hz, and subsequent analyses confirmed stable spectral behavior without edge-related artifacts.(Staunton et al., 2026; Pantelopoulos & Bourbakis, 2013)
- *EDA bandpass filtering parameters:* Sampling frequency = 4 Hz, Low cut‐off = 0.05 Hz, High cut‐off =1 Hz (Cowley et al., 2014; Van Bruinessen et al., 2016). EDA signals were band-pass filtered using a zero-phase third-order Butterworth filter with cut-off frequencies of 0.05–1.0 Hz at a sampling rate of 4 Hz. The upper cut-off was selected to remain well below the Nyquist frequency (2 Hz) while preserving all physiologically meaningful phasic electrodermal responses, which predominantly occur below 1 Hz.
- *BVP bandpass filtering parameters:* Sampling frequency = 64 Hz, Low cut‐off = 0.5 Hz, High cut‐off = 4.0 Hz (Elgendi, 2012; Zong et al., 2003; Allen, 2007; Liang & Lo, 2018). This choice of cutoff frequencies is common in HRV analysis, as it allows the retention of the typical frequency range of the heart rate (i.e., 0.5–4.0 Hz), effectively filtering out noise from higher frequency components and low‐frequency trends unrelated to heart rate variability. The filtering procedure introduces a conservative adjustment to the data by smoothing out very low‐frequency variations that are not directly related to the heart rate itself, making peak detection easier and more robust. While the trade‐off involves a downward bias in HRV measures (due to the reduced variability in IBIs), this is acceptable in the context of our analysis, where the primary aim was to clean and synchronize the data across different physiological signals (BVP, EDA, ACC, etc.) for further analysis, by focusing on stable, synchronized metrics, rather than capturing the fine variations in HRV that occur below 0.5 Hz.

While very low-frequency PPG components (e.g., around 0.1 Hz) have been reported as informative in specific contexts such as mental workload assessment or long-term autonomic regulation, these dynamics occur on time scales beyond the scope of the present study. Our primary objective was short-to-medium timescale synchronization and cross-device comparability rather than characterization of circadian or ultra-low-frequency cardiovascular rhythms. Accordingly, low-frequency components below 0.5 Hz were intentionally attenuated as a conservative preprocessing choice to ensure stable alignment and feature extraction across modalities.

- *TEMP bandpass filtering parameters:* Sampling frequency = 4 Hz, Low cut‐off = 0.02 Hz, High cut‐off = 0.4 Hz (Vert et al., 2022). For temperature specifically, upsampling from 1 Hz to 4 Hz was performed solely to match the common time base across modalities/devices for synchronization. Skin temperature varies slowly relative to these sampling rates; therefore, linear interpolation does not introduce physiologically meaningful high-frequency content. Importantly, subsequent temperature band-pass filtering (0.02–0.4 Hz) restricts the signal to minute-scale dynamics, thereby attenuating any potential interpolation-induced high-frequency components. Thus, upsampling increases temporal grid density but does not increase the effective temporal resolution of the temperature signal.

### Alignment

Following preprocessing, each physiological signal (EDA, BVP, TEMP, ACC) from the Empatica E4 and Embrace Plus devices was subjected to a multi-stage alignment pipeline designed to address temporal misalignment and amplitude inconsistencies. The alignment process combined dynamic time warping (DTW), wavelet‐based amplitude correction, and cross‐correlation evaluation.

Signals were first temporally aligned using dynamic time warping (DTW), a technique that identifies an optimal non‐linear mapping between time series by minimizing the cumulative distance between signal amplitudes (Chen et al., 2020; Steinmetzer et al., 2020; Wang, 2023). DTW computes an alignment path that adjusts for local time shifts and signal delays, enabling robust comparison even in the presence of temporal asynchronies. DTW produces an alignment path between the two signals that pairs samples based on similarity, which initially yields aligned value–index pairs rather than uniformly sampled time series. The resulting warping path was used to align signals from the two devices frame by frame. To restore temporal interpretability, timestamps from both devices were projected onto the DTW alignment path and subsequently regularized to obtain a uniformly spaced time axis. For downstream analyses and visualization, the resolved timestamp sequence of Signal 2 was used as the common temporal reference. This procedure adjusts the time representation without modifying signal amplitudes, resulting in aligned signals expressed as value–time pairs on a shared temporal grid.

Dynamic time warping (DTW) offers substantial advantages for synchronizing time series data collected from real‐world devices, particularly when measurements are asynchronous, noisy, or exhibit temporal distortions. Unlike simpler methods such as Euclidean distance or Pearson correlation, DTW allows non‐linear alignment, enabling it to compensate for device clock drift, transient data loss, or inter‐device variability in signal dynamics (Ratanamahatana & Keogh, 2005; van Schooten et al., 2023).

While research‐grade wearables nominally sample at fixed frequencies, effective irregularities may arise due to dropped data packets, buffering delays, or temporary sensor disconnections in uncontrolled environments (Tormene et al., 2009). DTW is especially robust to such issues, as it can align sequences even when their timestamps are partially misaligned (Meng et al., 2022), making it ideal for fusing heterogeneous sensor modalities (Lee et al., 2019). Empirical studies have shown that DTW achieves superior similarity detection across domains such as human motion analysis, physiological signal comparison, and neuroimaging (Imaizumi et al., 2020; Salvador & Chan, 2007). The aligned timestamps were further refined to ensure even temporal spacing, resolving repeated timestamp values. To quantify similarity, Pearson correlation coefficients were computed before and after alignment. This provided insight into both linear and monotonic relationships across devices for each modality.

Physiological signals collected from different devices often exhibit amplitude scaling differences and baseline shifts due to variations in sensor hardware, placement, or calibration. Even after temporal alignment using DTW, such discrepancies can hinder accurate comparison across paired signals. To address this, we performed post‐alignment amplitude normalization using the discrete wavelet transform (DWT), which allows multi‐resolution decomposition of signals without introducing temporal distortion.

Let **s**_1_, **s**_2_ ∈ R^*T*^ denote two time‐aligned signals of equal length *T*, corresponding to the same modality (e.g., BVP) from two devices. We applied DWT to each signal using the Daubechies 4 (db4) wavelet basis at decomposition level 2 (Zhou et al., 2024), yielding wavelet coefficient vectors **c**_1_, **c**_2_ ∈ R^*N*^, where *N* is the total number of coefficients. To equalize amplitude profiles, we partially shifted the wavelet coefficients toward a shared midpoint between the two signals. The adjusted coefficients ${c{\prime}}_{1}{c{\prime}}_{1}$ were computed as (Gao et al., 2019):

$$\begin{array}{c}{c{\prime}}_{1}={c}_{1}+\gamma .({\mu}_{2}-{\mu}_{1})\\ {c{\prime}}_{2}={c}_{2}-\gamma .({\mu}_{2}-{\mu}_{1})\end{array}$$

where:

· *γ* = 0.5 is a blending factor controlling the strength of the adjustment,

· $\mu_{1} = \frac{1}{N}\sum_{i=1}^{N}{c}_{1}[i]$ and $\mu_{2} = \frac{1}{N}\sum_{i=1}^{N}{c}_{2}[i]$ are the mean values of the wavelet coefficients for each signal.

Finally, we reconstructed the adjusted signals ${s{\prime}}_{1}$ and ${s{\prime}}_{2}$ using the inverse DWT applied to ${c{\prime}}_{1}$ and ${c{\prime}}_{2}$, respectively. This wavelet‐based adjustment preserved temporal structure while correcting for phase and amplitude mismatches across devices (Zhou et al., 2024; Gao et al., 2019).

To limit memory usage during DTW/DWT alignment, signals were processed in chunks of 10,000 samples. Chunking was performed after resampling and temporal merging, such that, for a given modality, both devices shared the same sampling rate and timestamp grid within each chunk. As a result, each chunk corresponds to a fixed time interval for that modality, while chunk duration in seconds differs across modalities due to their different sampling frequencies (e.g., longer intervals for EDA/TEMP than for BVP). Importantly, within each modality, alignment and correlation were always performed over identical time spans across devices. This approach reduced memory consumption while preserving fine‐grained alignment resolution.

Cross‐correlation magnitudes and lags were inspected for each signal modality (e.g., BVP, EDA, ACC, TEMP) across paired recordings from the Empatica E4 and EmbracePlus. This analysis was used to verify temporal overlap and identify consistent lead/lag patterns between the devices (van Lier et al., 2020). Cross-correlation was computed directly on the aligned, detrended, and band-pass-filtered signals using a rectangular window (i.e., no additional tapering). Preliminary tests with Hann-windowed segments produced comparable lag estimates, confirming that edge effects did not materially influence the results. The influence of sequence edges was mitigated through prior signal conditioning, DTW-based temporal alignment, and chunked processing, such that cross-correlation was evaluated on locally stationary segments rather than on full-length recordings.

### Feature extraction

To assess signal similarity not only at the raw data level but also in derived signal characteristics, we extracted features from each aligned modality across the Empatica E4 and Embrace Plus devices. Feature extraction was modality‐specific and leveraged domain‐relevant libraries, including FLIRT (Föll et al., 2021) for statistical and accelerometry features, and NeuroKit2 (Makowski et al., 2021) for physiological signal processing (EDA, BVP). Features were extracted in a sliding window fashion (60-s window length, 1-s step size), and pairwise feature correlations were computed to quantify cross‐device similarity. While chunking during alignment operates in the sample domain for computational reasons, feature extraction is performed in the time domain using physiologically motivated window lengths (Boucsein, 2012; Schaffer & Vagedes, 2013).

Because DTW-based synchronization was applied prior to feature extraction, corresponding windows across devices represent the same nominal time intervals. Pairwise feature correlations were therefore computed at zero lag to quantify cross-device similarity, without introducing additional temporal offsets.

No aggressive outlier detection or removal was applied. This was a deliberate choice, as transient deviations and abrupt changes in physiological signals can serve as informative event markers for cross-device synchronization. Instead of removing such events, we relied on minimal but consistent signal conditioning (resampling, detrending, band-pass filtering, and normalization) to reduce noise and baseline drift while preserving transient dynamics. This approach balances robustness for feature extraction and statistical analysis with the preservation of physiologically meaningful events relevant for temporal alignment. For clarity, feature descriptions are presented in the same modality order as used throughout the Methods and Results sections.

For blood volume pulse (BVP) signals, we employed the ppg_process() and ppg_analyze() functions from NeuroKit2. Signals were segmented into 60‐s chunks, processed to extract peaks, heart rate variability (HRV) metrics, and amplitude-based features (Makowski et al., 2021; Elgendi et al., 2013; Schafer & Vagedes, 2013; Castaldo et al., 2019; Selvaraj et al., 2020; Voss et al., 2009; Bernardi et al., 2001; Kim et al., 2020). The extracted features are in Table 3:

**Table 3:** Extracted heart rate variability (HRV) features from BVP signal

Category	Features
Time‐domain measures	HRV_MeanNN,HRV_IQRNN, HRV_MaxNN, HRV_SDNN,HRV_SDANN,HRV_SDSD, HRV_pNN50
Geometric measures	HRV_TINN, HRV_HTI, HRV_PRC20NN
Frequency‐ domain measures	HRV_LF, HRV_LFn, HRV_LnHF, HRV_HF, HRV_VLF, HRV_ULF
Nonlinear dynamics	HRV_SD1, HRV_SD2, HRV_CSI, HRV_CVI, HRV_AI, HRV_C1a, HRV_C2a, HRV_Cd, HRV_CD
Entropy measures	HRV_SampEn, HRV_RCMSEn, HRV_MSEn
Fractal/complexity	HyRV_DFA_*, HRV_MFDFA_alpha*, HRV_HFD, HRV_GI
Other Features	HRV_PASS, HRV_PIO

Acronyms and their explanations:

HRV_MeanNN: Mean of NN intervals, HRV_IQRNN: Interquartile Range of NN intervals, HRV_MaxNN: Maximum NN interval, HRV_SDNN: Standard deviation of NN intervals, HRV_SDANN: Standard deviation of the average NN intervals, HRV_SDSD: Standard deviation of successive differences, HRV_pNN50: Proportion of successive NN intervals differing by more than 50 ms. HRV_TINN: Triangular interpolation of NN intervals, HRV_HTI: Heart rate turbulence index, HRV_PRC20NN: Proportion of NN intervals greater than 20 ms. HRV_LF: Low frequency power (0.04–0.15 Hz), HRV_LFn: Normalized low frequency, HRV_LnHF: Log‐transformed ratio of low frequency to high frequency, HRV_HF: High frequency power (0.15‐0.4 Hz), HRV_VLF: Very low frequency power (<0.04 Hz), HRV_ULF: Ultra‐low frequency power. HRV_SD1: Standard deviation of the Poincaré plot’s short axis, HRV_SD2: Standard deviation of the Poincaré plot’s long axis, HRV_CSI: Complexity index, HRV_CVI: Cardiovascular variability index, HRV_AI: Autonomic index, HRV_C1a: First component of Poincaré plot, HRV_C2a: Second component of Poincaré plot, HRV_Cd: Correlation dimension, HRV_CD: Complexity dimension

HRV_SampEn: Sample entropy, HRV_RCMSEn: Recursive cumulative sum entropy, HRV_MSEn: Multiscale entropy. HRV_DFA_*: Detrended fluctuation analysis, HRV_MFDFA_alpha*: Multifractal DFA alpha exponent, HRV_HFD: High frequency DFA, HRV_GI: Gait index. HRV_PASS: Peak amplitude skewness, HRV_PIO: Peak intensity oscillations

Electrodermal activity (EDA) signals were processed using nk.eda_process(), which performs smoothing, peak detection, and decomposition into tonic and phasic components. Extracted features, shown in Table 4, included skin conductance level (SCL), skin conductance response (SCR) count and amplitude, and signal derivatives (Makowski et al., 2021; Greco et al., 2016; Posada‐Quintero et al., 2016; Braithwaite et al., 2015; Nagai et al., 2004; Roth et al., 2012):

**Table 4:** Extracted features from electrodermal activity (EDA) signal

Category	Features
Signal components	EDA_Tonic, EDA_Phasic
SCR (skin conductance response) Metrics	SCR_Onsets, SCR_Peaks, SCR_Amplitude, SCR_Height, SCR_RiseTime, SCR_Recovery, SCR_RecoveryTime

Acronyms and their explanations:

EDA_Tonic: Tonic component of the electrodermal activity signal, EDA_Phasic: Phasic component of the electrodermal activity signal. SCR_Onsets: The onset of the skin conductance response, SCR_Peaks: The peak of the skin conductance response, SCR_Amplitude: Amplitude of the skin conductance response, SCR_Height: Height of the skin conductance response, SCR_RiseTime: Time taken for the SCR to rise to its peak, SCR_Recovery: The recovery phase of the SCR, SCR_RecoveryTime: The time it takes for the SCR to return to baseline

Accelerometer signals (single axis or magnitude) were processed using Flirt’s get_acc_features() function. This included time‐ and frequency‐domain features, as shown in Table 5, activity metrics, and acceleration patterns relevant for movement analysis (Föll et al., 2021):

**Table 5:** Extracted time‐domain features

Category	Features
Basic stats	Mean, Std, Min, Max, PTP, sum
Shape	Skewness, kurtosis, IQR, IQR_5–95_, Pct_5_, Pct_95_
Dynamics	RMS, line integral, peaks, *n*_above_ _mean_, *n*below mean, *n*sign changes
Entropy	Shannon, permutation, SVD

Acronyms and their explanations:

PTP: peak‐to‐peak, IQR: interquartile range, IQR_5–95_: 5th–95th percentile range, Pct_5_: 5th percentile, Pct_95_: 95th percentile, RMS: root mean square, SVD: singular value decomposition. n_above mean: Number of values above the mean, n_below mean: Number of values below the mean, n_sign changes: Number of sign changes. Shannon: Shannon entropy, Permutation: permutation entropy

Temperature features, as shown in Table 6, were computed using the Flirt library’s get_stat_features() function (Föll et al., 2021). Time‐indexed temperature series were created using a 4-Hz sampling rate. For both Embrace and E4 devices, the following statistical features were extracted within fixed‐length windows:

**Table 6:** Extracted temperature features

Category	Features
Basic stats	Mean, Std, Min, Max, PTP, sum
Shape	Skewness, kurtosis, IQR, IQR_5–95_, Pct_5_, Pct_95_
Dynamics	RMS, line integral, peaks, *n*above mean, *n*below mean, *n*sign changes
Entropy	Shannon, permutation, SVD

Acronyms and their explanations:

PTP: peak‐to‐peak, IQR: interquartile range, IQR_5–95_: 5th–95th percentile range, Pct_5_: 5th percentile, Pct_95_: 95th percentile, RMS: root mean square, SVD: singular value decomposition

### Statistical analysis and testing

To rigorously evaluate signal similarity between the devices, we employed a comprehensive battery of statistical tests and visual assessments. These covered signal‐level alignment, feature consistency, agreement metrics, and frequency‐domain coherence.

- *Visual inspection*: Aligned signals for each modality were plotted side‐by‐side and overlaid to visually assess temporal agreement. This step provided initial qualitative evidence for successful synchronization, identifying gross misalignments or amplitude discrepancies (van Lier et al., 2020).
- *Bland–Altman analysis*: To assess the agreement between the signals from both devices, we computed Bland–Altman plots and derived summary statistics (mean bias and limits of agreement). This method quantifies the extent and pattern of disagreement between paired measurements (Greco et al., 2016; van Lier et al., 2020). The analysis is based on comparing the difference between the two measurements (i.e., the difference between the values from each device) to their average. Mathematically, let *x*_*i*_ and *y*_*i*_ represent the measurements from the two devices for the *i*‐th sample. The difference between the paired measurements is given by:
  $${d}_{i}={x}_{i}-{y}_{i}$$

The mean bias is the average of the differences, calculated as:

$$Mean bias= \frac{1}{N}\sum_{i=1}^{N}{d}_{i}$$

where *N* is the total number of paired samples. The limits of agreement (LoA) are computed as the mean bias ±1.96 × standard deviation of the differences, i.e.,

$$\mathrm{L}\mathrm{o}\mathrm{A}= \mathrm{M}\mathrm{e}\mathrm{a}\mathrm{n} \mathrm{b}\mathrm{i}\mathrm{a}\mathrm{s}\pm 1.96\times \mathrm{S}\mathrm{D}\left({d}_{i}\right)$$

The method assumes that the differences, *d*_*i*_, are normally distributed, and it provides insight into whether there is any systematic bias between the devices. The limits of agreement represent the range within which 95% of the differences between paired measurements are expected to lie. This statistical approach helps assess the degree of agreement and whether any systematic bias or discrepancies exist between the two devices.

- *Phase synchronization*: Phase synchronization between signals was quantified using metrics derived from analytic signal decomposition. These capture temporal phase alignment, especially relevant for periodic or rhythmic signals like ACC and BVP (Gao et al., 2019).
- *Concordance correlation coefficient (CCC):* The concordance correlation coefficient (CCC) was computed after z‐score normalization to measure agreement in both precision (How closely the values of the two synchronized signals align) and accuracy (How well the synchronized signals agree in terms of their underlying temporal patterns). CCC plots were generated using identity lines to visualize deviation from perfect concordance (van Lier et al., 2020).
- *Amplitude agreement*: Signal‐level amplitude agreement was quantified using: root mean square error (RMSE) mean absolute error (MAE). These metrics provided direct measurement of average amplitude deviation between aligned signals (Gao et al., 2019). In this case, since there is no external trusted reference or ground truth signal, the amplitude agreement is assessed by comparing the two signals directly with each other. These metrics provide a direct measure of average point-wise amplitude deviation between aligned signals. Because no external ground-truth reference signal is available, RMSE and MAE reflect relative amplitude consistency between devices rather than absolute accuracy. Importantly, amplitude differences may arise from device-specific calibration or gain differences; therefore, RMSE and MAE were interpreted in conjunction with shape-based agreement metrics (e.g., correlation coefficients, concordance measures, and DTW alignment), which are more sensitive to the similarity of temporal patterns than absolute signal scaling.
- *Statistical divergence*: To assess the distributional similarity of signal amplitudes, we computed Kullback–Leibler (KL) divergence between the normalized histograms (PDFs) of each aligned signal. This measure quantifies how one probability distribution diverges from a second, expected distribution (van Lier et al., 2020).
- *Feature correlation analysis*: After feature extraction (Section 2.4), we identified the strongest feature‐level associations between devices. The feature correlation matrix was flattened, sorted, and thresholded (|*r*|>0.7) to extract the most consistent features across devices. This provided a summary of which physiological features are most robust to sensor differences (van Lier et al., 2020).
- *Frequency‐domain coherence*: We applied coherence analysis to signals to quantify synchronization across frequency bands. Coherence values,${C}_{xy}\left(f\right)$, were plotted over a range of frequencies, with a summary extracted over a targeted frequency band (e.g., 0.1–10 Hz) (Gao et al., 2019).
- *Mutual information*: To assess nonlinear dependencies, we computed mutual information (MI) and normalized mutual information (NMI) across a range of histogram bin sizes. The optimal bin size was selected using the elbow method (Kneedle algorithm). MI and NMI were then interpreted on a scale of dependency strength. NMI values were interpreted similarly to assess normalized dependency bounds (Gao et al., 2019).

## Results

We evaluated the alignment quality across Empatica E4 and EmbracePlus devices as a case study to demonstrate the proposed synchronization pipeline. The evaluation used a combination of statistical, spectral, and feature‐level analyses. While the pipeline itself is designed to be generalizable across any pair of devices measuring the same signals and their derived biomarkers, the results reported here are specific to the E4–EmbracePlus dataset. In the Appendix Sections 1, we present detailed results from two selected participants as a representative subset of the entire study. These participants were selected for their ability to provide a clear, comprehensive overview of the data from the full cohort of 31 participants. The goal of presenting these two participants is to illustrate the patterns observed across the broader sample while highlighting key findings that reflect the overall trends of the larger dataset. The selection of these two participants was not arbitrary. They were selected to encompass different aspects of the variability observed across the cohort. By showcasing their data, we can effectively demonstrate the general trends, variations, and insights that are consistent with the group as a whole.

### Blood volume pulse signal agreement

To validate our synchronization pipeline, we compared the raw and derived BVP signals recorded simultaneously by the EmbracePlus and Empatica E4 devices. The Pearson correlation matrix of key BVP‐derived features (time‐, frequency‐, and nonlinear HRV metrics), as shown in Figure A1 (in Appendix); most matching features exhibit correlations above 0.90, and even the more noise‐sensitive complexity measures (e.g., Sample Entropy, DFA _*α*_, MSEn) exceed 0.75, confirming that our preprocessing and alignment preserve both temporal and spectral properties across devices. Frequency components such as HRV_LF, HRV_HF, HRV_VLF, and HRV_ULF show strong correlations (*r* > 0.85), further implying that the spectral power distribution of the derived HRV signals is preserved across devices.

**Fig. 2:**
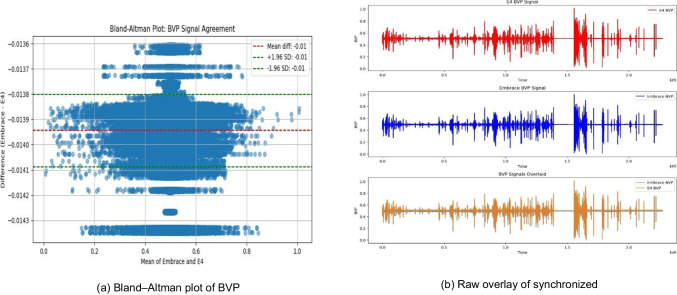
Agreement between EmbracePlus and Empatica E4 BVP signals: **(a)** sample‐level Bland–Altman analysis showing bias and limits of agreement; **(b)** overlaid raw BVP time series after synchronization

Bland–Altman analysis (as shown in Fig. 2A and in more detail in Appendix Figure A2) quantifies agreement at the sample level. Across 30 paired recordings (one per participant), the mean bias was essentially zero (– 0.01 normalized units) with very narrow limits of agreement (± 0.0015), yielding a percentage error of approximately 0.6%, well below the 30% threshold commonly used in clinical PPG validation studies. These tight bounds, together with the absence of proportional bias, indicate that any residual differences (e.g., due to pulse transit delays from proximal versus distal sensor placement) are physiologically negligible in the context of normalized BVP amplitudes.

Further, concordance correlation coefficient (CCC) identity plots (as shown in the Appendix Figure A3) and spectral coherence estimates (in Appendix Figure A4) reinforce these findings. The CCC between devices approached 1.000 over the entire cohort of 31 paired recordings, demonstrating virtually perfect linear and scale agreement, while coherence remained above 0.97 across the 0–32-Hz band (and above 0.98 within the cardiac band of 0.5–5 Hz). Finally, the two raw and their overlaid traces (as shown in Fig. 2B and in more detail in Appendix Figure A5) illustrate minimal lag or distortion in waveform morphology following synchronization.

### Electrodermal activity signal aggreement

To assess the synchronization pipeline for skin conductance, we compared raw and derived EDA signals from the EmbracePlus and Empatica E4 devices. Figure B6 (in Appendix) shows the Pearson correlation matrix of key EDA features (tonic/phasic decompositions and SCR metrics). Tonic and phasic components of the EDA signal correlate moderately to strongly (tonic: *r* ≈ 0.60–0.70; phasic: *r* > 0.85), while event‐based SCR features (e.g. SCR_Height, SCR_Amplitude, SCR_Peaks) achieve *r* > 0.7, confirming that both baseline and transient conductance fluctuations are largely preserved across devices. The slight reduction in correlation between embrace_EDA_Tonic and e4_EDA_Tonic compared to raw or phasic components may still point to differences in baseline correction or filtering; however, additional factors likely contribute.

The main goal of preprocessing was not to remove all artifacts, but rather to retain the original signal structure while improving the visibility of shared temporal patterns between the two devices. The stronger correlation between the phasic components of the two devices can be explained by the fact that both devices may have similar response patterns to the same types of artifacts (e.g., motion artifacts, electrical interference). Since the phasic component is typically much more dynamic and higher frequency (reflecting short‐term fluctuations), the artifacts might be present in both devices, but in a similar temporal structure. In contrast, the tonic component, which represents the long‐term baseline conductance, requires higher precision in capturing small, sustained variations over time. This makes it more sensitive to slight differences in how each device captures and processes this component.

Moreover, the Empatica E4 was worn more proximal to the wrist, whereas the EmbracePlus was positioned more distally. This placement configuration was consistent across all paired recordings, and no counterbalancing was performed. Sweat gland density is known to decrease with increasing distance from the palm, meaning fewer sweat glands are typically activated at more distal sites (Bariya et al., 2020; Wilke et al., 2007). As a result, both devices detect phasic peaks, reflecting dynamic increases in sweat, but the tonic (mean) level differs simply because the baseline sweat volume is lower at more distal sites. Device hardware differences, such as electrode composition and contact area, can alter tonic baseline conductance independent of phasic responsiveness.

**Fig. 3:**
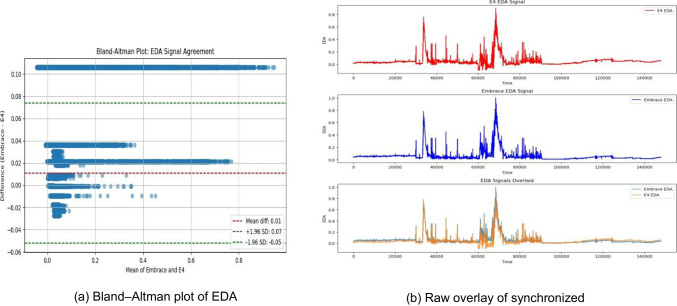
Agreement between EmbracePlus and Empatica E4 EDA signals: **(a)** sample‐level Bland–Altman analysis showing bias and limits of agreement; **(b)** overlaid raw EDA time series after synchronization

Bland–Altman analysis (shown in Fig. 3A and in more detail in Appendix Figure B7) reveals a mean bias of + 0.01 normalized units with limits of agreement from – 0.05 to + 0.07, corresponding to a percentage error of ≈ 26 within the ± 30% clinical threshold for acceptable PPG/EDA agreement. It is also worth noting that if the E4 wristband is less breathable, it may increase perspiration as part of a thermoregulatory response, which could in turn elevate EDA levels. This may partially explain cases in which the E4 shows higher mean EDA values than the EmbracePlus (Appendix Figure B10). The differing form factors and materials of the two devices likely contribute to this effect: the EmbracePlus employs a soft, breathable silicone rubber strap designed for all‐day comfort (Empatica, 2021), whereas the E4 wristband uses a stiffer, less‐permeable proprietary band structure (Empatica, 2020). Such differences in material breathability and enclosure design may influence thermal exchange and moisture accumulation during extended wear. Prior work on wearable temperature sensors also suggests that tightly fitting, non‐breathable enclosures can trap heat and humidity, affecting both wearer comfort and physiological signal measurements.

Concordance correlation coefficient (CCC) identity plots (in Appendix Figure B8) yield overall CCC = 0.776, with per‐sample concordance clustering closely along *y*_=_ *x*. Spectral coherence analysis (in Appendix Figure B9) further confirms signal similarity: coherence remains above 0.90 in the dominant EDA band (< 1 Hz) and averages 0.84 across 0–2 Hz, demonstrating preservation of frequency content despite modest high‐frequency variability. This variability may also, in part, be attributed to hardware factors such as differences in electrode composition between devices. For example, earlier versions of the E4 employed Ag/AgCl electrodes, which may behave differently compared to the materials used in the EmbracePlus (Posada‐Quintero et al., 2017; Empatica S.r.l., 2015; Empatica Support, 2025; Empatica S.r.l., 2021).

Finally, the two overlaid raw traces (shown in Fig. 3A and in more detail in Appendix Figure B10) illustrate minimal timing offsets and matching peak morphology, with slightly smoother tonic trends in the EmbracePlus signal. Taken together, these results indicate that the alignment pipeline yields clinically acceptable agreement for EDA signals across two independent wearable platforms, while highlighting expected variability.

### Accelerometer signal agreement

We evaluated cross‐device agreement of wrist‐worn accelerometry on each axis (see Appendix C).

**Fig. 4:**
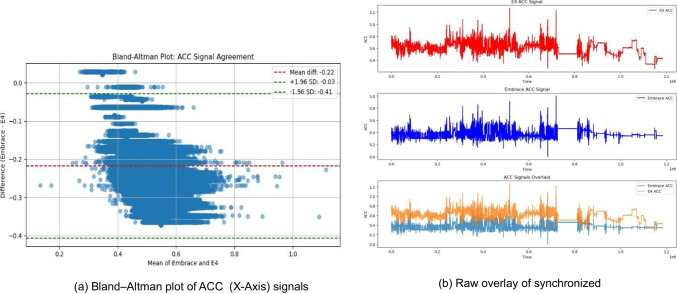
Agreement between EmbracePlus and Empatica E4 ACC signals for the *X*-axis: **(a)** sample‐level Bland–Altman analysis showing bias and limits of agreement; **(b)** overlaid raw ACC time series after synchronization

On the *X*‐axis (in Appendix Figure C11), moderate feature agreement was observed (Pearson *r* = [0.36, 0.7], CCC = 0.60 (in Appendix Figure C13), RMSE = 0.097) with high spectral coherence (in Appendix Figure C19) (0.975), but Bland–Altman analysis (can be seen in Fig. 4A and in more details in Appendix Figure C12) revealed a consistent negative bias (mean diff ≈ – 0.20) and a percentage error of ≈ 40%, exceeding the 30% clinical threshold. While absolute amplitude differs (systematic offset, as shown in raw overlays in Fig. 4B), the relative shape and dynamics of the signals are preserved. This may reflect differences in how the devices respond to high‐frequency motion or short‐term movement mismatches, potentially due to brief periods of loose contact, inconsistent placement, or device handling. While accelerometer signals reflect actual movement, discrepancies between devices may sometimes reflect sensor‐specific sensitivity or mechanical artifacts rather than true differences in physical activity.

**Fig. 5:**
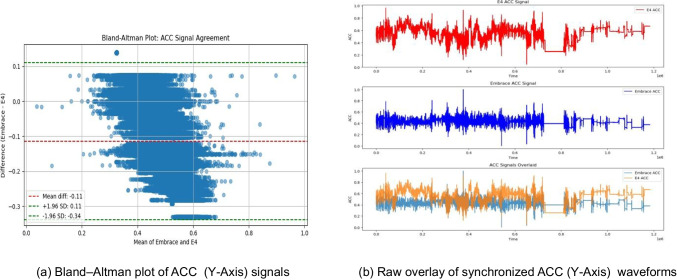
Agreement between EmbracePlus and Empatica E4 ACC signals: **(a)** sample‐level Bland–Altman analysis showing bias and limits of agreement; **(b)** overlaid raw ACC time series after synchronization

The *Y*‐axis (in Appendix Figure C16) showed the weakest correspondence (Pearson *r* = 0.09, CCC = 0.19 C18, RMSE = 0.120) and similar > 40% error, indicating substantial amplitude divergence despite preserved coherence (in Appendix Figure C19 (> 0.97)) and stable bias across motion intensities, as shown in Fig. 5a. This result flags a device‐specific baseline offset or differential sensitivity to lower amplitude motion along the *Y*‐axis (see Fig. 5B). Part of this discrepancy may also stem from positional differences: the E4 was worn more proximally (closer to the forearm), while the EmbracePlus was worn distally (nearer to the wrist). For instance, during activities such as typing or using a mouse, where most motion is generated at the wrist and hand, the distal, wrist‐level EmbracePlus is expected to capture the resulting accelerations more strongly than the more proximal E4, potentially contributing to the observed amplitude divergence along the *Y*‐axis. Prior research on wrist‐worn accelerometers highlights that even slight changes in device position (top vs bottom of the wrist) can significantly affect the measured acceleration vector and activity estimates (Polo et al., 2019; van Hees et al., 2013).

**Fig. 6:**
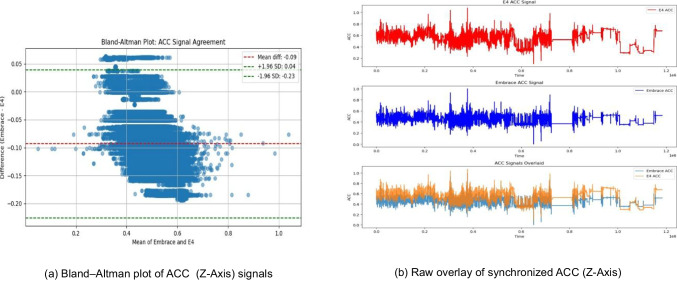
Agreement between EmbracePlus and Empatica E4 ACC signals: **(a)** sample‐level Bland–Altman analysis showing bias and limits of agreement; **(b)** overlaid raw ACC time series after synchronization

These results underscore robust synchronization for device-defined *Z*-axis, but advise caution when interpreting absolute *X* and *Y* accelerations across devices. It should be noted that, for wrist-worn sensors, axis orientations are defined in the local device coordinate frame and do not correspond to a fixed anatomical or global vertical direction, as wrist rotation and task-dependent movements can substantially alter axis alignment. Despite this, the *Z*‐axis achieved the strongest alignment (Pearson *r* = 0.81, CCC = 0.85, RMSE = 0.064, coherence = 0.988) with a percentage error of ≈ 28.5%, narrowly within acceptable bounds, also supported by the Bland–Altman plots in Fig. 6A. Identity‐plot analyses (in Appendix Figures C13, C18, and C23) and overlaid time‐series traces (in Appendix Figures C15, C20 and C25) confirm that all axes maintain consistent temporal and spectral patterns, peaks and bursts align closely, while highlighting axis‐dependent amplitude offsets attributable to differences in device orientation, placement, and sensor characteristics, as shown in Fig. 6B. Accordingly, stronger agreement along the *Z*-axis reflects greater consistency in device-frame dynamics rather than privileged sensitivity to a globally defined vertical motion.

### Skin temperature signal agreement

**Fig. 7:**
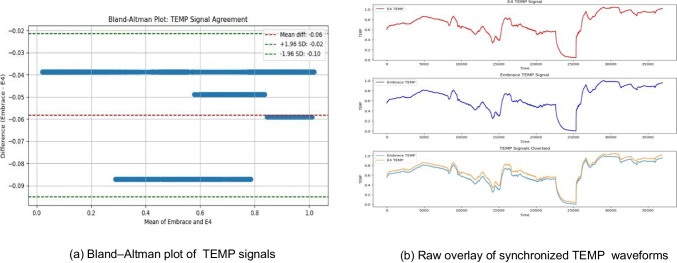
Agreement between EmbracePlus and Empatica E4 TEMP signals: **(a)** sample‐level Bland–Altman analysis showing bias and limits of agreement; **(b)** overlaid raw TEMP time series after synchronization

We evaluated cross‐device agreement of wrist‐worn temperature sensors on the EmbracePlus and Empatica E4. Figure D26 (in Appendix) displays the feature‐correlation matrix for basic and entropy‐based temperature metrics: basic statistics (mean, std, RMS) correlate strongly (*r* > 0.8), while complexity measures (permutation and SVD entropy) show moderate agreement (*r* ≈ 0.60–0.70). Bland–Altman analysis (shown in Fig. 7A and in more detail in Figure D27 in the Appendix) yields a mean bias of – 0.06 corresponding to an average percentage error of ≈ 17.5%, well within the ± 30 % clinical threshold. There is a consistent negative bias across paired recordings, meaning EmbracePlus generally reports lower temperature values than E4. The spread is tight and symmetric, indicating stable bias with no major outliers or proportional error.

Concordance correlation coefficient identity plots (in Appendix Figure D28) confirm overall CCC = 0.755, with data clustering tightly along *y* = *x* and a small vertical offset reflecting consistent underestimation by EmbracePlus. Spectral coherence (in Appendix Figure D29) remains above 0.72 across 0–0.5 Hz and exceeds 0.90 in most bands, indicating preserved temporal dynamics despite minor amplitude and distributional offsets (MI = 3.32, NMI = 0.74, KL = 0.49, Pearson *r* = 0.755, PLV = 0.982). Overlaid time‐series traces (shown in Fig. 7B and in more detail in Appendix Figure D30) show closely aligned temperature peaks and troughs across devices. This confirms that both devices capture similar temporal patterns, even if the absolute values differ. Minor dips at very low frequencies likely reflect different filtering behaviors or thermal inertia between the sensors.

The differing form factors and materials of the devices may contribute as well. Prior work on wearable temperature sensors suggests that tight, non-breathable enclosures can trap heat and humidity, leading to elevated or lagging temperature readings compared with more flexible, skin-adaptive designs (Sim et al., 2016). Together, these results demonstrate that, although EmbracePlus reports slightly lower absolute values, both devices reliably capture relative thermal fluctuations and frequency content. Compared with other physiological signals (e.g., accelerometer), temperature readings showed relatively stable agreement, suggesting that thermal sensing is less affected by placement or device-specific factors in this setup.

## Discussion

This study investigated signal‐level agreement between the Empatica E4 and EmbracePlus devices across multiple physiological modalities, focusing on synchronization fidelity and modality‐specific variability. The findings help answer two key research questions:

### How well do the Embrace and E4 devices agree across physiological signals?

Overall, the EmbracePlus and Empatica E4 showed moderate to strong agreement across all signal types, though the degree of reliability varied by modality.

Blood volume pulse (BVP) showed the strongest agreement between devices, with high consistency across both amplitude and temporal features. This strong agreement reflects the relatively rich temporal variability of the BVP signal, which provides informative structure for synchronization and alignment. Although BVP is quasi-periodic, natural variability in heart rate and pulse morphology prevents exact sequence repetition, enabling reliable identification of corresponding events across devices. In contrast to slowly varying signals such as temperature, which provide limited temporal information for precise alignment, BVP contains sufficient dynamic content to yield accurate synchronization. Consequently, small residual timing differences on the order of tenths of a second remain visible in some cases, but are generally negligible for the intended analyses. The agreement between PPG sensors is also remarkable, considering that the Embrace uses 8 photodiodes (page 64 of the user manual) and the E4 uses 2 (Empatica S.r.l., 2015; Empatica, 2021), as shown in Table 1, Section 2.2.1.

Electrodermal activity (EDA) showed generally good agreement between devices, though with slightly more variability than BVP when assessed across paired recordings. Variability in agreement was observed across device pairs, indicating that within-subject factors—such as local skin conductance baseline, wear location, strap fit, or contact stability—can influence cross-device measurement consistency. While overall trends aligned well across individual recordings, these nuances highlight EDA's sensitivity to local measurement conditions and suggest that device comparisons for EDA should explicitly account for subject-level variability. Interestingly, while prior literature suggests that more distal placements, like that of the EmbracePlus, should detect higher EDA due to increased sweat gland density (Bariya et al., 2020; van Dooren et al., 2012), our results do not consistently support this expectation. For some (nine out of 31) paired datasets, EmbracePlus reported lower EDA values than E4, resulting in a noticeable negative bias. However, this pattern was not universal: other paired datasets (the remaining 22 out of the 31) exhibited minimal bias or even a slight positive difference. This variability may be within-subject, device-specific measurement differences, such as skin hydration, local sweating patterns, strap pressure, or contact stability, rather than inter-individual physiological variation. Note that there may also be local differences in the presence of eccrine sweat glands on the wrist versus nearby areas, which could have contributed to differences in signal quality between devices.

It also highlights the complex interaction between anatomical site, physiological variability, and sensor design, underscoring that placement alone does not determine signal amplitude. Variability was more pronounced in tonic‐level signals, whereas phasic responses (e.g., SCRs) aligned well across devices. This likely reflects differences in skin‐sensor contact or baseline conductance calibration. This variability may reflect within-subject, device-specific measurement factors such as skin hydration, local sweating patterns, strap pressure, electrode size, or sensor contact stability (Boucsein, 2012; Bariya et al., 2020; van Dooren et al., 2012). Differences in signal processing pipelines, such as how each device computes tonic SCL or handles motion artifacts, may also contribute (Benedek and Kaernbach, 2010). Notably, SCL is more influenced by local temperature and sweat gland filling, while SCRs are more tightly linked to transient sympathetic bursts (Boucsein, 2012; Posada‐Quintero et al., 2017). This may explain why tonic‐level differences were more pronounced, whereas phasic SCRs showed better alignment across devices.

Accelerometry (ACC) exhibited modality‐ and axis‐dependent performance. The device-defined dorsoventral axis, *Z*‐axis, showed the strongest agreement (CCC = 0.850), with reliable alignment in time and frequency domains. The *X*‐axis showed moderate agreement (CCC = 0.599), while the *Y*-axis was least consistent (CCC = 0.190). Across all axes, Embrace generally reported lower acceleration magnitudes than E4, possibly due to sensor gain calibration, internal filtering, or device orientation variance. Despite this, coherence remained high in many cases, suggesting that relative motion patterns were well preserved.

Temperature (TEMP) showed moderate structural agreement but notable amplitude differences. EmbracePlus consistently reported lower temperature values (this might also be responsible for the lower SCL values), as confirmed by the negative bias in Bland–Altman plots and vertical shifts in CCC identity plots. Feature correlations were generally moderate to strong for basic statistical measures such as mean, standard deviation, and RMS. However, these did not reach universally high levels, suggesting some variability in signal amplitude or dynamic range across devices. In contrast, entropy‐based features—particularly permutation and SVD entropy, exhibited weaker and less consistent correlations, indicating greater sensitivity to device‐specific signal characteristics or processing differences. While feature‐level variability exists, previous spectral coherence analysis (see in Appendix Figure D29) showed high coherence (> 0.9), supporting strong alignment in the frequency domain. The differences likely stem from calibration discrepancies and differences in thermal insulation or skin contact between the two devices.

### Which physiological signals are most robust to device‐specific variability?

Among the modalities evaluated, BVP emerged as the most robust signal to device‐specific variability. Its time‐domain, frequency‐domain, and nonlinear features were highly correlated across devices. Even minor amplitude differences did not affect the interbeat interval detection or heart rate variability computation.

Phasic EDA features, such as skin conductance responses (SCRs), also showed strong robustness. While tonic signals were more sensitive to device drift and baseline offsets, phasic bursts aligned closely, likely because they represent short‐term sympathetic activations that are less affected by absolute signal scaling.

Accelerometry, particularly along the *Y*‐axis, was more sensitive to hardware orientation, sensor resolution, and filtering differences, making it less robust to device variability. However, high coherence values suggest that relative motion patterns (timing, frequency) are preserved, even if amplitude is not.

Supporting our findings, a notable difference in moderate-to-vigorous physical activity (MVPA) was observed between devices mounted on the same wrist in the paper (Garnett et al., 2022). This supports our own finding that accelerometry, particularly along the *Y*‐axis, is more sensitive to hardware‐specific factors such as device orientation, sensor resolution, and filtering differences. While amplitude measurements varied, high coherence values in our data indicate that relative motion patterns, timing, and frequency structure were still well preserved across devices. This highlights the importance of considering not only the mounting location but also the signal processing pipelines and sensor architecture when comparing accelerometer‐derived metrics.

Temperature signals exhibited moderate agreement in temporal structure, as reflected by correlation-based metrics, but lower agreement in absolute amplitude. Because correlation measures are insensitive to constant amplitude offsets or scaling differences (provided device gain remains stable), these results indicate that both devices capture similar temperature trends over time, even when absolute values differ. This suggests that temperature measurements from these devices are well suited for within-device or trend-based analyses, whereas direct cross-device comparison of absolute temperature values may require additional calibration to account for device-specific offsets or gain differences. Accordingly, calibration strategies such as offset or gain correction could improve absolute agreement without affecting the underlying temporal correspondence.

In summary, EmbracePlus consistently reported lower absolute values across all physiological signals compared to E4, but the relative alignment and structural dynamics were well preserved in most cases. The most reliable agreement was seen in BVP and EDA (phasic), while ACC and TEMP showed greater variability depending on axis or context.

Several factors may account for the systematic differences in absolute signal amplitudes across devices, particularly in EDA, ACC, and TEMP signals. First, the EDA sensors themselves may differ in electrode composition and contact properties. Earlier versions of the E4 employed Ag/AgCl electrodes, while EmbracePlus uses stainless steel (Posada‐Quintero et al., 2017; Empatica S.r.l., 2015; Empatica Support, 2025). Such material differences can alter skin‐electrode impedance and baseline conductance readings. Second, the physical form and materials of the devices differ. The EmbracePlus has a soft, breathable silicone band, whereas the E4’s band is more rigid and less permeable (Empatica, 2021). These factors can influence sensor‐skin contact stability, thermal dissipation, and moisture retention, all of which affect EDA and temperature readings. Third, the placement of the devices on the wrist (EmbracePlus distal, E4 proximal) introduces variability due to known anatomical differences in sweat gland density (van Dooren et al., 2012; Bariya et al., 2020) and vascular structure, which may explain the tonic‐level EDA discrepancies and temperature offsets. Because our synchronization and agreement analyses emphasize temporal alignment, relative signal dynamics, and shape-based similarity, such device-specific amplitude differences are largely orthogonal to the primary objective of assessing cross-device synchronization. Consequently, even when absolute values differ, the preservation of shared temporal patterns supports reliable alignment across platforms. Accordingly, observed amplitude offsets should be interpreted as contextual factors rather than limitations of the synchronization framework.

### Practical usage and recommendations for future users

The proposed pipeline is intended to serve both as a reusable synchronization tool and as a validation framework for assessing cross-device interoperability. In practice, we recommend that users apply the pipeline in two complementary ways. First, when integrating data from different wearable platforms, such as in longitudinal studies, multi-site collaborations, or studies spanning device generations, users should apply the pipeline to a representative subset of concurrent recordings to verify that signals can be reliably synchronized and exhibit sufficient agreement for their intended analyses. This initial validation step enables informed decisions about whether devices can be treated as interchangeable at the signal or feature level.

Second, for use cases closely matching the present study (e.g., wrist-worn research-grade devices, similar recording contexts, and comparable preprocessing choices), users may follow the pipeline directly and reference the present results as evidence that cross-device synchronization and comparison are feasible. Importantly, synchronization should be viewed as a necessary but not sufficient condition for interoperability: agreement metrics at the signal, spectral, and feature levels provide critical guidance on which modalities and features can be meaningfully compared across platforms. More broadly, the pipeline is designed to standardize how cross-device validation is performed rather than to guarantee interchangeability a priori. By making validation explicit and reproducible, the framework allows users to adapt thresholds and decisions to their specific research or clinical objectives.

In line with prior method-comparison literature (Bland & Altman, 1986; Lin, 1989; van Lier et al., 2020), we recommend interpreting multiple metrics jointly rather than relying on a single agreement measure in isolation. For example, high temporal correlation or spectral coherence together with elevated RMSE or systematic Bland–Altman bias may indicate preserved temporal dynamics despite device-specific amplitude scaling or calibration differences. Conversely, strong agreement across concordance, spectral, and feature-level metrics provides stronger evidence that signals or derived biomarkers may be treated as interchangeable for downstream analyses.

## Future research directions, limitations & conclusions

### Future research directions

While this study demonstrates the feasibility of cross‐device signal alignment and comparison, several avenues remain open for future work. The synchronization and analysis pipeline developed for this study, covering ACC, TEMP, BVP, and EDA signals from the EmbracePlus and E4, is already functional for multimodal signal alignment and comparison. Future work could extend this framework to support additional sensors (e.g., SpO_2_, respiration) and wearable platforms. By generalizing the current implementation into an open‐source, modular benchmarking tool, researchers could enable plug‐and‐play compatibility and facilitate scalable cross‐device validation across a wide range of physiological modalities.

Complementing real‐world data with laboratory‐based studies involving standardized stimuli (e.g., cold pressor, Stroop, etc.) would allow for more precise validation of signal agreement under known physiological activations. These settings could help disentangle signal discrepancies due to device variability versus behavioral or environmental noise.

## Limitations

Despite its contributions, this study has several limitations:

Firstly, the data were collected in real-world conditions without contextual or event‐level annotations, limiting the ability to attribute signal patterns to specific behaviors, emotional states, or environmental factors.

Moreover, although we worked with what Empatica labels as “raw” sensor streams, we do not have full visibility into the firmware‐level filtering, resampling, the downstream cloud-based preprocessing or quality‐control steps that shape those data before they are written to cloud storage. For example, EmbracePlus runs an on‐wrist detection routine that flags the device as “not worn” after five minutes and automatically censors the corresponding data segments, a feature that is undocumented for the legacy E4 (Empatica Inc, 2025a). Moreover, the signals we downloaded arrived in Avro files produced by Empatica’s cloud pipeline, whose exact preprocessing details (e.g., digital filtering, artifact rejection) are not publicly disclosed (Empatica Inc, 2025b). Such proprietary, “black‐box” processing has been widely noted as a general limitation when validating research‐grade wearables, because firmware updates or hidden algorithms can introduce unnoticed drifts or biases in the measured parameters (Jacobsen et al., 2020). These factors must be considered when interpreting the apparent agreement between EmbracePlus and E4, and they highlight the need for future studies to track firmware versions and, where possible, to access unprocessed sensor outputs.

The participant cohort represents a narrow demographic or clinical group. The participant cohort in this study is relatively homogeneous, comprising 31 individuals (20 women and 11 men) recruited from hospital research staff at the Hospital Clínic of Barcelona, Spain. Given that participants were also self-identified as Caucasian, the sample represents a narrow demographic. Consequently, the robustness of the proposed synchronization and agreement analyses under more diverse physiological, behavioral, or environmental conditions—such as those associated with different age groups, ethnic backgrounds, health statuses, or activity patterns—remains to be established. Future studies should therefore evaluate synchronization performance across more heterogeneous populations to assess generalizability under a broader range of input signal characteristics.

While the Empatica E4 device is no longer commercially available, the relevance of publishing this research remains high for several important reasons:

- The Empatica E4 was widely used in earlier studies, making it an important benchmark for physiological monitoring in wearable research. Many existing studies have utilized the E4 to collect data on BVP, EDA, ACC, and TEMP, creating a significant body of research that relies on its data. This study provides valuable insights into how the EmbracePlus (a newer device) compares with the E4, helping maintain continuity and consistency in the literature.
- Future researchers aiming to compare new devices with the Empatica E4 for data replication or comparison purposes will find this study beneficial, as it provides a clear understanding of the differences in measurements and synchronization between the devices.
- As the Empatica E4 has been discontinued, many research groups are transitioning to the EmbracePlus or other upgraded devices. However, this transition poses a challenge for data compatibility and device synchronization. Many researchers are unaware of how well the new devices align with the E4's collected data. This study bridges that gap by providing a detailed analysis of how EmbracePlus and E4 perform together in terms of signal synchronization and data agreement, which is critical for researchers continuing studies involving the E4.

This study was not designed around a priori power analysis. The sample size (*N* = 31) was determined by feasibility and is consistent with prior wearable validation studies. As this work aims to demonstrate a generalizable synchronization pipeline, future applications may incorporate power analyses tailored to specific signals, their derived biomarkers, and agreement metrics.

Addressing these limitations through standardized protocols, expanded populations, and device‐agnostic pipelines would support more robust cross‐platform analyses in the growing field of wearable physiology.

## Conclusion

This study presents a standardized, end-to-end framework for synchronizing and validating physiological signals acquired concurrently from different wearable platforms. By integrating temporal alignment with signal-level, spectral, and feature-level agreement analyses, the proposed pipeline extends prior synchronization-focused approaches and provides a practical methodology for assessing cross-device interoperability across multiple physiological modalities. Applied to concurrent recordings from the Empatica E4 and EmbracePlus, the framework demonstrated that several signals, particularly BVP and phasic EDA, can be reliably synchronized and compared, while also highlighting modality-specific limitations and sources of variability.

Beyond the specific devices examined here, the proposed approach is intended as a reusable validation tool for researchers and clinicians facing the increasingly common challenge of integrating physiological data across platforms, device generations, or study sites. By lowering technical barriers and making cross-device validation explicit and reproducible, this work supports more robust, transparent, and equitable use of wearable physiological data in both research and applied settings.

## Supplementary Information

Below is the link to the electronic supplementary material.

Supplementary file1 (DOCX 12146 KB)

Supplementary file2 (DOCX 4942 KB)

## Data Availability

The datasets generated and/or analyzed during the current study are available in the University Clinic Barcelona Data Repository and have been mirrored on the Open Science Framework (OSF) to facilitate peer review. The data are provided as a set of compressed.zip files, which together constitute the original dataset folder titled “E4xEmbrace.” When unzipped, the folder contains the raw physiological recordings from the Empatica E4 and EmbracePlus devices used in this study. Due to privacy and ethical considerations, the datasets are shared in de-identified form. Researchers may request additional access through the institutional repository’s request system (https://forms.office.com/e/eW33QQEvh3). The data for peer review are accessible via the OSF link: https://osf.io/qmhr2/overview?view_only=f86c5a8f7ff3406abfbc912c0f3c32d9 Upon publication, the dataset will be made openly accessible in accordance with institutional and ethical guidelines.

## Abbreviations

*EDA*: Electro‐dermal activity
*BVP*: Blood volume pulse
*HRV*: Heart rate variability
*ACC*: Accelerometer
*TEMP*: Temperature

## Code availability

Code used in this work is attached as supplementary material, and the code will be made publicly available on GitHub at INTREPIBD/e-plus-integration upon publication.
