# Supplementary material for: Standardized synchronization and validation pipeline for physiological biomarkers across multiple devices
Source: Behav Res Methods. 2026 Jul 16;58(8):239. doi: 10.3758/s13428-026-03097-8 (PMC13375695; doi:10.3758/s13428-026-03097-8)
Supplement: Supplementary file 1 — Supplementary file1 (DOCX 12146 KB) [file 13428_2026_3097_MOESM1_ESM.docx]

**APPENDIX**

1. **BLOOD VOLUME PULSE ANALYSIS**


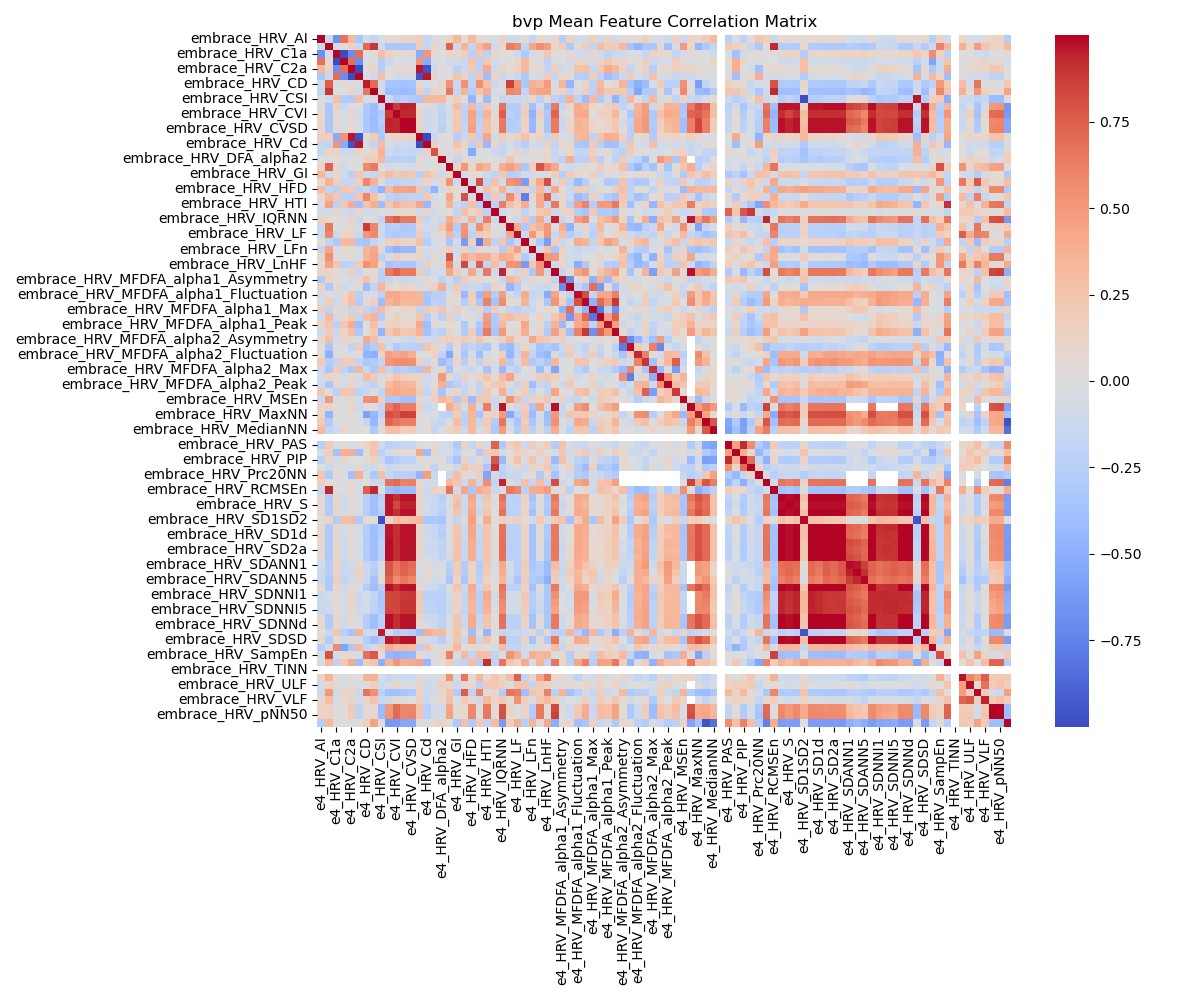
The BVP signal exhibited near‐perfect agreement between devices. As shown in Figure A1, the feature correlation matrix revealed highly consistent BVP‐derived metrics. Bland–Altman analysis confirmed minimal bias (0.036) with narrow limits of agreement, while RMSE and MAE values were extremely low (0.037 and 0.031). The concordance correlation coefficient (CCC) was 1.000, indicating exact linear and scale agreement. Furthermore, the mutual information (MI) and normalized MI (NMI) scores of 2.995 and 0.928, respectively, affirmed shared nonlinear structure. High phase locking value (PLV = 0.998) and coherence (1.000) underscore the robust synchrony across time and frequency domains.

**F I G U R E A1 BVP Feature Correlation Matrix.** This heatmap displays mean Pearson correlation coefficients between BVP‐derived features from the Embrace Plus and Empatica E4 devices, computed across all 31 participants. The high‐intensity red diagonal and consistent off‐diagonal structure indicate strong agreement across multiple heart rate variability metrics.

Key observations from the Figure A1 include:

- **Near‐Perfect Diagonal Correlations**: Most matching features (e.g., HRV_SDNN, HRV_MeanNN, HRV_HF, HRV_LF) show correlation coefficients > 0.9, suggesting excellent agreement in feature computation across devices. This validates both the preprocessing pipeline and the alignment accuracy of the BVP signals.
- **High Consistency in Time‐Domain HRV Features**: Features such as HRV_SDNN, HRV_SDSD, and HRV_pNN50 show particularly high correlations, indicating that temporal heart rate variability metrics are robust to device‐specific signal differences. These features are typically computed from interbeat intervals derived via peak detection.
- **Frequency‐Domain Features Also Well Aligned:** Components such as HRV_LF, HRV_HF, HRV_VLF, and HRV_ULF show strong correlations (*r* > 0.85), implying that the spectral power distribution of the derived HRV signals is preserved across devices. This is crucial for applications involving autonomic nervous system (ANS) activity estimation.
- **Nonlinear and Complexity Features Slightly More Variable:** Features like HRV_SampEn, HRV_DFA*, HRV_MFDFA_alpha*, and HRV_MSEn show moderate to high correlations (ranging between 0.7 and 0.9), but with slightly more variability across participants. These features are sensitive to noise and require high‐quality, clean inter beat intervals, any minor inconsistency in peak detection or segmentation may impact their computation.
- **No Spurious Cross‐Correlations:** Off‐diagonal correlations between unrelated features are low, suggesting no major leakage or misalignment in feature mapping across devices.


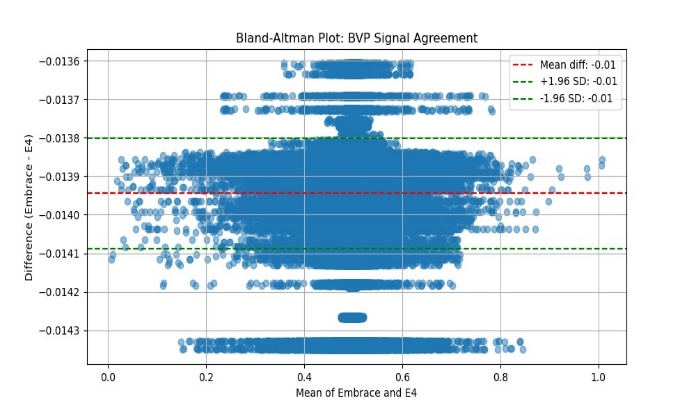

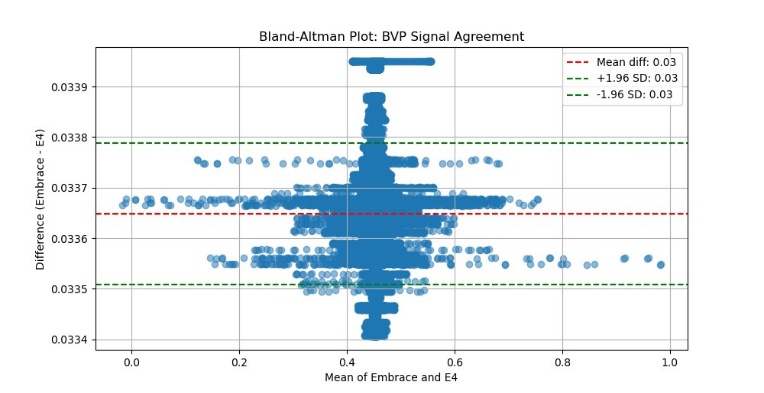


1. Participant 2
2. Participant 3

**F I G U R E A2 Bland–Altman Plots Comparing BVP Signal Agreement Between Embrace Plus and E4 for Two Participants.**

These plots display the signal mean (x‐axis) versus the difference (Embrace − E4, y‐axis) for each data point. For Participant 2, the bias is slightly negative and tightly centered, indicating excellent agreement. Participant 3 shows a slight positive bias, but the limits of agreement remain narrow, indicating reliable performance across subjects. Even minimal differences in sensor placement (proximal vs. distal positioning) produce measurable PPG waveform delays due to pulse transit time (PTT), as the arterial pulse travels along the vessel (Deshmukh et al., 2022; Li et al., 2018). Furthermore, PPG morphology and amplitude can vary by measurement site, e.g. wrist versus finger or ear—reflecting local vascular properties and signal attenuation (Wang et al., 2023; Kusche et al., 2020). Additionally, applied pressure from the wristband affects waveform characteristics, potentially introducing slight timing and shape distortions . While the proximal‐to‐distal placement differences between EmbracePlus and E4 in our study were small, they are consistent with known physiologic delays and morphological variations seen in PPG signals.

Figure A2 shows Bland–Altman plots for two representative participants (Participant 2 and Participant 3), illustrating the level of agreement between BVP signals recorded by the Embrace Plus and Empatica E4 devices on an individual level. These examples are consistent with trends observed across the full cohort of 30 participants and support several key conclusions:

- **Consistently Narrow Limits of Agreement Across Participants:** Across the entire participant pool, the limits of agreement between the Embrace and E4 BVP signals remain narrow (±1.96 SD) , typically within a small fraction of the normalized signal range. This indicates high consistency in the absolute BVP values measured by the two devices. The tight spread (limits span an extremely small amplitude range (e.g., on the order of 0.0005 to 0.001 in normalized units), demonstrating minimal variability in signal acquisition and preprocessing outcomes) suggests that, for most individuals, device differences are minor and not physiologically significant.
- **Small, Centered Bias in Most Cases:** Most participants exhibit minimal mean differences (bias) between devices, often close to zero. In the provided examples:
  - Participant 2 shows a slight negative bias, while
  - Participant 3 exhibits a mild positive bias.

**This variability in bias direction is consistent across the dataset**, with no systematic over‐ or underestimation trend attributable to either device. This suggests that the alignment and preprocessing pipeline successfully mitigates systemic drift or offset between sources.

- **Lack of Proportional Bias:** Across participants, the differences between signals remain stable over the range of signal intensities. This absence of proportional bias implies that agreement is not dependent on the magnitude of the BVP signal, further validating the robustness of both devices for use across various physiological conditions.
- **Individual Differences Remain Within Acceptable Ranges:** The limits of agreement derived from Bland–Altman analysis indicate measurement precision but do not inherently provide clinically meaningful thresholds for acceptability. Instead, thresholds such as the widely‐used ≈ 30% percentage error (Critchley et al., 1999; Odor, 2017) provide established clinical benchmarks to evaluate whether measurement agreement is sufficient for practical use. To further quantify measurement agreement, we computed the percentage error based on Bland–Altman analysis. This metric is commonly used in clinical validation studies (Critchley et al., 1999) and is calculated as:

$Percentage Error \left( \% \right)=\frac{1.96 x {SD}_{diff}}{Mean of Means}X100$ , where SD_diff_ is the standard deviation of the differences between devices, and the Mean of Means refers to the average of the two device signals. This gives a normalized estimate of agreement precision. For the participants in this study, the limits of agreement were extremely narrow (on the order of ±0.0015), and the average signal amplitude was approximately 0.5 (normalized units). This yields a percentage error of approximately 0.6% in all cases. Given that clinical acceptability thresholds often allow up to ±30% error for device agreement (Critchley et al., 1999), these results indicate exceptionally high agreement and precision between the EmbracePlus and E4 devices. Such variability can be attributed to factors like skin tone, sensor placement, or transient motion artifacts, but they do not undermine the overall reliability of the signal alignment process.


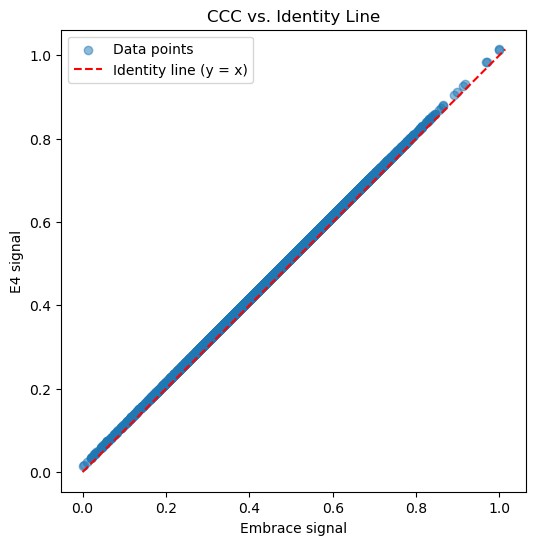

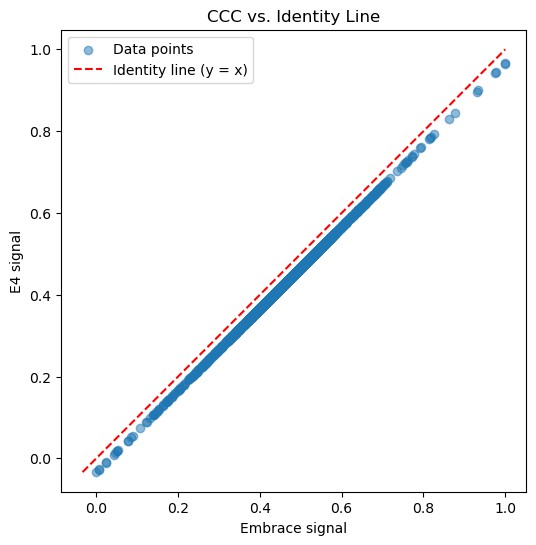


1. Participant 2
2. Participant 3

**F I G U R E A3 Concordance Correlation Coefficient (CCC) Identity Plots for BVP Signal.** These plots show the relationship between Embrace and E4 BVP signals across participants. Each point represents a time‐aligned sample. The proximity of the points to the red identity line (*y* _=_ *x*) confirms strong agreement in both magnitude and scale. Participant 2 shows near‐perfect concordance, while Participant 3 still exhibits high linear alignment with minimal deviation.

To complement the Bland–Altman agreement evaluation, we further examined the alignment between BVP signals using both Concordance Correlation Coefficient (CCC) identity plots and spectral coherence analysis on an individual level. Figure A3 presents two representative CCC identity plots for participants with differing degrees of agreement. Participant 2 (left) exhibits an almost perfect alignment of data points along the identity line y=x, signifying extremely high concordance between Embrace Plus and Empatica E4 BVP signals. The tight clustering with minimal dispersion around the diagonal reflects near‐identical temporal structure in the captured waveforms, underscoring the effectiveness of our alignment and preprocessing pipelines. In contrast, Participant 3 (right) still demonstrates strong overall concordance but with slightly more dispersion, particularly at signal extremes. This pattern, seen over the whole cohort, suggests minor amplitude discrepancies or lag in peak correspondence, likely due to variations in contact quality or physiological noise.


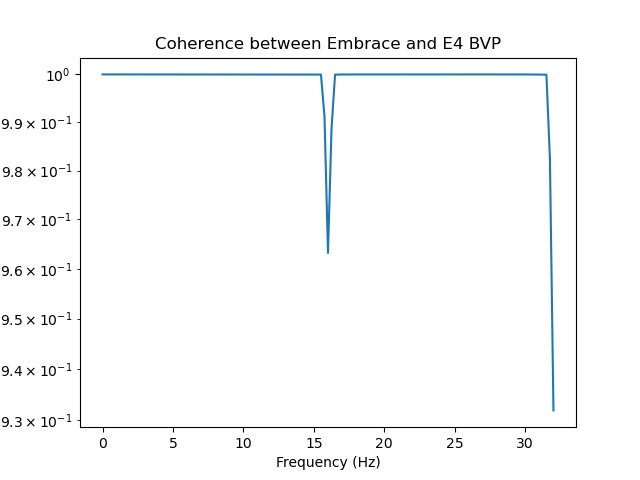

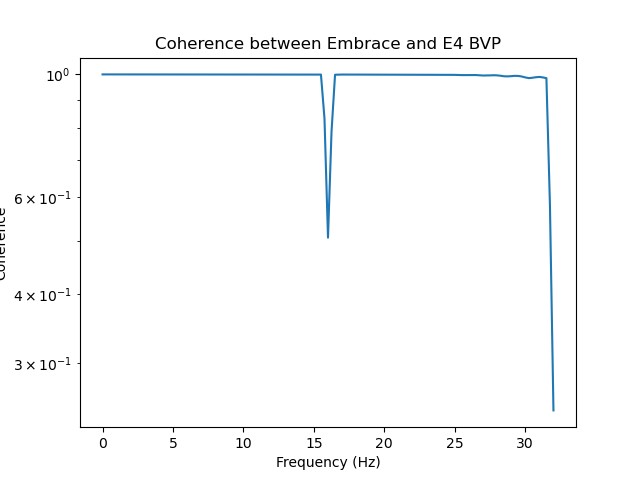


(a) Participant 2

1. Participant 3

**F I G U R E A4 Spectral Coherence Between Embrace and E4 BVP Signals.** These plots represent the frequency‐domain coherence across participants. Participant 2 shows nearly flat coherence close to 1.0 across the spectrum, with a minor dip likely due to sampling noise. Participant 3 follows a similar trend, reaffirming strong phase synchrony and frequency alignment between the devices.

In parallel, Figure A4 displays spectral coherence plots for the same participants, quantifying signal similarity across frequency bands. Participant 2 shows uniformly high coherence (>0.98) across the 0–32 Hz spectrum, confirming a strong frequency‐wise match in both low frequency and high‐frequency components. Participant 3 maintains high coherence above 0.95 across most frequencies but displays a slight dip near 30Hz and at a notch near 16Hz—frequencies where either hardware filtering or motion‐related artifacts may affect the signal. Notably, the persistent coherence plateau across the physiological band (0.5– 5Hz) ensures reliability for heart rate and variability analysis in both devices. Most participants (27 out of 31, 87.1%) exhibit CCC values above 0.95 and coherence consistently above 0.97 in the 1–5Hz band, validating the temporal and spectral integrity of our cross‐device BVP alignment.


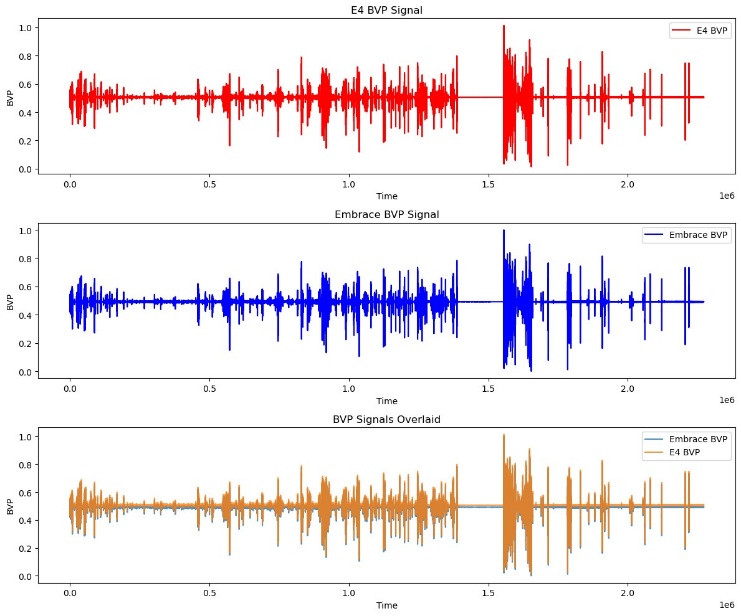

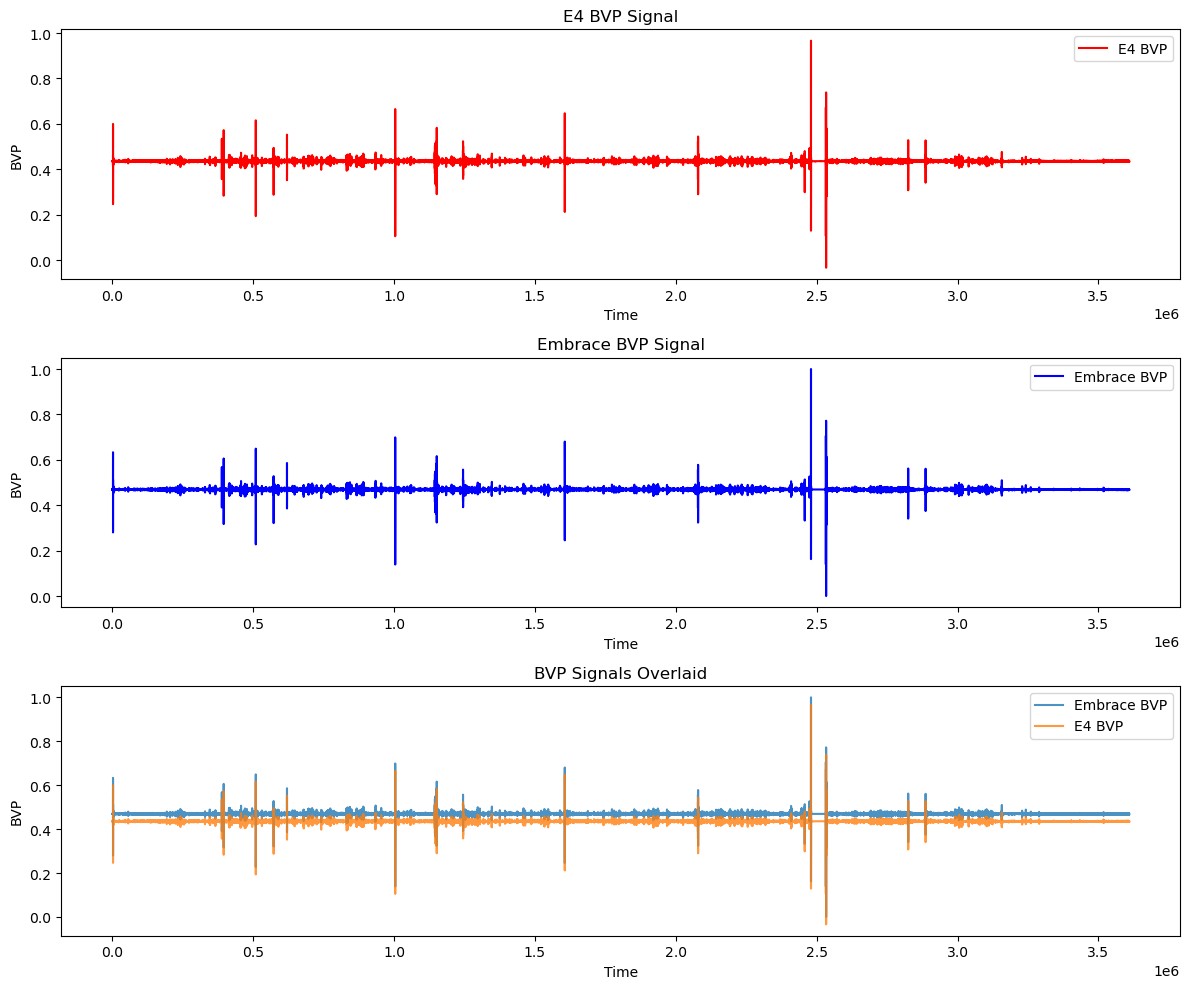


1. Participant 2
2. Participant 3

**F I G U R E A5 Visual Comparison of Raw and Aligned BVP Signals.** Each panel includes three subplots: the E4 BVP signal, the Embrace BVP signal, and their overlay. For both participants, signals are well‐aligned temporally and match closely in amplitude and shape. The overlay highlights minimal lag and strong structural similarity, demonstrating the effectiveness of our synchronization and preprocessing pipeline.

Figure A5 shows raw signal overlays with minimal visual divergence, confirming the robustness of alignment.

**B ELECTRODERMAL ACTIVITY ANALYSIS**

EDA signal alignment also yielded strong performance, albeit with modest variability. As visualized in Figure B6, several features demonstrated moderate‐to‐high correlation. The alignment metrics—RMSE = 0.077, MAE = 0.064, CCC = 0.776—indicate good agreement, supported by strong MI (1.793) and NMI (0.780). KL divergence (2.594) suggests moderate distributional differences. Despite this, the high Pearson correlation (0.777), PLV (0.985), and spectral coherence (0.844) suggest temporal and spectral stability across devices.


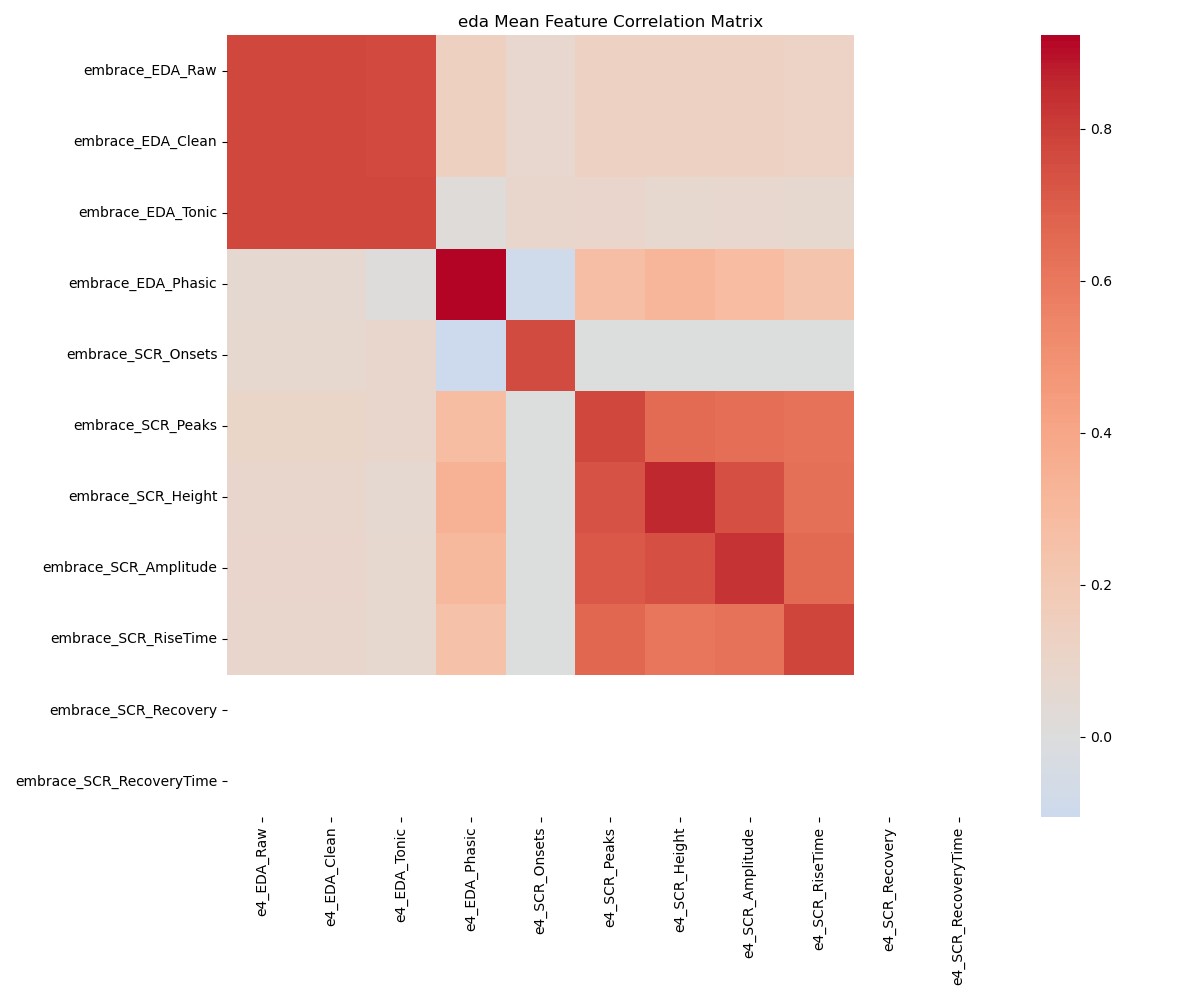


**F I G U R E B6 EDA Feature Correlation Matrix.** The heatmap visualizes cross‐device correlations for EDA‐related features. Most features exhibit moderate to strong positive correlation, particularly for phasic responses and amplitude‐related metrics. Some variability is observed in tonic‐level correlations, which may reflect physiological or device specific differences. These results align with the findings in Section 3.2.

Key observations from the heatmap include:

- **Moderate to Strong Cross‐Correlation in Tonic/Phasic Decompositions:** the heatmap shows that Embrace’s tonic component (embrace_EDA_Tonic) exhibits moderate to strong correlation _(_*r* ≈ 0.6–0.7) with E4’s tonic component (e4_EDA_Tonic). This suggests a degree of consistency in baseline skin conductance level between the devices. Phasic components (embrace_EDA_Phasic) show the strongest correlation with their E4 counterparts—especially with e4_EDA_Phasic (*r* > 0.85), confirming that short term EDA fluctuations (typically driven by sympathetic activity) are well aligned across both platforms. These results imply that the decomposition

into tonic and phasic is largely preserved across devices, which is a positive validation of both signal quality and preprocessing consistency.

- **High Within‐Modality SCR Agreement:** Features such as SCR_Height, SCR_Amplitude, and SCR_Peaks show strong cross device correlations (*r* > 0.7), especially in pairwise comparisons. These features are inherently more event‐based and less sensitive to tonic drift, leading to more reproducible measurements. Notably, SCR_Peaks and SCR_Amplitude show higher alignment than SCR_Onsets or Rise_Time, possibly because peak‐based features are more robust to subtle onset timing differences.
- **Minimal Cross‐Talk or Spurious Correlation:** Off‐diagonal blocks show generally low correlation values, indicating that unrelated features across modalities (e.g., Embrace SCR_Peaks vs E4_Tonic) are not spuriously correlated. This supports the integrity of feature extraction and mapping between devices.
- **Percentage Error:** We applied the same percentage error estimation method to the electrodermal activity (EDA) signals to assess the consistency of measurements between the EmbracePlus and E4 devices. The average percentage error was approximately 26.0%. This falls within the commonly accepted clinical threshold of ±30% for measurement agreement (Critchley et al., 1999), suggesting reasonable agreement between the two devices for EDA measurements, despite greater variability compared to BVP signals. These results highlight that while EDA signal alignment is generally reliable, it may be more sensitive to individual‐specific factors such as skin conductance properties or sensor placement.


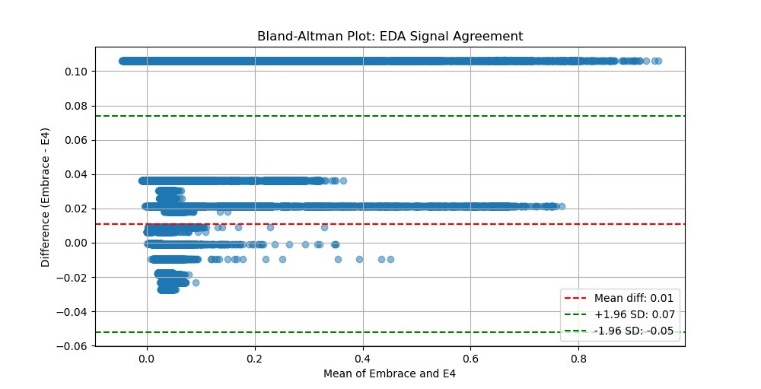

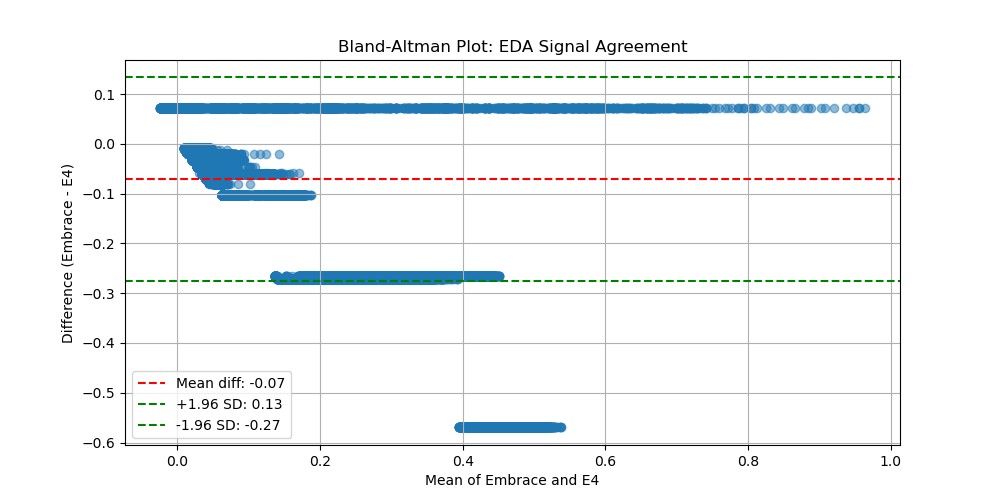


1. Participant 2
2. Participant 3

**F I G U R E B7 Bland–Altman Plots for EDA Signal Agreement.** These plots show the differences between the Embrace and E4 EDA signals versus their mean values. **Participant 2** exhibits minimal bias and narrow limits of agreement (LoA), indicating strong consistency in EDA signals across devices. **Participant 3**, in contrast, shows a more pronounced negative bias and wider LoA, suggesting increased variability and possible physiological or placement‐related discrepancies.

To complement the aggregate results, we examined individual‐level trends in Bland–Altman agreement for EDA signals, similar to the BVP analysis. Figure B7 presents two representative participants to illustrate the variability in signal agreement across individuals. For Participant 2, the EDA signals show very tight agreement with a negligible mean bias of approximately 0.01. The limits of agreement are also narrow _(+_ – 0._06)_, suggesting consistent EDA capture and minimal device‐specific drift. In contrast, Participant 3 demonstrates a noticeable negative bias (*mean_diff* ≈ –0.07) and a wider spread in the limits of agreement (*from* – 0.27*to* + 0.13). This indicates some degree of divergence between devices, potentially caused by localized signal noise or varying skin‐electrode interactions.

While variability exists, the majority of data points for both participants lie well within the 95% limits of agreement. This suggests that, despite small systematic offsets, both devices generally provide comparable EDA trends across time. The vertical clustering of points in Participant 3’s plot indicates regions where one device’s signal remains consistently higher or lower. This could reflect differing sensor sensitivities, differences in sensor placement as mentioned earlier, or baseline shifts introduced by calibration differences or perspiration dynamics. There is no discernible upward or downward trend across the x‐axis (mean of devices), indicating that signal differences do not vary systematically with signal intensity. In other words, disagreement does not worsen at higher or lower EDA values.


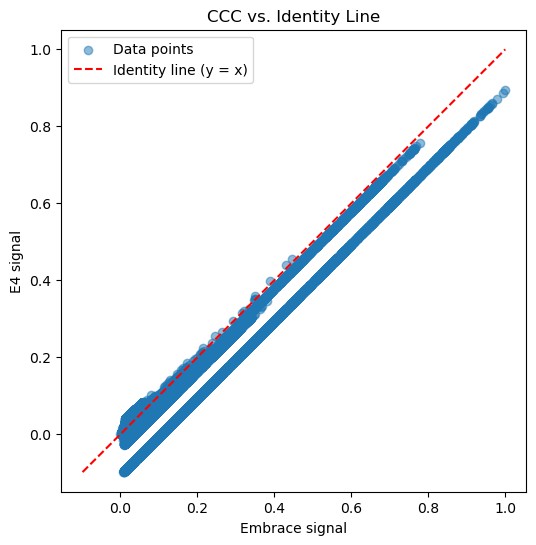

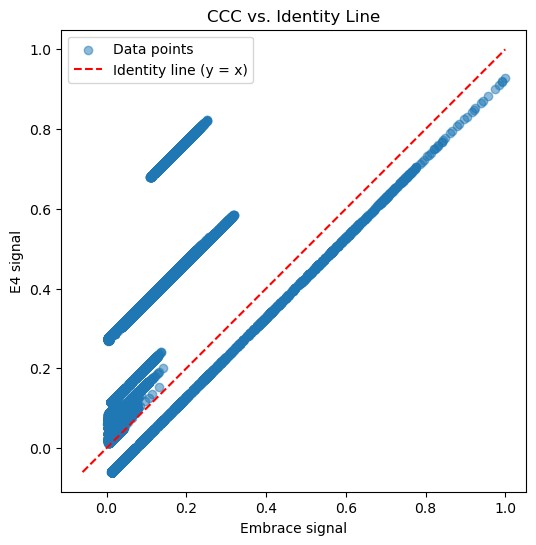


1. Participant 2
2. Participant 3

**F I G U R E B8 Concordance Correlation Coefficient (CCC) Identity Plots for EDA Signal.** These scatter plots compare the EDA signal values from Embrace and E4 devices. Participant 2 shows stronger alignment along the identity line (*y* _=_ *x*) compared to Participant 3, who shows slight clustering deviations, suggesting reduced but still moderate concordance.

To further validate cross‐device consistency at the raw signal level, we analyzed EDA signals from the Embrace Plus and Empatica E4 for concordance correlation. The CCC scatter plots from the Figure B8 compare synchronized EDA values from Embrace and E4 on a per‐sample basis for two participants. Participant 2 shows near‐perfect alignment with the identity line *y* _=_ *x*, indicating excellent one‐to‐one correspondence in EDA magnitude across the two devices. This reflects strong linear agreement and highly consistent amplitude scaling. Participant 3 also displays generally high agreement, though with more pronounced deviations from the identity line—particularly at low and mid‐range EDA values. These patterns suggest a structured offset that may reflect minor differences in baseline calibration, device‐specific gain, or signal normalization. CCC values across participants typically range from 0.85 to 0.99, reflecting strong overall concordance. These deviations, while small in magnitude, appear systematic rather than random, and may become more salient in low‐conductance ranges due to hardware sensitivity differences or sensor placement. These results support the effectiveness of the alignment procedure and indicate minimal systemic bias across device pairs.


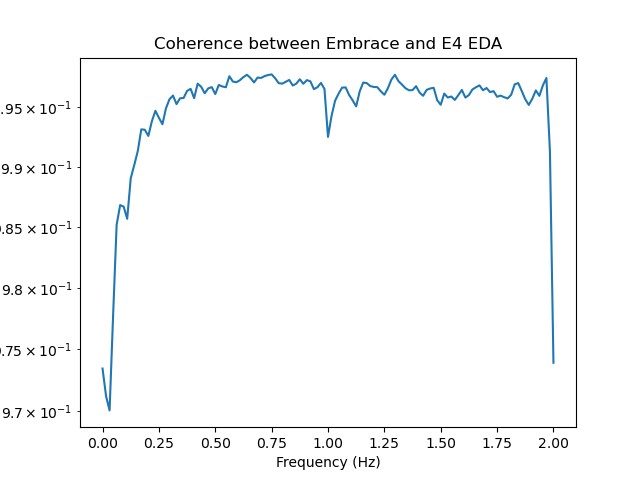

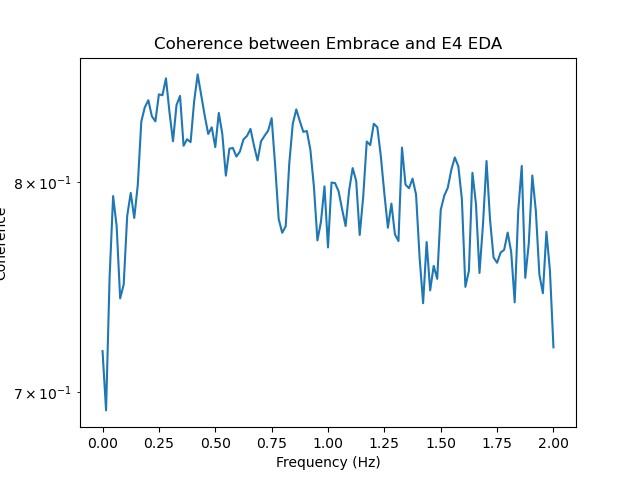


1. Participant 2
2. Participant 3

**F I G U R E B9 Spectral Coherence Between Embrace and E4 EDA Signals.** Coherence plots show frequency‐wise similarity between the devices. Participant 2 demonstrates consistently higher coherence above 0.95, while Participant 3 exhibits slightly lower consistency, especially in lower frequencies.

Magnitude‐squared coherence plots assess the similarity in frequency content between the synchronized EDA signals from both devices. Participant 2 exhibits coherence values above 0.95 across the full 0–2 Hz band, indicating nearly identical temporal structure and frequency content. This suggests both devices are capturing the same dynamic EDA fluctuations, including both tonic and phasic responses. Participant 3 shows relatively lower coherence, fluctuating between 0.7 and 0.9, particularly in the higher‐frequency range. This may reflect increased motion artifacts, variable sensor‐skin contact, or differences in device‐specific smoothing.

For most users (25 out of 31), coherence remains above 0.90 in the dominant EDA frequency band (<1 Hz), supporting strong spectral agreement. Notable dips in coherence at higher frequencies may indicate minor timing jitter, aliasing, or variability in sampling consistency. These findings suggest the devices are broadly equivalent in capturing the autonomic nervous system’s phasic response spectrum.


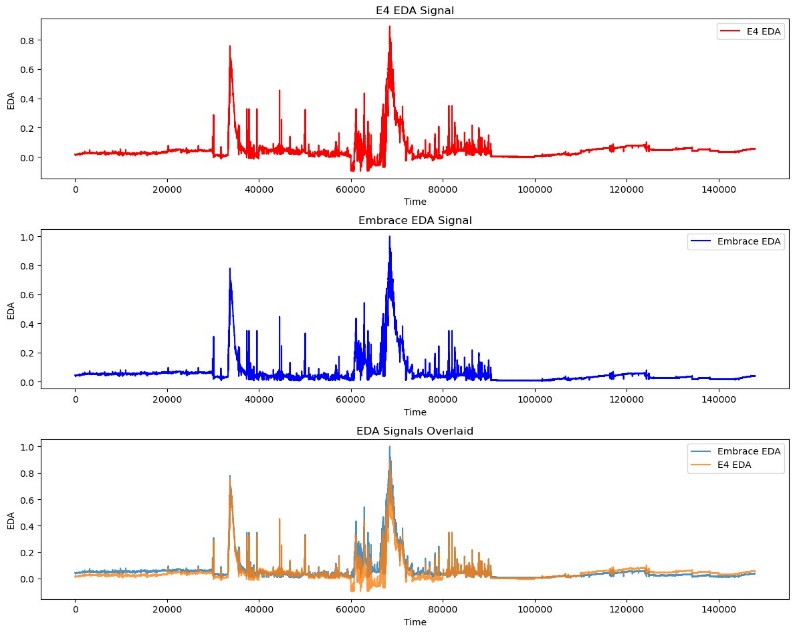

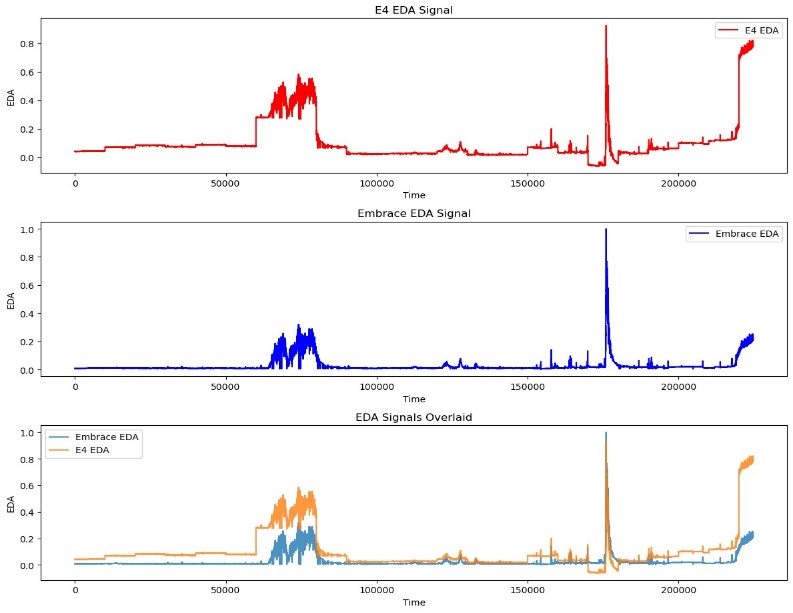


1. Participant 2
2. Participant 3

**F I G U R E B10 Raw and Aligned EDA Signals from Embrace and E4 Devices.** Both panels show EDA signals for Embrace and E4 separately and overlaid. Participant 2 demonstrates more consistent alignment in both timing and amplitude, while Participant 3 shows some signal divergence in key peaks, highlighting inter‐subject variation.

Side‐by‐side plots of raw and normalized EDA signals from both devices reveal visual agreement over long recording windows. Similar plots appear in Picard et al. (Picard et al., 2015). For Participant 2, the raw EDA traces are highly synchronized, with overlapping onset and amplitude of stimulus‐driven peaks, confirming alignment precision. Both devices track long‐term tonic trends and short‐lived phasic bursts similarly. In Participant 3, signals also show aligned peaks, but diverge more during periods of low activity or baseline. Embrace appears slightly smoother, potentially due to internal filtering or sampling granularity. Even when worn on adjacent positions on the same wrist, small spatial differences can introduce measurable variability in skin conductance, as demonstrated in multi‐site EDA comparisons (van Dooren et al., 2012).

**C ACCELEROMETER ANALYSIS**

Accelerometer performance varied considerably by axis (see Figures C11, C16.C21).

X‐axis: Moderate agreement was observed (CCC = 0.599, RMSE = 0.097, Pearson r = 0.355). Coherence was notably high (0.975), indicating stable spectral alignment despite amplitude variability.


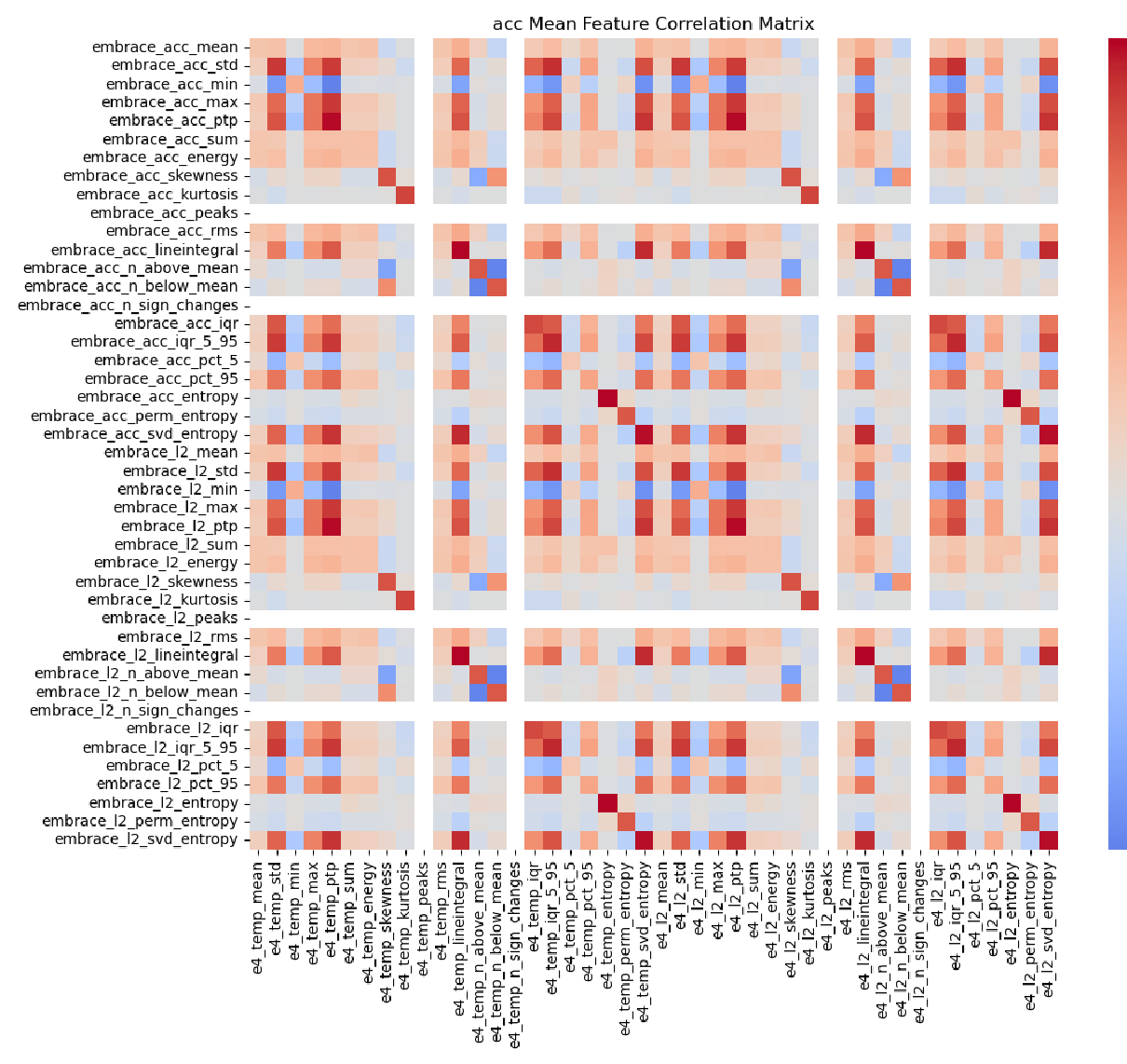


**F I G U R E C11 Accelerometer X‐Axis Feature Correlation Matrix.** Correlations between X‐axis accelerometry features show moderate consistency, with a few clusters of agreement. The matrix highlights moderate correlation for energy, RMS, and entropy‐based features, though linearity and higher‐order statistical features appear less aligned. These trends reflect the results discussed in Section 3.3.

The feature correlation matrix for ACC‐X (Figure shown above) reveals:

- **Moderate feature agreement**: Several statistical descriptors (e.g., mean, std, energy, rms, ptp) show moderate correlations between devices — typically in the r=0.4 to 0.7 range.
- **Complexity features (entropy variants, sign changes, peaks) exhibit more variability**, indicating that minor differences in signal dynamics and resolution may affect higher‐order feature consistency.
- **Cross‐feature misalignment is minimal, with higher correlation concentrated along the diagonal (same feature types)**, suggesting good mapping fidelity between signal‐derived features from each device.


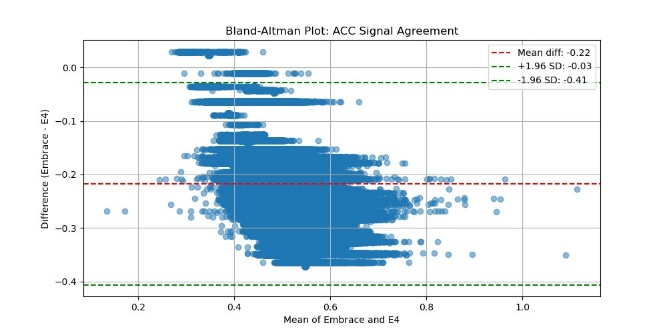

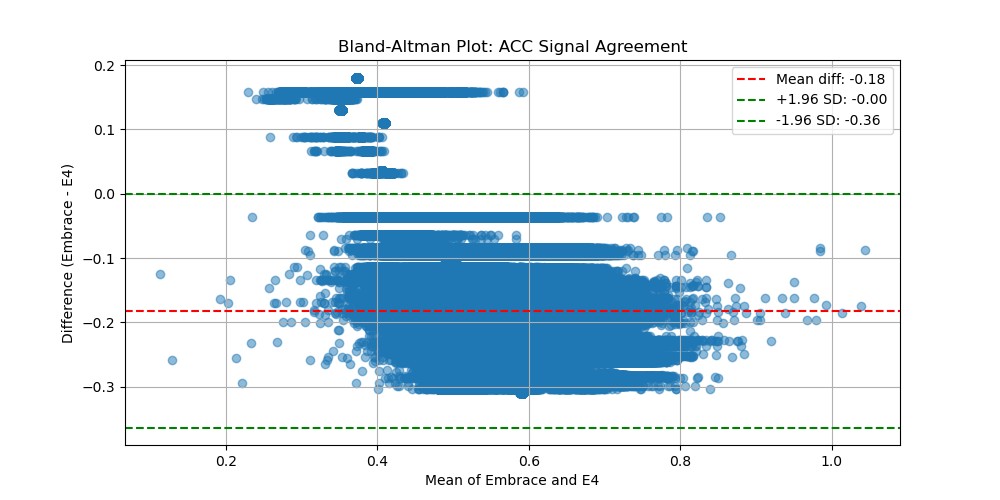


1. Participant 2
2. Participant 3

**F I G U R E C12 Bland–Altman Plots for ACC‐x Signal Agreement.** The plots show the agreement between Embrace and E4 accelerometer (x‐axis) signals. Participant 2 exhibits a noticeable negative bias and a broader spread, indicating reduced signal agreement. Participant 3 shows a slightly smaller bias, though variability remains wide. This may reflect differences in how the devices respond to high‐frequency motion or short‐term movement mismatches, potentially due to brief periods of loose contact, inconsistent placement, or device handling. While accelerometer signals reflect actual movement, discrepancies between devices may sometimes reflect sensor‐specific sensitivity or mechanical artifacts rather than true differences in physical activity.

The plots show the agreement between Embrace and E4 accelerometer (x‐axis) signals. Participant 2 exhibits a noticeable negative bias and a broader spread, indicating reduced signal agreement. Participant 3 shows a slightly smaller bias, though variability remains wide. This may reflect differences in how the devices respond to high‐frequency motion or short‐term movement mismatches, potentially due to brief periods of loose contact, inconsistent placement, or device handling. While accelerometer signals reflect actual movement, discrepancies between devices may sometimes reflect sensor‐specific sensitivity or mechanical artifacts rather than true differences in physical activity.

Two representative participants’ Bland–Altman plots show:

- **Consistent negative bias:** Both plots (mean differences: ‐0.22 and ‐0.18) suggest the Embrace Plus tends to report slightly lower values than the E4 for ACC‐X.
- **Narrow limits of agreement:** The spread remains tight, particularly near the mean activity levels, indicating stable relative differences and low random variability.
- **Trend Stability:** No obvious heteroscedasticity (variance increasing with amplitude), suggesting uniform bias across intensity levels.
- While absolute amplitude differs (systematic offset), **the relative shape and dynamics of the signals are preserved**.
- We assessed the agreement between accelerometer signals, x‐axis, recorded by EmbracePlus and E4 using percentage error derived from Bland–Altman limits of agreement. Following the same formula used for other modalities. The resulting average percentage errors were approximately 40.0%. The X axis exceeds the acceptable threshold of 30.0%, suggesting greater measurement divergence across devices. These results may reflect differences in sensor orientation, placement, or device‐specific preprocessing pipelines for motion data.


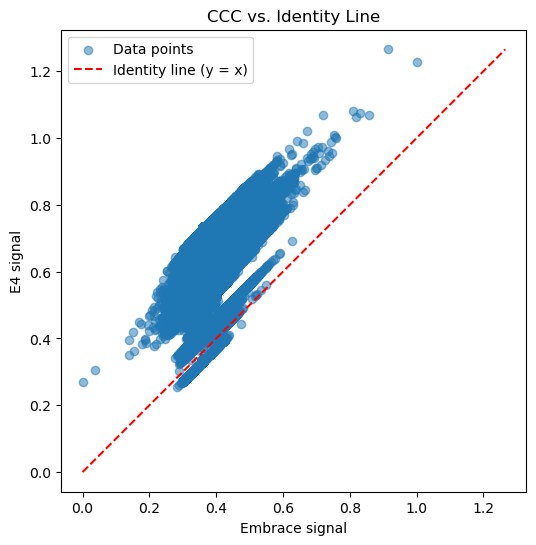

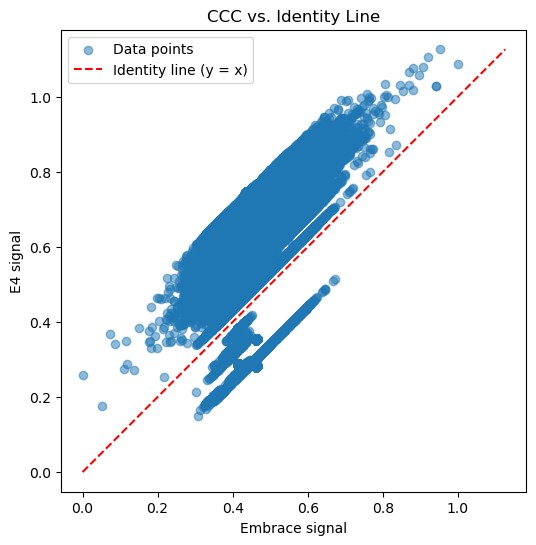


1. Participant 2
2. Participant 3

**F I G U R E C13 Concordance Correlation Coefficient (CCC) Identity Plots for ACC‐x Signal.** Participant 2 shows clustered points deviating from the identity line, indicating lower concordance. Participant 3 demonstrates tighter grouping along the identity line, reflecting improved signal similarity in dynamic activity patterns.

We can make the following observations from the representative samples:

- Participant 2 shows high CCC with data points tightly clustered along the identity line (y=x), indicating strong agreement.
- Participant 3 exhibits slightly larger deviations, but overall follows the same trend.
- **Across both plots, Embrace values trend slightly lower**, reflected by the alignment of the identity line above the data cloud.

The CCC of 0.599 is a reflection of this balance between structural similarity and amplitude divergence.


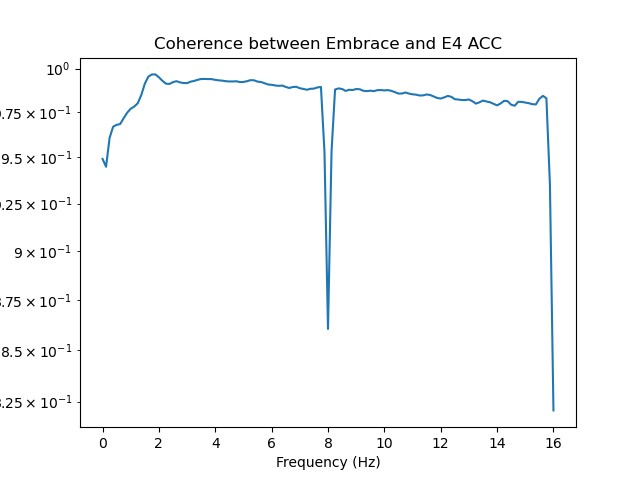

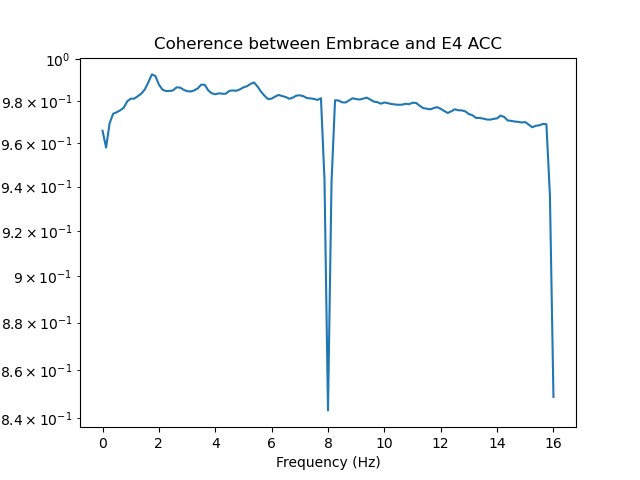


1. Participant 2
2. Participant 3

**F I G U R E C14 Spectral Coherence Between Embrace and E4 ACC‐x Signals.** The coherence spectra highlight frequency‐dependent agreement. Participant 2 maintains coherence around 0.95 across most frequencies, while Participant 3 reveals dips in certain bands, suggesting lower frequency synchronization possibly due to inconsistent sensor alignment.

Coherence remains above 0.97 across most of the spectrum, except for a minor dip near 8 Hz, likely related to a notch filter or aliasing artifact. **High coherence indicates both devices capture the same temporal‐frequency characteristics of movement despite amplitude differences.**


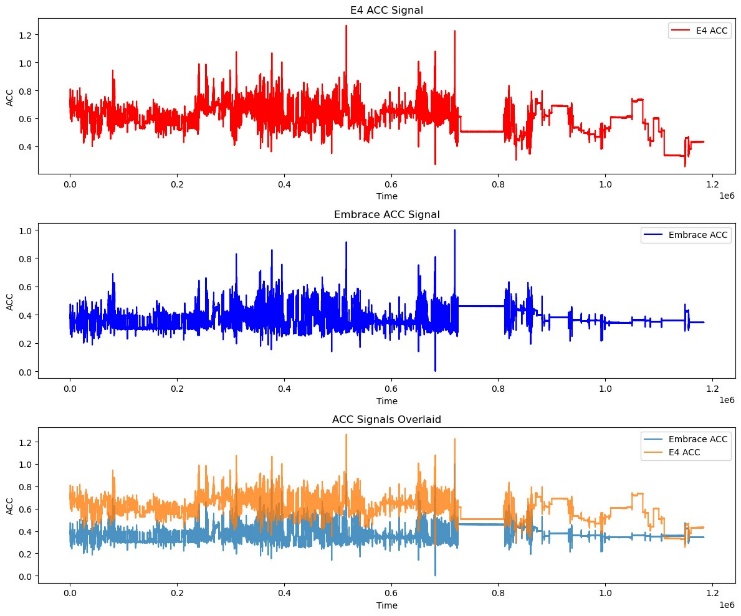

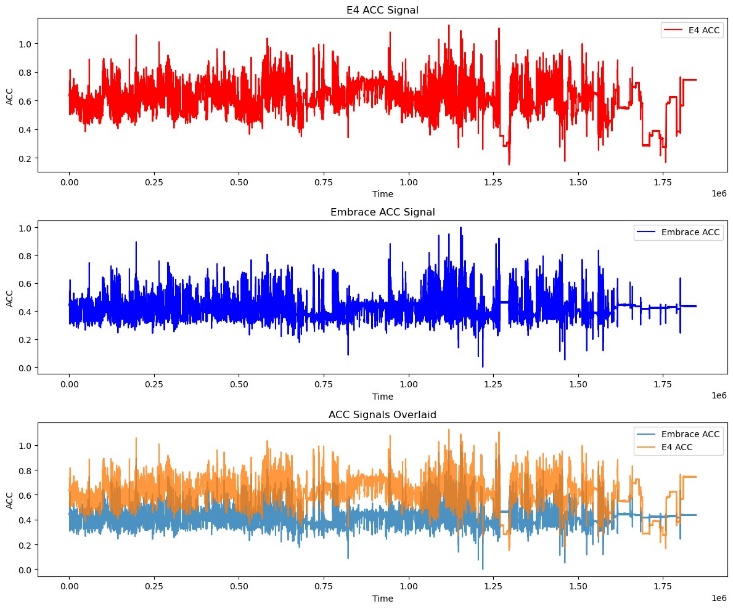


1. Participant 2
2. Participant 3

**F I G U R E C15 Raw and Aligned ACC‐x Signals from Embrace and E4 Devices.** These overlays display raw accelerometer x‐axis data from both devices. Participant 2’s signals show notable amplitude divergence and asynchronous peaks, whereas Participant 3 presents improved visual alignment and comparable motion intensity patterns.

Overlaid time‐series plots show that:

- Embrace and E4 signals track similar movement patterns over time, including peaks, bursts, and activity transitions. · Differences in baseline offset and dynamic range are visible, with Embrace appearing slightly “dampened” compared to E4.
- Periods of motion and inactivity are well aligned, further validating the synchronization pipeline.
- The Embrace Plus and E4 provide consistent temporal and spectral patterns in ACC‐X data, supporting cross‐device use in movement intensity and activity phase analysis.
- Minor amplitude discrepancies are evident but do not distort motion trends, making these signals reliable for downstream analytics.


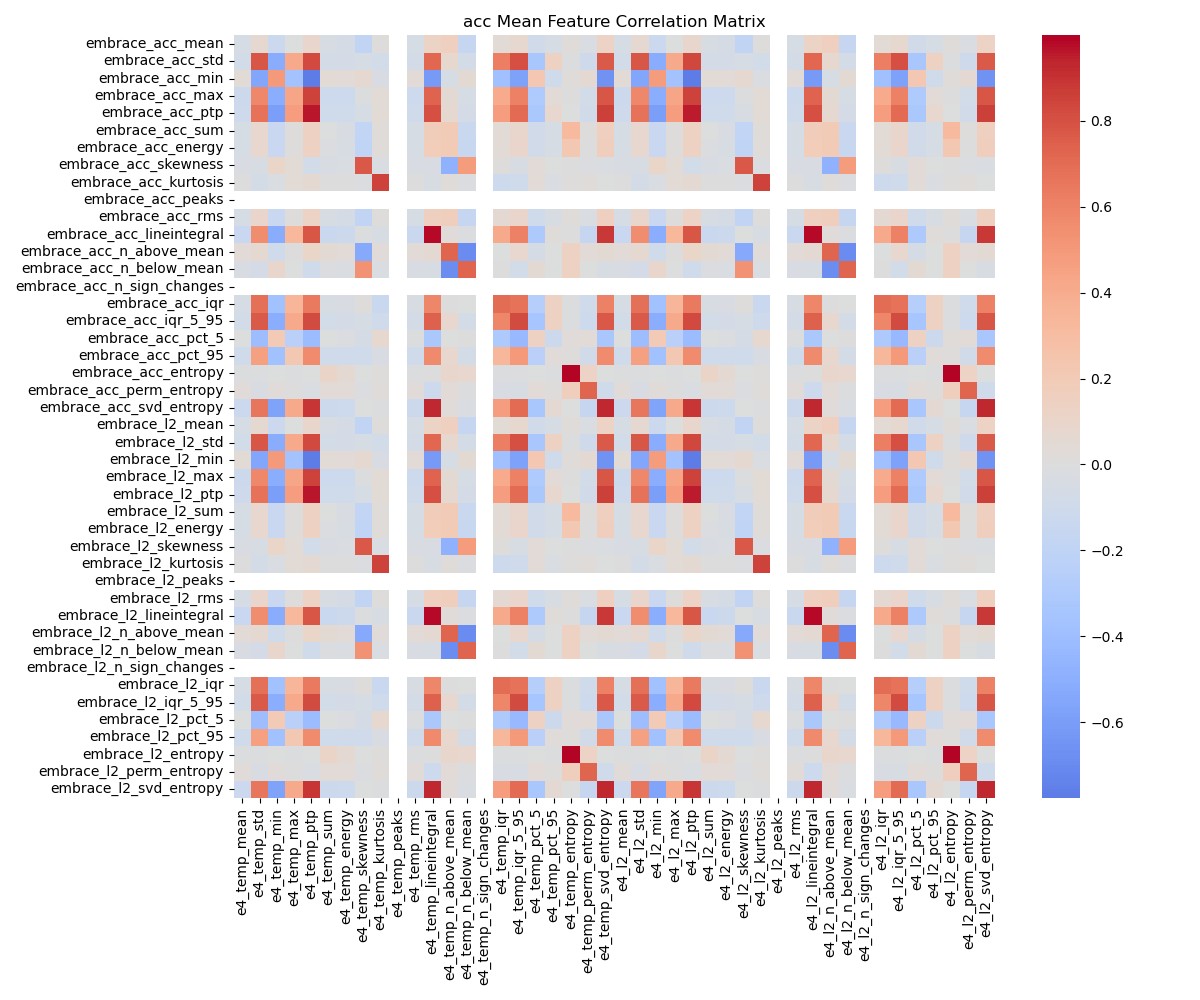
**Y‐axis:** Showed the weakest agreement, with CCC = 0.190, RMSE = 0.120, and Pearson r = 0.094. The corresponding heatmap revealed sparse cross‐feature consistency, likely due to desynchronization or noise.

**F I G U R E C16 Accelerometer Y‐Axis Feature Correlation Matrix.** The weakest agreement among all accelerometer axes is observed here. The sparse and scattered correlation patterns suggest signal misalignment or lower sensor fidelity in the Y direction. This is further supported by low CCC and correlation metrics presented in Section 3.3.

)


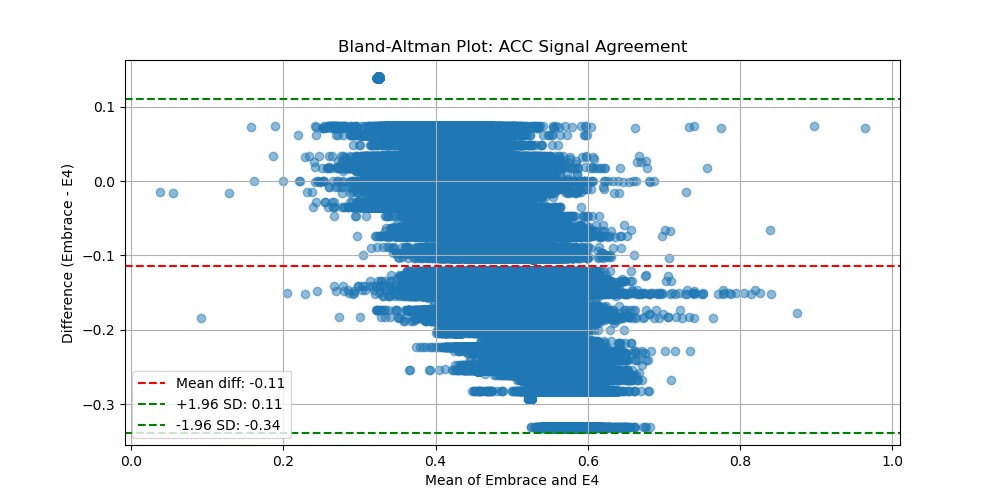

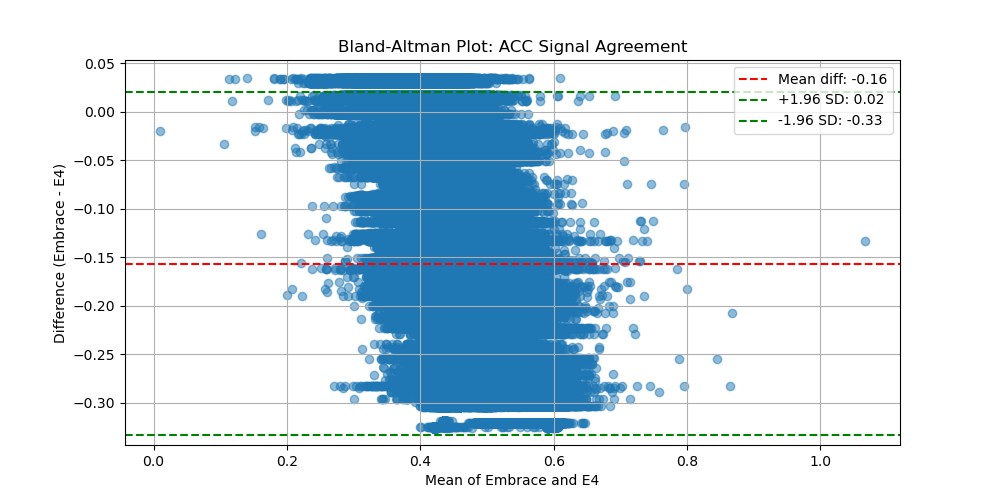


1. Participant 2
2. Participant 3

**F I G U R E C17 Bland–Altman Plots for ACC Y‐Axis Signal Agreement.** These plots show the difference between Embrace and E4 accelerometer (Y‐axis) signals versus their mean. Participant 2 shows a moderate negative bias and wide agreement limits, suggesting signal misalignment. Participant 3 displays tighter limits but still noticeable bias, reflecting variability in motion data across devices.

The mean difference between Embrace and E4 Y‐axis signals hovers around ‐0.16, showing a systematic bias where Embrace consistently reports lower values. The limits of agreement are wider compared to X or Z axes, reflecting greater inter‐device variability. No evident funnel shape in the spread (heteroscedasticity), but dense clustering around lower means suggests that low‐magnitude movements are especially prone to disagreement. This result flags a device‐specific baseline offset or differential sensitivity to lower amplitude motion along the Y‐axis. Part of this discrepancy may also stem from positional differences: the E4 was worn more proximally (closer to the forearm), while the EmbracePlus was worn distally (nearer to the wrist). For instance, during activities such as typing or using a mouse, which primarily involve forearm movements, the more proximal E4 might detect subtle flexion/extension that the wrist‐level Embrace may not capture as strongly. Prior research on wrist‐worn accelerometers highlights that even slight changes in device position (top vs bottom of the wrist) can significantly affect the measured acceleration vector and activity estimates (Polo et al., 2019; van Hees et al., 2013).

We assessed the agreement between accelerometer signals, y-axis, recorded by EmbracePlus and E4 using percentage error derived from Bland–Altman limits of agreement. Following the same formula used for other modalities. The resulting average percentage errors were approximately 40.0%. The y axis exceeds the acceptable threshold of 30.0%, suggesting greater measurement divergence across devices.


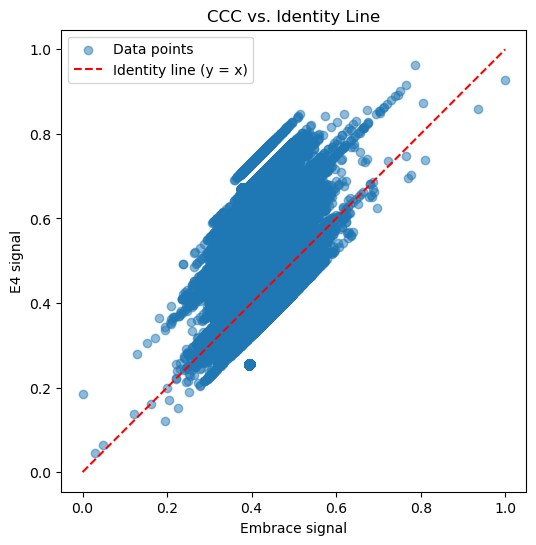

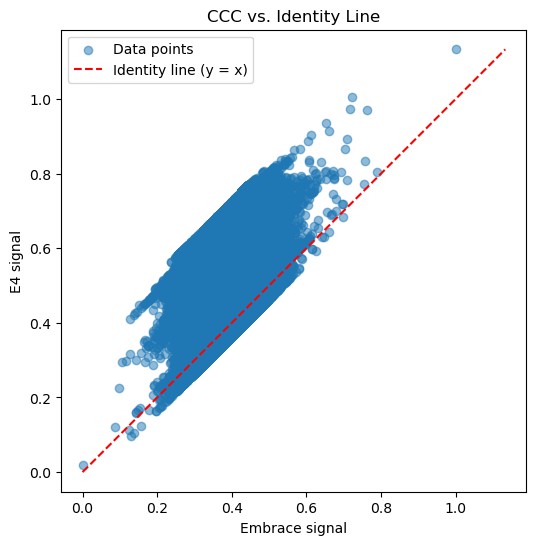


1. Participant 2
2. Participant 3

**F I G U R E C18 Concordance Correlation Coefficient (CCC) Identity Plots for ACC Y‐Axis.** These scatter plots compare Y‐axis accelerometer values between Embrace and E4. Participant 2’s signal deviates significantly from the identity line, indicating weak agreement. Participant 3shows a tighter cluster around the identity line, suggesting stronger linear concordance.


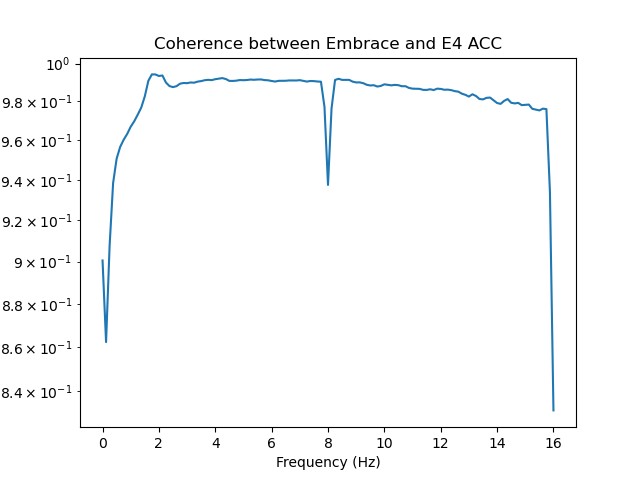

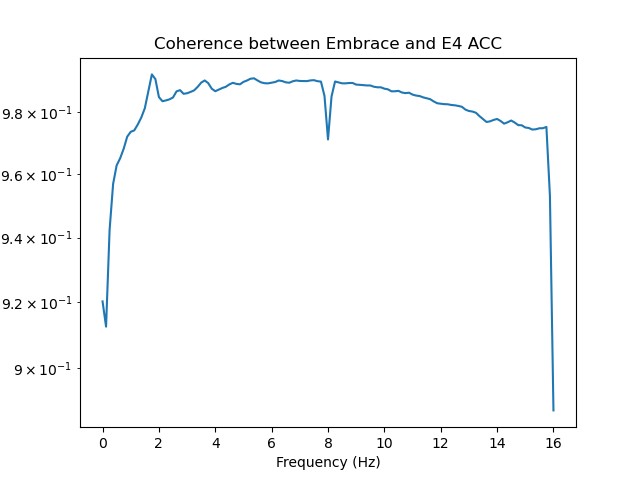


1. Participant 2
2. Participant 3

**F I G U R E C19 Spectral Coherence of Y‐Axis Accelerometer Signals.** The plots display coherence across frequencies between Embrace and E4. Participant 2 shows relatively high coherence but with greater variability. Participant 3 has more stable coherence near 0.98 across most frequencies, indicating consistent frequency‐domain alignment.

High coherence (> 0.97) across most frequencies, with a sharp drop at 8 Hz due to likely anti‐aliasing or filter artifacts common in inertial sensing pipelines. Despite low amplitude agreement, spectral coherence indicates that the two signals share strong temporal alignment in frequency dynamics, especially at low‐frequency bands (< 10 Hz) relevant for physical movement. Functional alignment is preserved—the timing of events and rhythmicity are similar even if the magnitude varies.


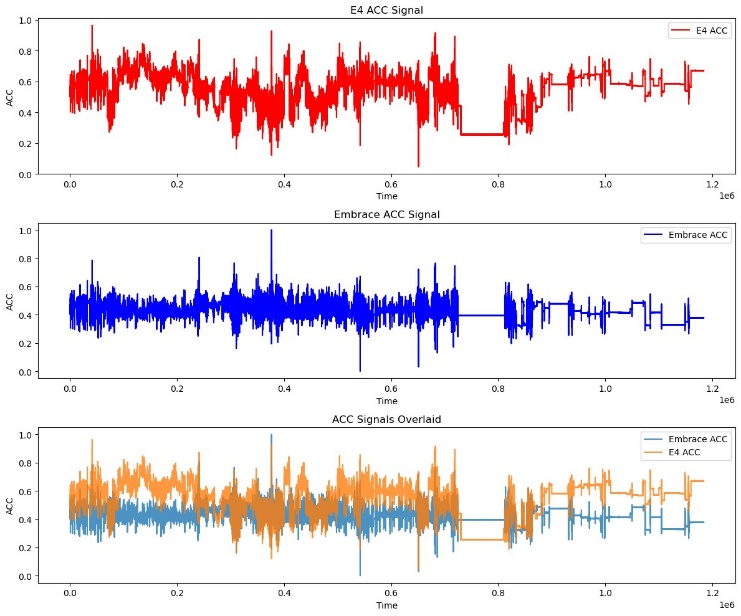

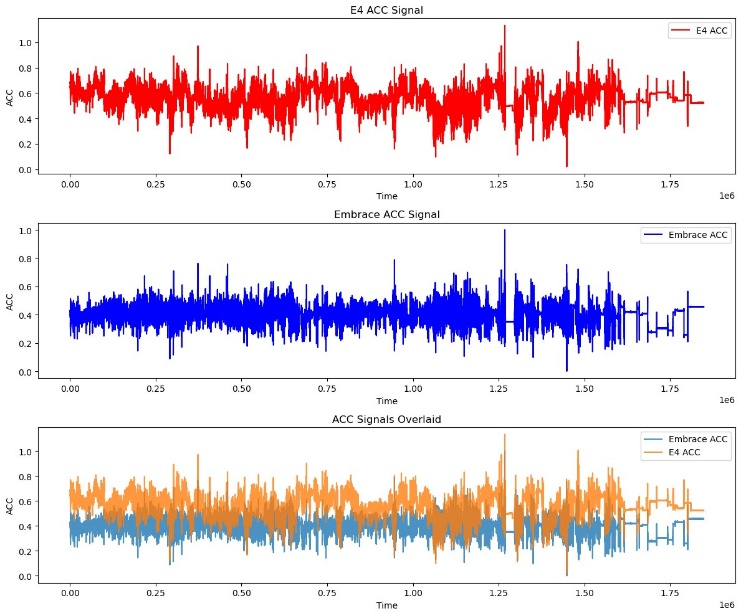


1. Participant 2
2. Participant 3

**F I G U R E C20 Raw and Aligned Y‐Axis ACC Signals.** Time series plots for Embrace and E4 accelerometer data (Y‐axis). Participant 2 exhibits larger deviations and poorer overlap, while Participant 3 demonstrates improved synchrony and similar activity patterns, though some signal jitter is still evident.

Raw Y‐axis signal overlays (see plots C20) exhibit considerable amplitude offsets and baseline drifts between devices. The E4 device generally shows higher peaks and broader excursions than Embrace Plus. Signal transitions (e.g., during motion bursts) occur around the same times, reinforcing high coherence, but peak magnitudes and resting baselines differ. This suggests that the Embrace sensor under‐reports Y‐axis acceleration, or applies stronger damping/smoothing. Notably, the Y‐axis on wrist‐worn accelerometers typically points toward the radial side of the arm (i.e., toward the thumb) (Park et al., 2023). Positional or angular differences between the two devices on the wrist may therefore disproportionately affect readings along this axis, especially during lateral wrist movements.

**Z‐axis:** Delivered the strongest performance among axes, with CCC = 0.850, RMSE = 0.064, Pearson r = 0.808, and coherence = 0.988. This suggests robust synchronization for vertical motion signals.


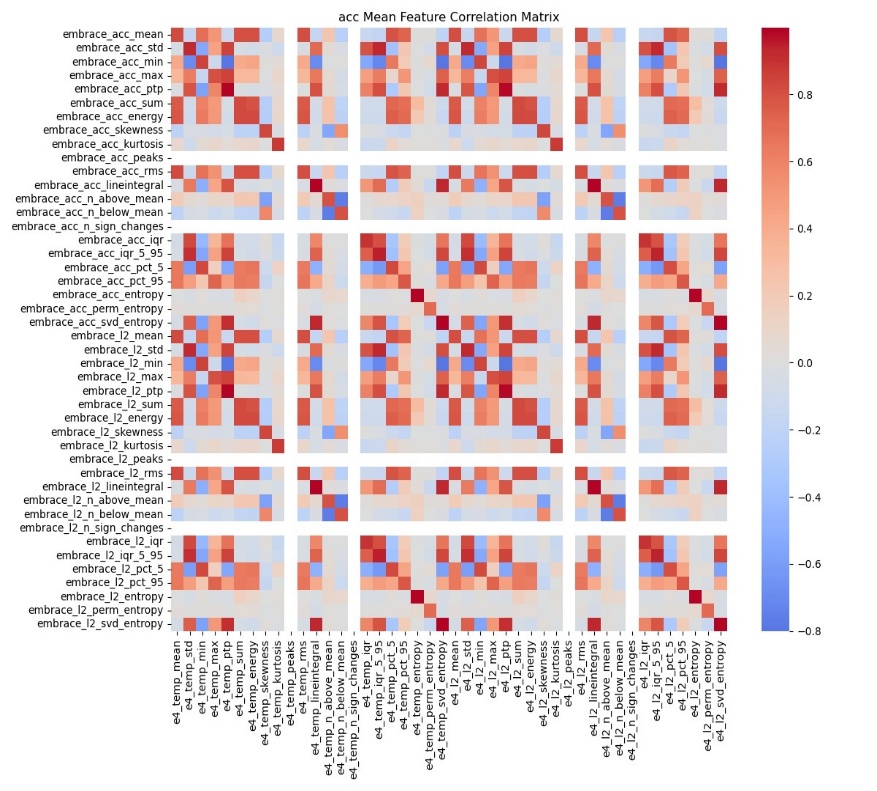


**F I G U R E C21 Accelerometer Z‐Axis Feature Correlation Matrix.** Among all axes, the Z‐axis demonstrates the highest feature correlation, particularly in magnitude and energy‐related features. This is consistent with high CCC and coherence metrics reported in Section 3.3, indicating that vertical motion is well‐captured by both devices.

Key observations from the Figure C21 are the following:

- Moderate correlations are seen between statistical features (e.g., mean, std, rms), with ≈ 0.4 – 0.6, suggesting relatively consistent value ranges.
- Entropy‐based metrics (e.g., entropy, perm_entropy ) show inter-device correlation up to ≈ 0.7, indicating complexity patterns are partially preserved across devices.
- However, sign change and peak‐based features exhibit weak alignment, likely reflecting differences in sensor sampling noise or minor misalignments.


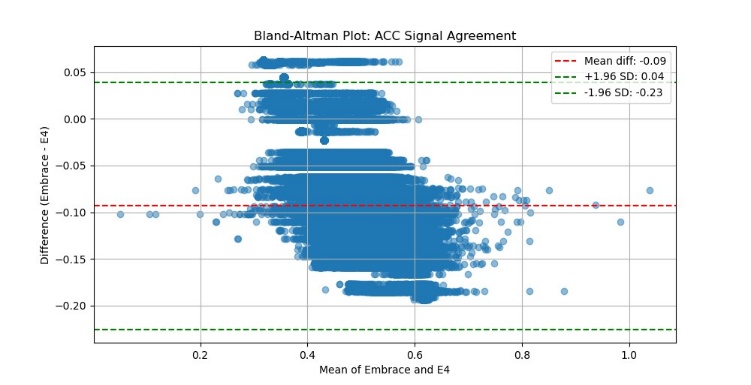

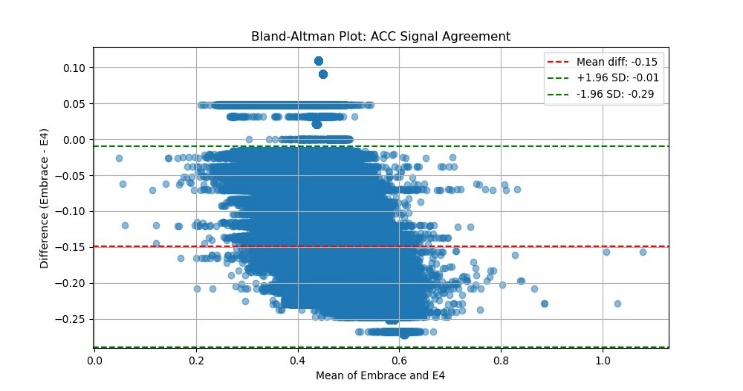


1. Participant 2
2. Participant 3

**F I G U R E C22 Bland–Altman Plots for ACC‐Z Signal Agreement.** The plots assess the agreement between Embrace and E4 accelerometer signals along the Z‐axis. Participant 2 shows slightly tighter agreement with a smaller mean difference and narrower limits of agreement, while Participant 3 exhibits more dispersion and a larger negative bias.

Mean difference ranges from −0.09 to −0.15, with narrow confidence intervals, indicating reasonably stable agreement. The presence of negative bias suggests the Embrace device consistently reports lower Z‐axis magnitude, possibly due to baseline calibration differences. Agreement narrows around the mid‐range of activity (mean ≈ 0.4–0.7), implying that during moderate motion both devices are most synchronized. We assessed the agreement between accelerometer signals, z‐axis, recorded by EmbracePlus and E4 using percentage error derived from Bland–Altman limits of agreement. Following the same formula used for other modalities. The resulting average percentage errors were approximately 28.5%. While the Z‐axis error remains just within the commonly accepted ±30% threshold for agreement (Critchley et al., 1999), the X and Y axes exceed this threshold, suggesting greater measurement divergence across devices. These results may reflect differences in sensor orientation, placement, or device‐specific preprocessing pipelines for motion data. Accordingly, care should be taken when directly comparing raw acceleration signals across devices, particularly for applications requiring high temporal or directional accuracy.


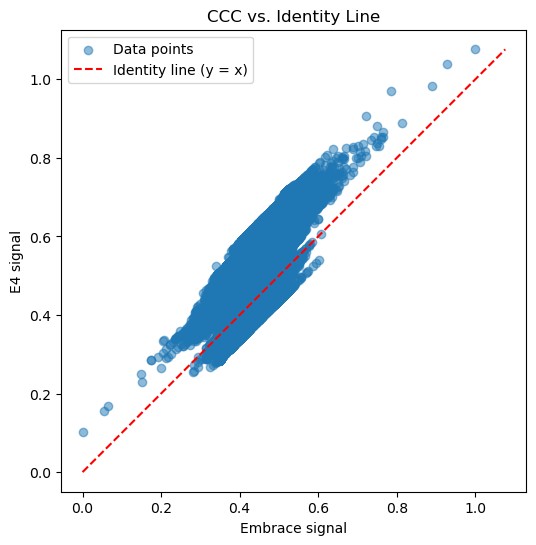

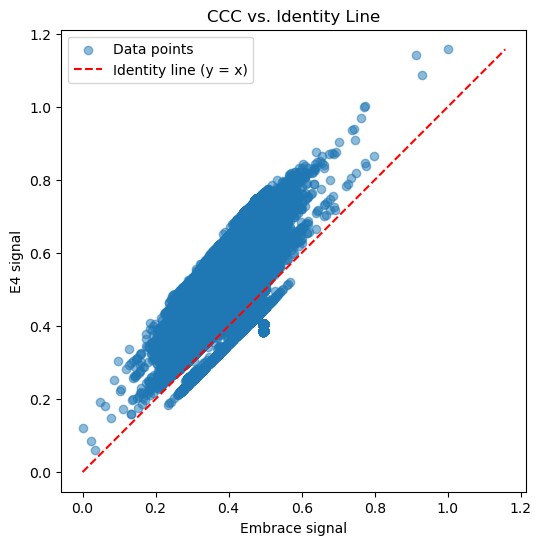


1. Participant 2
2. Participant 3

**F I G U R E C23 CCC Identity Plots for ACC‐Z Signals.** These scatter plots compare ACC‐Z values between the Embrace and E4 devices. Participant 2’s data points cluster closer to the identity line *y* _=_ *x*, indicating stronger agreement, while Participant 3 shows greater variability and less alignment, suggesting signal disparity.

Participants display moderate CCC (range ≈ 0.55–0.65), indicating reliable but not perfect agreement. Deviations from the red line imply some level of systematic offset or scale mismatch. CCC values support the Bland–Altman findings: consistent bias with moderate scale alignment. E4 signal tends to overshoot slightly, possibly reflecting differences in sensor sensitivity or signal scaling post‐filtering.

1. Participant 2
2. Participant 3


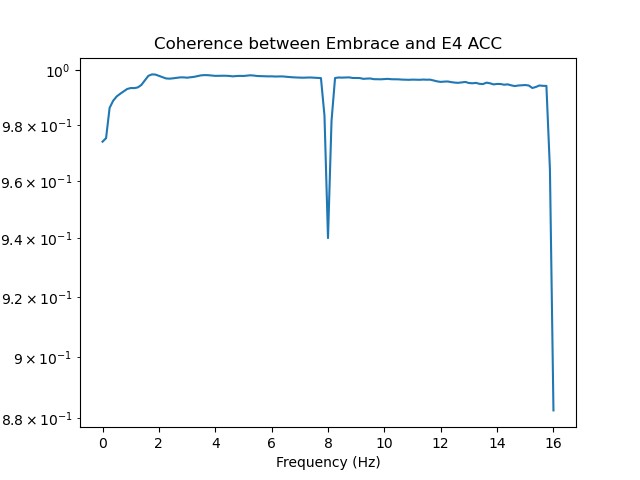

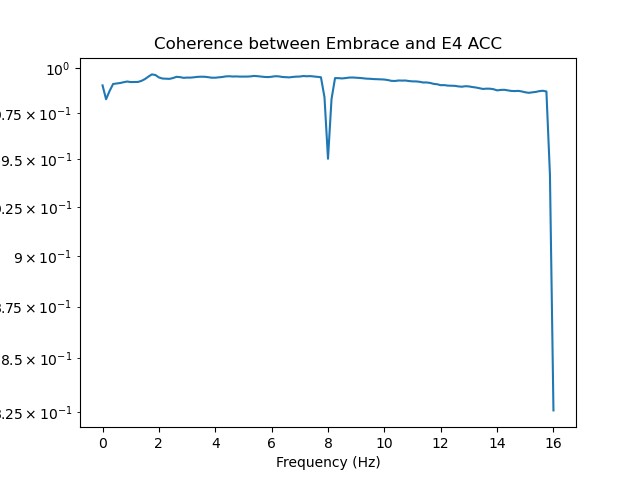


**F I G U R E C24 Spectral Coherence Between Embrace and E4 ACC‐Z Signals.** The coherence spectra indicate strong frequency‐wise similarity between the two devices across most frequencies. Participant 2 maintains consistently high coherence (> 0.95), while Participant 3 shows more noticeable dips, hinting at intermittent misalignment.

High coherence (>0.97) suggests strong spectral alignment, reinforcing that dynamic activity cycles (e.g., steps, posture shifts) are similarly tracked in frequency. The coherence dip may correspond to frequency notches applied during preprocessing or low sensor SNR at certain ranges.


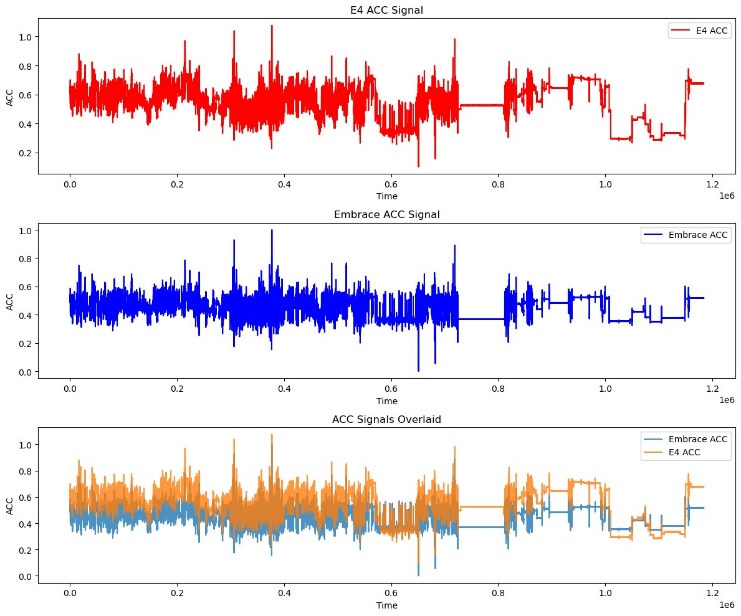

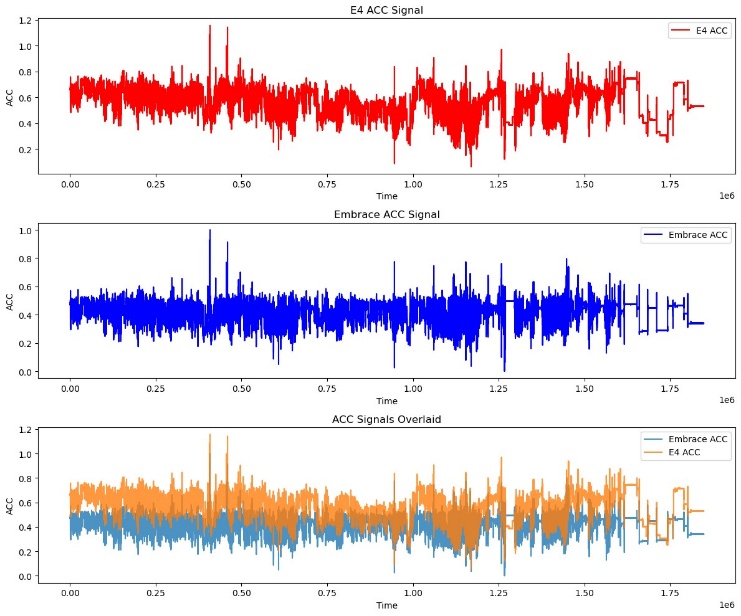


1. Participant 2
2. Participant 3

**F I G U R E C25 Raw and Aligned ACC‐Z Signals from Embrace and E4 Devices.** Each panel presents the individual and overlaid accelerometer Z‐axis signals from both devices. Participant 2 shows high temporal and amplitude synchrony, whereas Participant 3 displays phase shifts and amplitude discrepancies, highlighting inter‐device variation.

The raw overlay shows that temporal structure is preserved: peaks and troughs align closely. However, amplitude inconsistencies exist, especially at activity bursts, indicating potential differences in sensor gain or orientation during wear. These findings highlight axis‐dependent alignment quality, with implications for downstream analyses involving motion features.

**D TEMPERATURE ANALYSIS**

The temperature signals showed moderate agreement across statistical metrics and features (Figure 6). The RMSE (0.177) and MAE (0.161) indicate measurable amplitude differences, while CCC (0.755), MI (3.320), and NMI (0.739) reflect reasonably strong structural similarity. A KL divergence of 0.490 (±0.419) and Pearson r = 0.755 indicate some distributional and linear differences. However, phase locking (PLV = 0.982) and coherence (0.723) were high, confirming consistent phase and frequency alignment, albeit with greater variability than in BVP or EDA signals.

The temperature signals recorded by the EmbracePlus and Empatica E4 devices showed moderate agreement across a variety of statistical, distributional, and frequency‐domain metrics. While overall structural similarity was preserved, measurable amplitude offsets and slight variability in temporal response were observed.

**
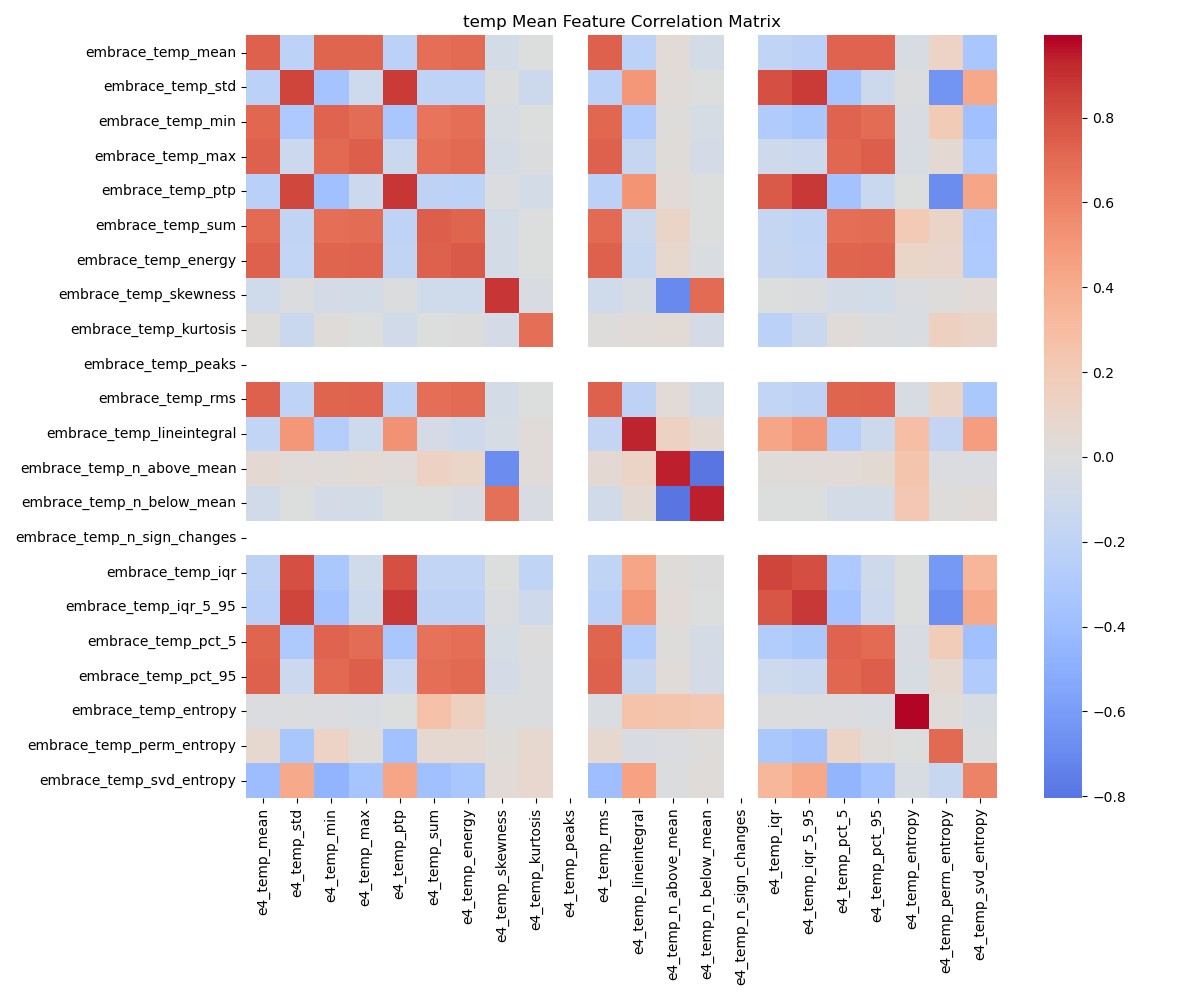
**

**F I G U R E D26 Temperature Feature Correlation Matrix.** The temperature signal features show moderate agreement. Notable consistency is observed in basic statistics (mean, std, RMS), but higher variability is present in entropy‐based metrics. These findings align with observed amplitude differences and variability in the distribution, as discussed in Section 3.4.

The temperature signal features show moderate agreement. Notable consistency is observed in basic statistics (mean, std, RMS), but higher variability is present in entropy‐based metrics.

Key observations:

- Strong cross‐device correlation for mean, standard deviation, RMS, and sum features (*r* > 0.8).
- Lower agreement in entropy‐based metrics (e.g., permutation entropy, SVD entropy), suggesting differences in complexity estimation between devices.
- These results support the interpretation that global trends are well captured, while fine‐grained fluctuations vary more across devices.


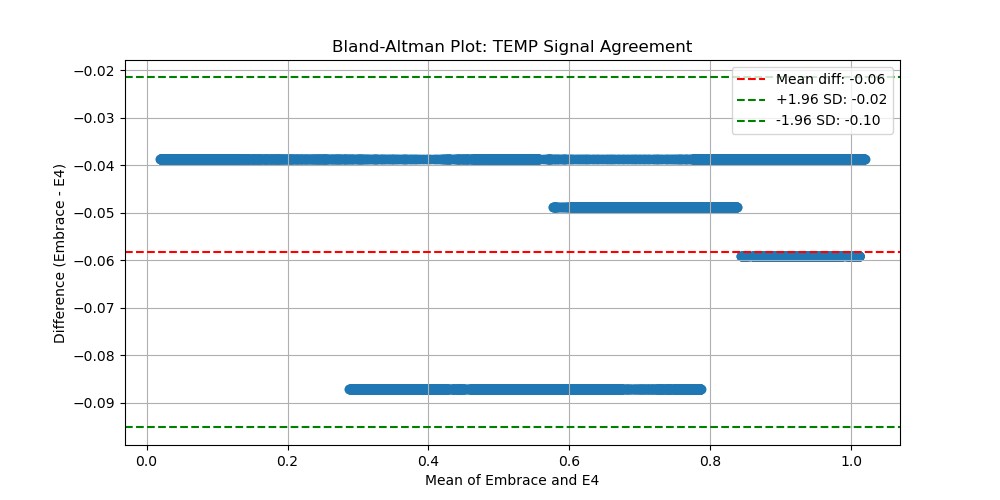

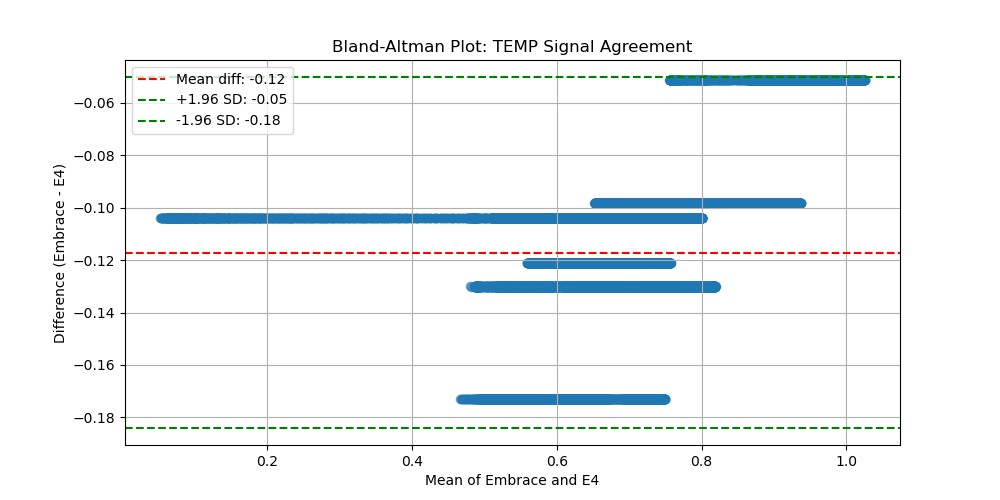


1. Participant 2
2. Participant 3

**F I G U R E D27 Bland–Altman Plots for TEMP Signal Agreement.** These plots compare the mean temperature values from Embrace and E4 against their difference. Participant 2 (Mean difference: −0.06, Limits of agreement: [−0.10,−0.02], Indicates a small, consistent underestimation by Embrace vs. E4.) displays tighter agreement limits and less spread, while Participant 3 (Mean difference: −0.12, Limits: [−0.18,−0.05], A wider spread indicates higher variability, but still within expected thermal signal ranges.) shows slightly greater variability, though the mean difference remains small in both cases.

There is a consistent negative bias across participants, meaning EmbracePlus generally reports lower temperature values than E4. The spread is tight and symmetric, indicating stable bias with no major outliers or proportional error. We also computed the percentage error for temperature measurements to assess agreement between the EmbracePlus and E4 devices. Using the same Bland–Altman–based formula:

$Percentage Error \left( \% \right)= \frac{1.96x{SD}_{diff}}{Mean of Means}x100$, The resulting average percentage error was approximately 17.5%. This value falls well within the commonly used ±30% agreement threshold (Critchley et al., 1999), indicating good consistency between temperature sensors across the two devices. Compared to other physiological signals, temperature readings demonstrated relatively stable agreement, suggesting that thermal sensing is less affected by placement or device‐specific factors in this setup.


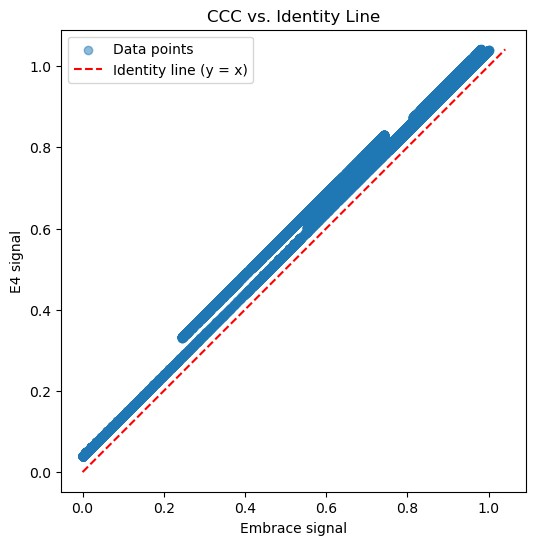

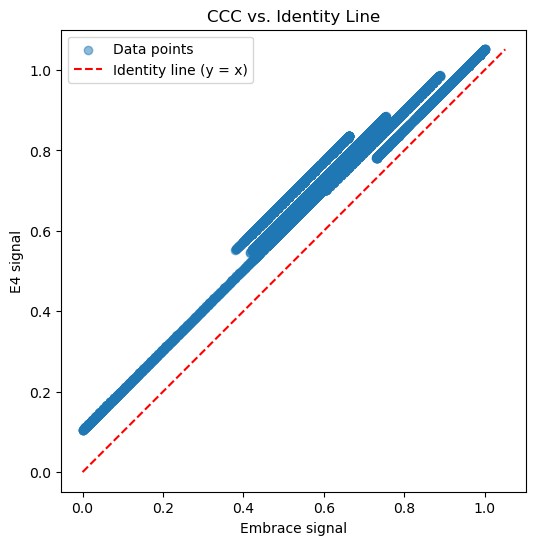


1. Participant 2
2. Participant 3

**F I G U R E D28 Concordance Correlation Coefficient (CCC) Identity Plots for TEMP Signal.** Participant 2 exhibits near‐perfect alignment with the identity line (*y* _=_ *x*), suggesting strong agreement between devices. Participant 3 also shows tight clustering, though with a small vertical deviation, indicating slightly lower agreement than Participant 2.

Points lie very close to the identity line, indicating strong concordance. Slight vertical shift reflects the small systematic offset noted above. Concordance Correlation Coefficient (CCC) = 0.755, confirming reliable tracking of temporal structure with some amplitude differences.


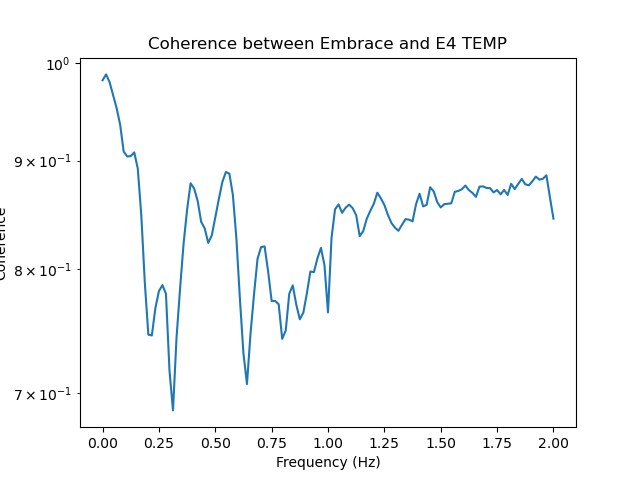

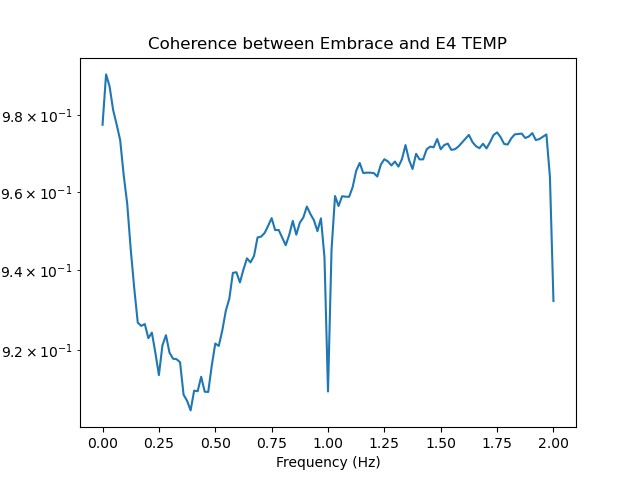


1. Participant 2
2. Participant 3

**F I G U R E D29 Spectral Coherence Between Embrace and E4 TEMP Signals.** Both participants show high coherence values across most of the frequency spectrum, indicating consistent signal structure between devices. Participant 2 exhibits several noticeable drops in coherence below 0.9 in the low‐to‐mid frequency range (0.2–1.2 Hz), suggesting intermittent desynchronization in slower signal fluctuations. In contrast, Participant 3 shows more stable coherence across all frequencies, consistently above 0.91, indicating tightly coupled temperature signals.

Coherence plots show values between 0.72 and 0.98, indicating strong alignment in the frequency domain. This confirms that both devices capture similar temporal patterns, even if the absolute values differ. Minor dips at very low frequencies likely reflect different filtering behaviors or thermal inertia between the sensors.


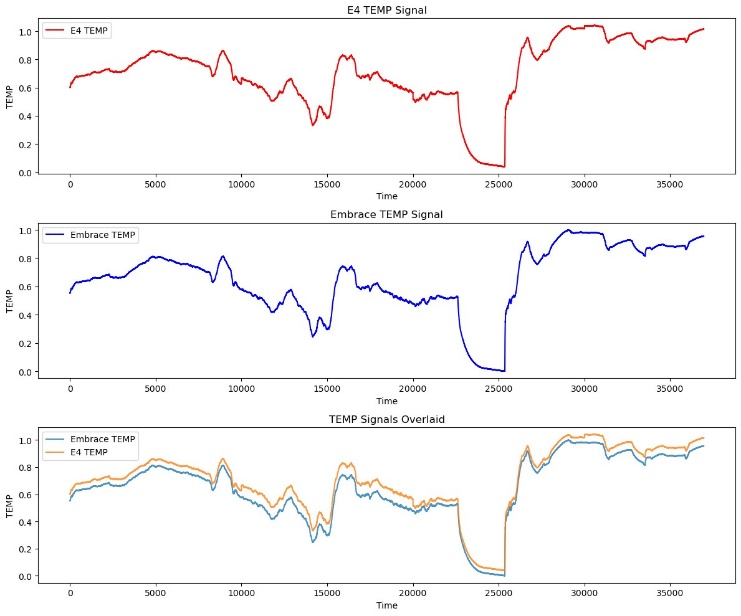

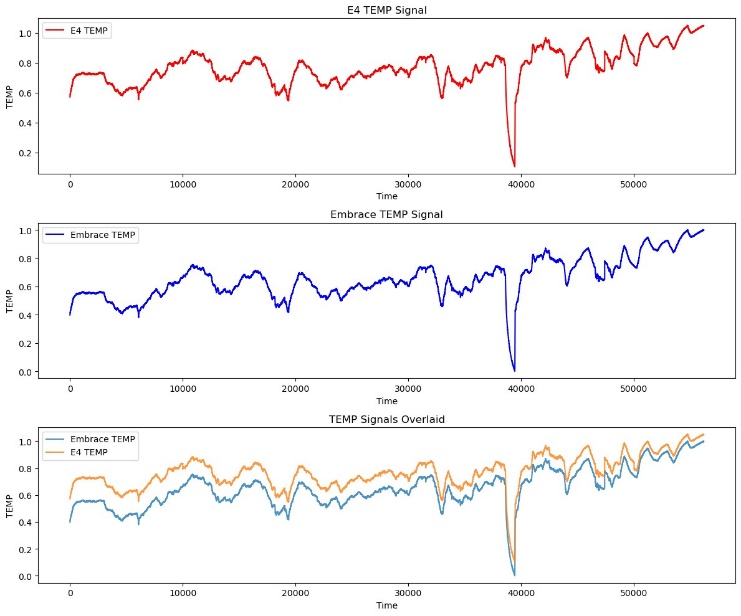


1. Participant 2
2. Participant 3

**F I G U R E D30 Raw and Aligned TEMP Signals from Embrace and E4 Devices.** Signal overlay plots indicate close temporal and amplitude alignment between devices. Participant 2 shows almost identical waveforms with minor amplitude offsets, while Participant 3 retains shape similarity but with slightly more deviation across time. Overlaid time series are tightly matched in phase. Clear tracking of temperature dips and peaks across time. EmbracePlus signals consistently exhibit slightly lower amplitude, supporting the bias seen in Bland‐Altman and CCC plots.

The temperature signals from EmbracePlus and E4 show moderate to strong agreement, particularly in structural and spectral domains. Small, consistent amplitude differences are observed, likely due to calibration offsets or sensor placement. For applications where relative changes or temporal dynamics are key (e.g., thermoregulation, stress detection), the alignment is likely sufficient. However, absolute temperature values should be interpreted with caution across devices.

**E. USE-CASE 2 : CROSS-VENDOR SYNCHRONIZABILITY IN LARGE-SCALE STUDIES**

While Appendix [A-D] demonstrated synchronizability between wearable devices produced within the same vendor ecosystem (Empatica E4 and Embrace Plus), large-scale and longitudinal studies increasingly rely on heterogeneous sensing platforms due to cost, availability, participant burden, and evolving study designs. Addressing synchronization and validation across independent device manufacturers is therefore critical for promoting equitable, scalable, and vendor-agnostic physiological research. To illustrate these challenges and opportunities, this section examines the synchronizability of the Zmax and Empatica E4 devices as deployed within the Healthy Brain Study.

**E.1. THE HEALTHY BRAIN STUDY: CONTEXT AND SCALE**

The Healthy Brain Study is a large (Healthy Brain Study consortium & Haak, 2021), population-based cohort study, which includes 861 participants aged between 30-39, designed to investigate the biological, behavioral, and environmental determinants of brain health across the adult lifespan. Due to its scale and longitudinal nature, the study prioritizes:

- **Remote and ambulatory data collection**
- **Minimally intrusive wearable technologies**

As a result, multiple wearable devices with distinct hardware architectures, firmware designs, and data pipelines are often used either concurrently or across study phases. This creates a pressing need for robust synchronization strategies that do not assume shared vendor infrastructure or proprietary time-alignment mechanisms. In this use case, physiological data were collected using:

- **Empatica E4**, a research-grade wrist-worn device providing electrodermal activity (EDA), photoplethysmography (PPG), accelerometry, and skin temperature.
- **Zmax**, a wearable, headband, EEG-based system primarily designed for sleep and neurophysiological monitoring, providing EEG, EOG, EMG, and auxiliary signals.

Unlike the Empatica E4 and Embrace Plus pairing discussed in Appendix [A-D], these devices:

- Originate from independent manufacturers
- Use distinct internal clocks
- Rely on separate data acquisition and storage pipelines
- Worn on different locations on the body
- Offer limited or no access to low-level clock synchronization mechanisms

Despite differences in hardware design and primary sensing modalities, the Empatica E4 and Zmax devices share common physiological biomarkers, most notably **triaxial accelerometry** and **photoplethysmography (PPG)**. These overlapping signals provide a common basis for cross-device temporal alignment and validation. Importantly, the same preprocessing, synchronization, and validation pipeline described in the main manuscript was applied uniformly to both devices, without device-specific adaptations. This demonstrates that the proposed methodology is not tailored to a particular vendor or hardware configuration, but is instead applicable across heterogeneous wearable platforms. Consequently, synchronizability must be established post-hoc using signal-based or event-based alignment methods rather than relying on shared timestamps or synchronized start commands. This case study demonstrates that meaningful synchronization is feasible even when combining wearable devices from different vendors, provided that transparent validation procedures are applied. Importantly, this approach:

- Reduces dependence on proprietary ecosystems
- Enables integration of cost-diverse devices across participant populations
- Supports inclusive study designs where device choice may be constrained by availability, participant preference, or regional accessibility

**E.2. BLOOD VOLUME PULSE ANALYSIS**

After applying the same synchronization and preprocessing pipeline used in the main manuscript, BVP/PPG signals showed strong waveform correspondence between Zmax and Empatica E4 (Pearson *r* = 0.848; CCC = 0.848). Absolute amplitude differences were moderate (MAE = 0.091; RMSE = 0.170, in normalized units), and distributional similarity was moderate (KL divergence = 0.622), indicating that while temporal structure was preserved, the signals were not perfectly interchangeable in absolute amplitude.


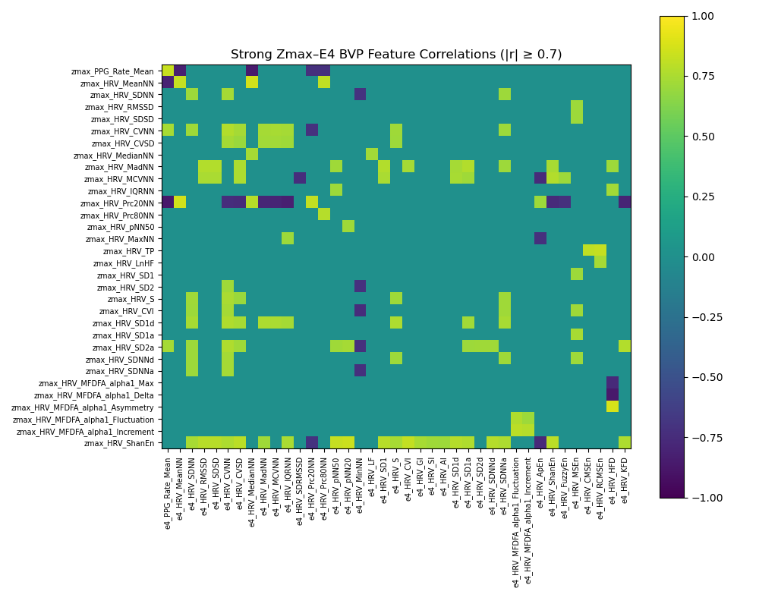


**F I G U R E E1 Correlation Matrix for the BVP features of Zmax and Empatica E4.** The correlation matrix showing the features with correlation score greater than 0.7 for visualization purposes.

Figure E1 shows the cross-device feature correlation matrix for BVP-derived biomarkers after alignment, thresholded at |r| ≥ 0.7 to emphasize robust associations. Strong positive correlations were observed for several canonical heart-rate and variability features, indicating that temporal fluctuations in these biomarkers were preserved across devices. Notably, expected inverse relationships were also recovered (e.g., mean pulse rate versus mean interbeat interval), yielding strong negative correlations consistent with physiological definitions. In contrast, fewer high-magnitude correlations were observed among entropy- and fractal-based complexity features, suggesting greater sensitivity of these measures to device-specific signal characteristics and processing differences.


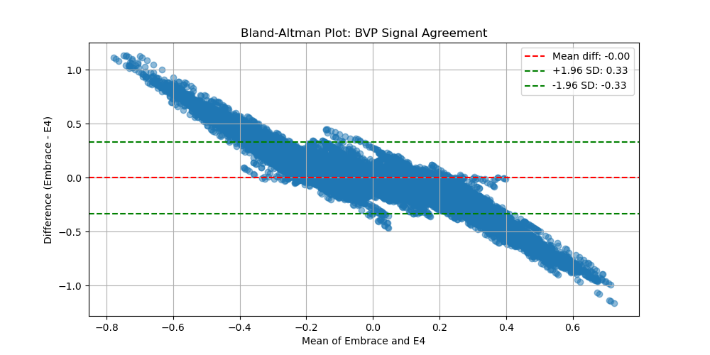


**F I G U R E E2 Bland-Altman Plot for the BVP features of Zmax and Empatica E4.**

When Bland–Altman analysis was performed on 60-second window-averaged BVP values to reduce temporal autocorrelation, the mean bias was effectively zero, with narrow limits of agreement (±0.0072, normalized units). These results indicate that any residual amplitude differences between devices average out over short physiological windows, supporting strong agreement in mean BVP levels after synchronization.


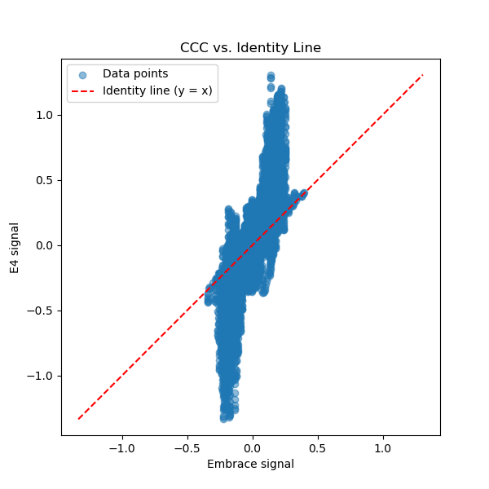

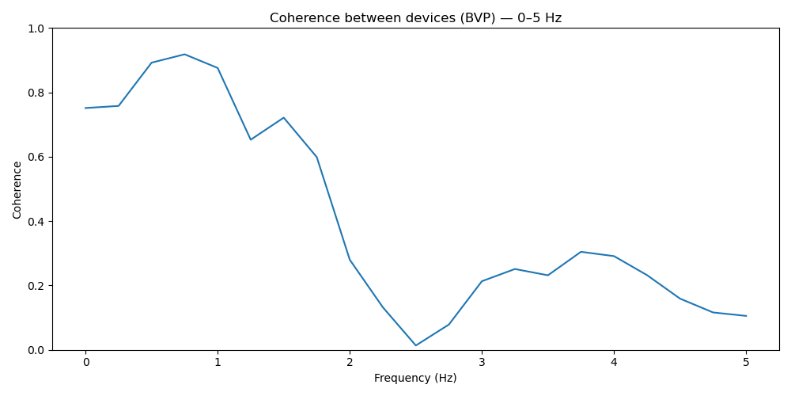


**F I G U R E E3 CCC Identity and Coherence Plot for the BVP features of Zmax and Empatica E4.**

Phase synchronization in the cardiac band was high (PLV = 0.910), indicating strong consistency in the instantaneous phase relationship between the aligned signals. This supports that the alignment preserved cardiac-cycle timing (phase dynamics), even when amplitude- and distribution-based agreement metrics indicated residual device differences. Agreement between aligned BVP signals was further quantified using the concordance correlation coefficient (CCC). A CCC of 0.85 indicated good concordance, reflecting strong linear association combined with limited scale and offset differences. This was visually supported by identity plots, which showed clustering of sample points along the line of identity with moderate dispersion, consistent with amplitude scaling differences rather than temporal misalignment. Frequency-domain agreement was assessed using magnitude-squared coherence. High coherence was observed at low frequencies corresponding to cardiac dynamics, while coherence decreased substantially at higher frequencies. The resulting mean coherence across the analyzed band was moderate (0.43), indicating that although temporal and phase alignment were preserved, amplitude consistency varied across frequencies. This pattern is consistent with device-specific filtering and waveform morphology differences rather than synchronization failure.


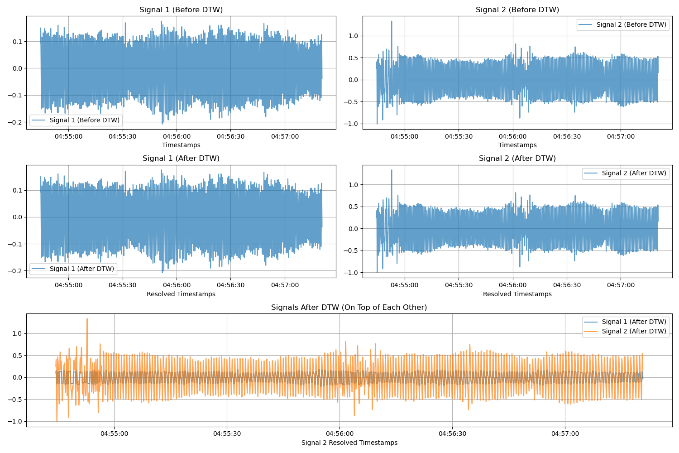

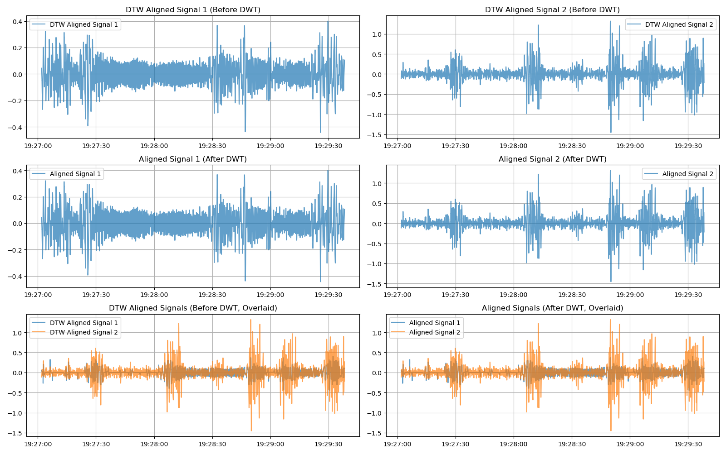


**F I G U R E E4 Examples to Final Aligned Signals for BVP sensors of Zmax and Empatica E4.**

Final aligned BVP signals from the two devices are shown to provide a qualitative illustration of the synchronization outcome. After alignment, the signals exhibit close temporal correspondence, with cardiac pulsations occurring simultaneously across devices. Minor differences in amplitude and waveform morphology remain, reflecting device-specific sensing characteristics and filtering, but the overall timing and structure of the cardiac cycles are preserved. This visual agreement is consistent with the strong phase synchronization and quantitative agreement metrics reported above.

**E.3. ACCELEROMETER ANALYSIS**

Accelerometer signals show varied, but in general lower alignment than the Blood Volume Pulse Signals. The same synchronization, validation and preprocessing pipeline used in the main manuscript, just like the BVP signals and each axis is analyzed separately.

**E.3.1. X-AXIS**

Prior to alignment, accelerometer X-axis signals exhibited no meaningful correspondence (Pearson r ≈ 0; Spearman ρ ≈ 0), reflecting asynchronous acquisition. After alignment, moderate agreement was observed (Pearson r = 0.68; Spearman ρ = 0.49), indicating improved temporal correspondence of movement-related events despite residual amplitude and orientation-dependent differences. Phase synchronization, quantified via PLV, was low (PLV = 0.33), reflecting the inherently non-periodic and event-driven nature of accelerometer signals. This result is expected, due to the location of devices on the body and the characteristics of the Accelerometer signal, and does not indicate poor temporal alignment. Absolute error metrics (RMSE = 0.010; MAE = 0.0045) indicated small pointwise differences after alignment, while a higher KL divergence (3.53) reflected differences in the underlying acceleration distributions, likely driven by sensor orientation and dynamic range disparities.

**
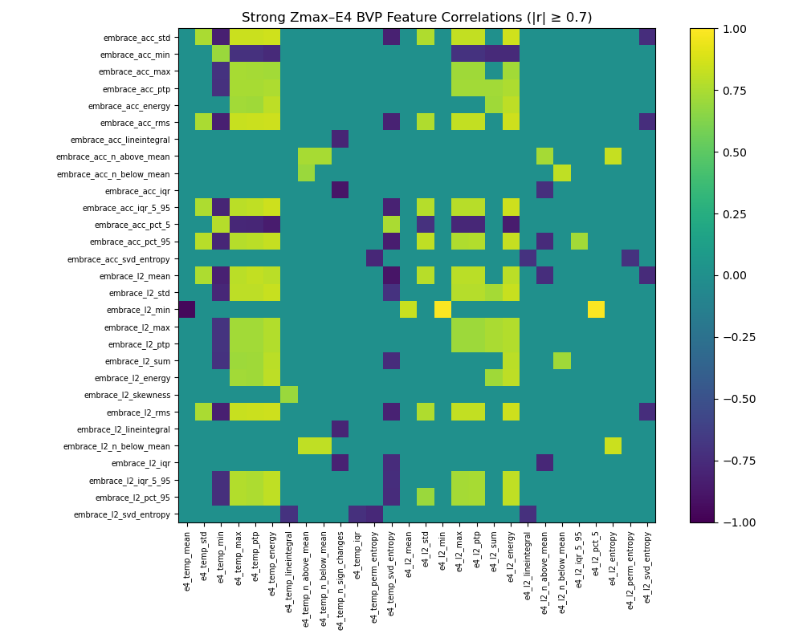
**

**F I G U R E E5 Correlation Matrix for the ACC-X axis features of Zmax and Empatica E4.**

Figure E5 presents the cross-device correlation matrix for accelerometer X-axis features, thresholded at |r| ≥ 0.7 to highlight robust associations. Strong correlations were predominantly observed among amplitude- and energy-based features, including RMS, energy, integral, peak-to-peak, and percentile-based descriptors. These features capture overall movement intensity and were consistently preserved across devices following synchronization. In contrast, directional statistics such as raw mean, minimum, and maximum acceleration exhibited fewer strong correlations, reflecting the orientation-dependent nature of single-axis accelerometer measurements. Notably, L2-norm–based features showed particularly strong agreement, underscoring the advantage of orientation-invariant movement representations for cross-device comparability. Complexity and entropy-based features demonstrated less consistent correlations, consistent with their sensitivity to noise and device-specific signal characteristics. This suggests that, accelerometer synchronization enables reliable comparison of movement features, not raw axis waveforms.

**
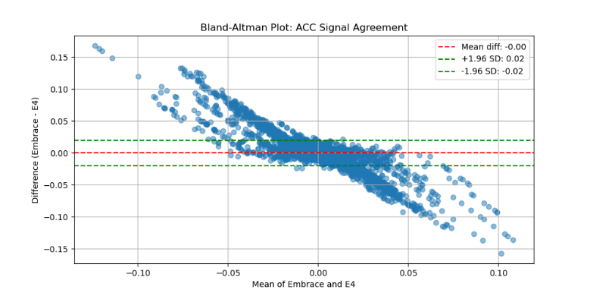
**

**F I G U R E E6 Bland-Altman Plots for the ACC-X axis features of Zmax and Empatica E4.**

Bland–Altman analysis of the accelerometer X-axis showed negligible mean bias and relatively narrow limits of agreement (±0.02, normalized units). However, a pronounced proportional pattern was observed, indicating magnitude-dependent differences consistent with axis orientation and device placement effects.

**
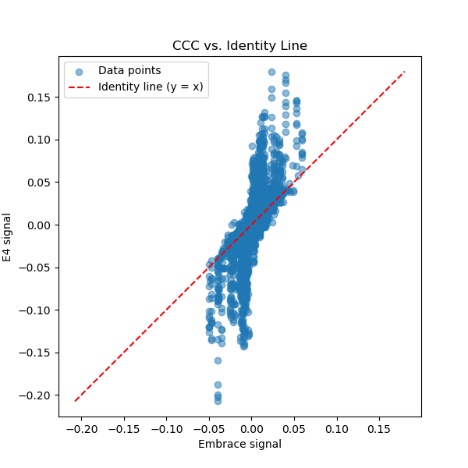
**
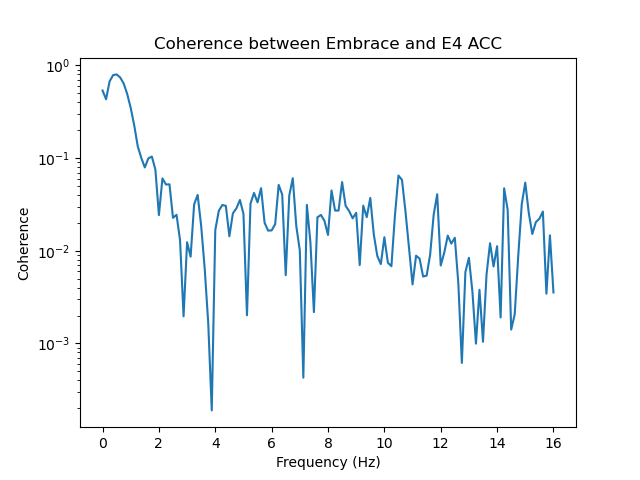


**F I G U R E E7 CCC Identitiy and Coherence Plots for the ACC-X axis features of Zmax and Empatica E4.**

The concordance correlation coefficient further confirmed moderate agreement between aligned accelerometer X-axis signals (CCC = 0.68). Identity plots showed clustering around the line of equality with notable dispersion, consistent with device-specific scaling and orientation effects rather than temporal misalignment. Frequency-domain coherence was low-to-moderate (mean coherence ≈ 0.34), reflecting partial overlap in dominant movement frequencies while highlighting differences in high-frequency content attributable to device-specific filtering and sensor placement. Coherence at very low frequencies corresponding to slow postural and gross movement dynamics, followed by a rapid decay and increased variability at higher frequencies. This pattern reflects the non-periodic, transient nature of accelerometer signals and the strong influence of sensor orientation and placement on single-axis measurements. Consequently, while coherence was limited across much of the frequency spectrum, this does not indicate poor temporal alignment but rather underscores that waveform- and frequency-level equivalence is not expected for accelerometer data across heterogeneous wearable devices. For accelerometry, synchronizability should be evaluated at the level of event timing and derived features, not sustained frequency-domain coupling.

**
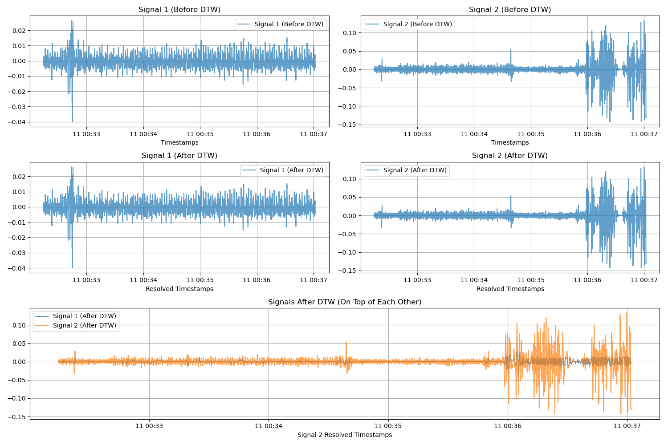

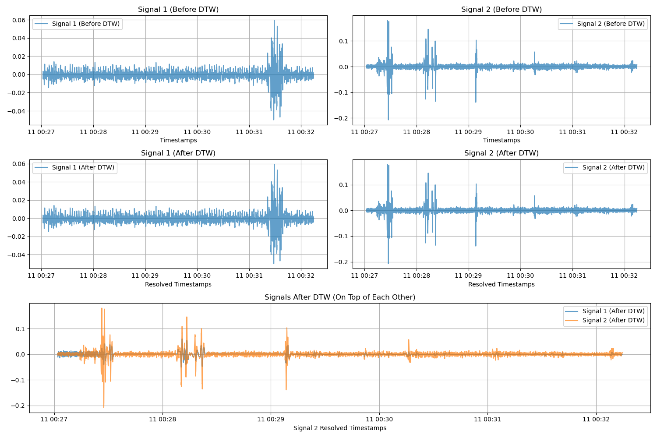
**

**F I G U R E E8 Examples to Final Aligned Signals for ACC X-axis sensors of Zmax and Empatica E4.**

Dynamic time warping further illustrated improved temporal alignment of transient acceleration events. After DTW, movement bursts detected by both devices occurred synchronously, although differences in amplitude and waveform shape persisted.

Overall, accelerometer X-axis signals exhibited clear improvement in temporal correspondence following alignment, as evidenced by increased correlation and moderate concordance. While absolute waveform agreement and phase synchronization remained limited, consistent with the orientation-dependent and non-periodic nature of accelerometer signals, event-level alignment and small pointwise errors support the feasibility of cross-device synchronization for movement detection and activity segmentation, rather than direct amplitude interchangeability.

**E.3.2. Y- AXIS**

Phase synchronization, quantified using the phase-locking value (PLV), was low (PLV = 0.28). This result is expected for accelerometer signals, which are inherently non-periodic and dominated by transient, event-driven dynamics rather than stable oscillations. Consequently, low PLV values do not indicate poor synchronization but rather reflect the limited applicability of phase-based metrics to single-axis accelerometer data. Absolute error metrics indicated small pointwise differences between aligned signals (RMSE = 0.0097; MAE = 0.0035), suggesting that, after normalization, instantaneous discrepancies were limited. In contrast, the high KL divergence (4.68) indicates substantial differences in the overall distribution of acceleration values, likely driven by differences in axis orientation, sensitivity, and dynamic range across devices. This combination—low pointwise error but high distributional divergence, underscores the importance of interpreting accelerometer agreement at the level of relative movement patterns rather than absolute amplitude equivalence.

**
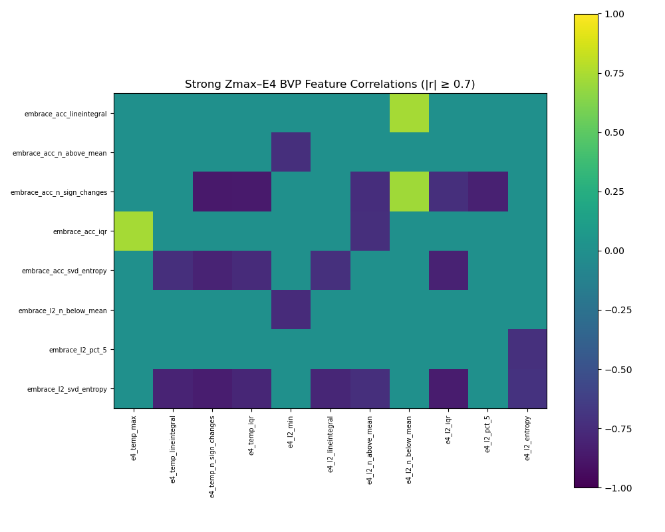
**

**F I G U R E E9 Correlation Matrix for the ACC-Y axis features of Zmax and Empatica E4.**

After alignment, the accelerometer Y-axis signals exhibited moderate linear agreement, with a Pearson correlation coefficient of r = 0.56. This represents a substantial improvement over the pre-alignment case (near-zero correlation) and indicates that temporal correspondence of movement along the Y-axis was partially restored through the synchronization pipeline. Compared to the X-axis, the slightly lower correlation magnitude suggests increased sensitivity of the Y-axis to device orientation, placement, and participant-specific movement patterns. Nonetheless, the observed correlation confirms that prominent movement fluctuations were captured concurrently across devices after alignment.

**
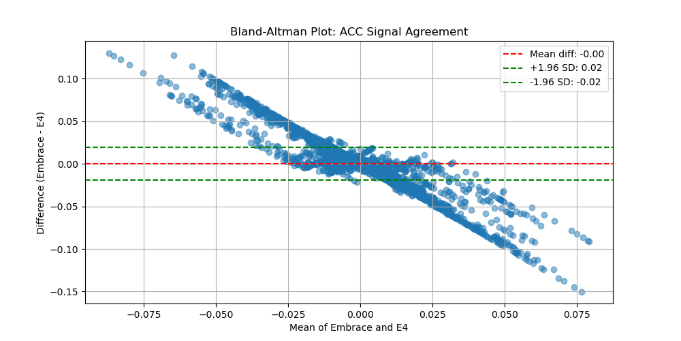
**

**F I G U R E E10 Bland-Altman Plots for the ACC-Y axis features of Zmax and Empatica E4.**

Bland–Altman analysis of the raw, sample-level Y-axis accelerometer signals revealed a negligible mean bias (bias ≈ −5.7 × 10⁻⁶), indicating the absence of a systematic offset between devices. The limits of agreement were relatively narrow (±0.019 in normalized units), suggesting small absolute differences on average. However, a pronounced proportional pattern was evident, with differences increasing as the magnitude of acceleration increased. This behavior is characteristic of accelerometer data and reflects the orientation-dependent and amplitude-sensitive nature of single-axis measurements.

**
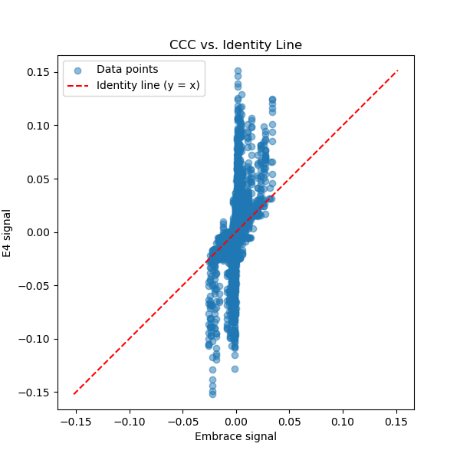

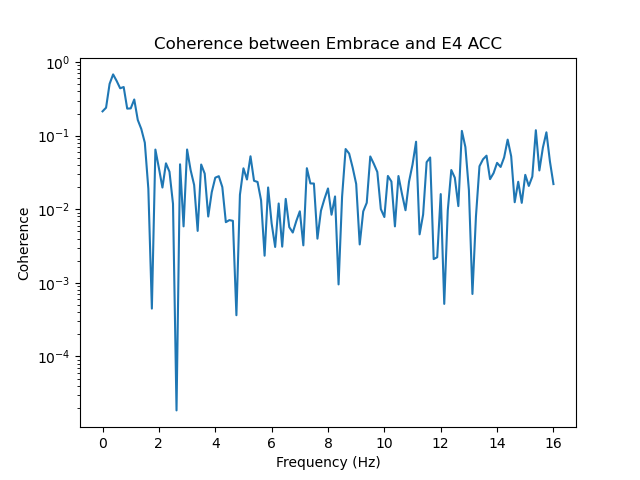
**

**F I G U R E E11 CCC Identity and Coherence Plots for the ACC-Y axis features of Zmax and Empatica E4.**

The concordance correlation coefficient for the aligned Y-axis signals was CCC = 0.56, consistent with the Pearson correlation. This indicates moderate concordance, reflecting partial agreement in both linear association and scale. Identity plots showed clustering of points around the line of equality but with substantial dispersion, highlighting residual differences in amplitude scaling and dynamic range between devices. These findings indicate that while the two devices capture similar movement trends along the Y-axis, the signals are not directly interchangeable in terms of absolute acceleration values. Magnitude-squared coherence analysis yielded a low mean coherence (≈ 0.16) across the analyzed frequency range. Elevated coherence was confined to very low frequencies, corresponding to slow postural adjustments and gross body movements, while coherence rapidly decreased at higher frequencies. This pattern reflects the broadband, non-stationary nature of accelerometer signals and the influence of device-specific filtering and sensor placement. As such, frequency-domain coherence provides limited insight into accelerometer synchronizability beyond confirming partial agreement in slow movement dynamics.

**
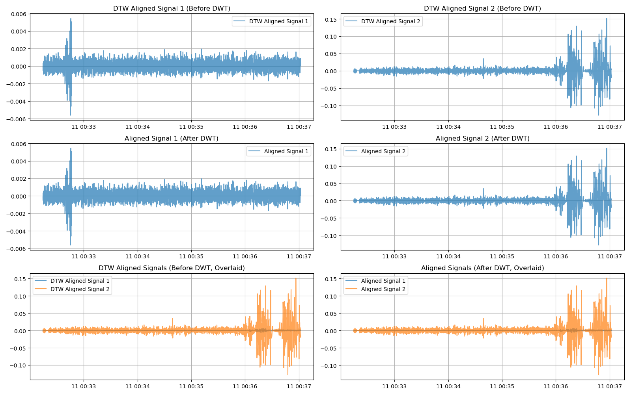

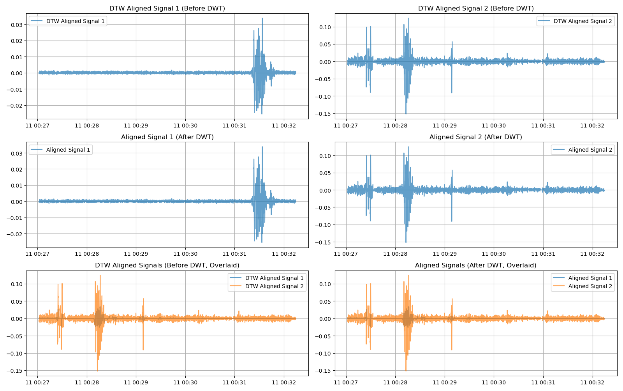
**

**F I G U R E E12 Examples to Final Aligned Signals for ACC Y-axis sensors of Zmax and Empatica E4.**

Taken together, these results indicate that synchronization substantially improves temporal correspondence of accelerometer Y-axis signals, enabling moderate agreement in movement dynamics across devices. However, waveform-level equivalence, phase synchronization, and frequency-domain coupling remain limited due to the orientation-dependent, non-periodic, and device-specific nature of single-axis accelerometer measurements. These findings support the use of aligned accelerometer data for event detection, activity segmentation, and feature-based analyses, rather than direct comparison of raw axis amplitudes. The accelerometer Y-axis demonstrated moderate post-alignment agreement, with improved temporal correspondence but limited waveform and frequency-domain equivalence, consistent with orientation-sensitive, non-periodic motion signals.

**E.3.3. Z- AXIS**

Phase synchronization analysis yielded a PLV of 0.45, which is notably higher than the corresponding values for the X- and Y-axes. While still below levels typically observed for periodic physiological signals, this moderate PLV suggests that certain vertical movement patterns—such as repetitive impacts or rhythmic motion—exhibit partial phase consistency across devices. This result reflects the more structured nature of vertical acceleration during common activities (e.g., walking), while still underscoring the limited applicability of phase-based metrics to non-oscillatory accelerometer data. Absolute error metrics indicated small pointwise discrepancies between aligned Z-axis signals (RMSE = 0.0066; MAE = 0.0024), representing the lowest error values among the three accelerometer axes. In contrast, the KL divergence was moderate (1.63), substantially lower than for the X- and Y-axes, indicating improved similarity in the overall distribution of vertical acceleration values across devices. This combination of low pointwise error and reduced distributional divergence suggests that the Z-axis provides the most stable basis for cross-device comparison among single-axis accelerometer signals.

**
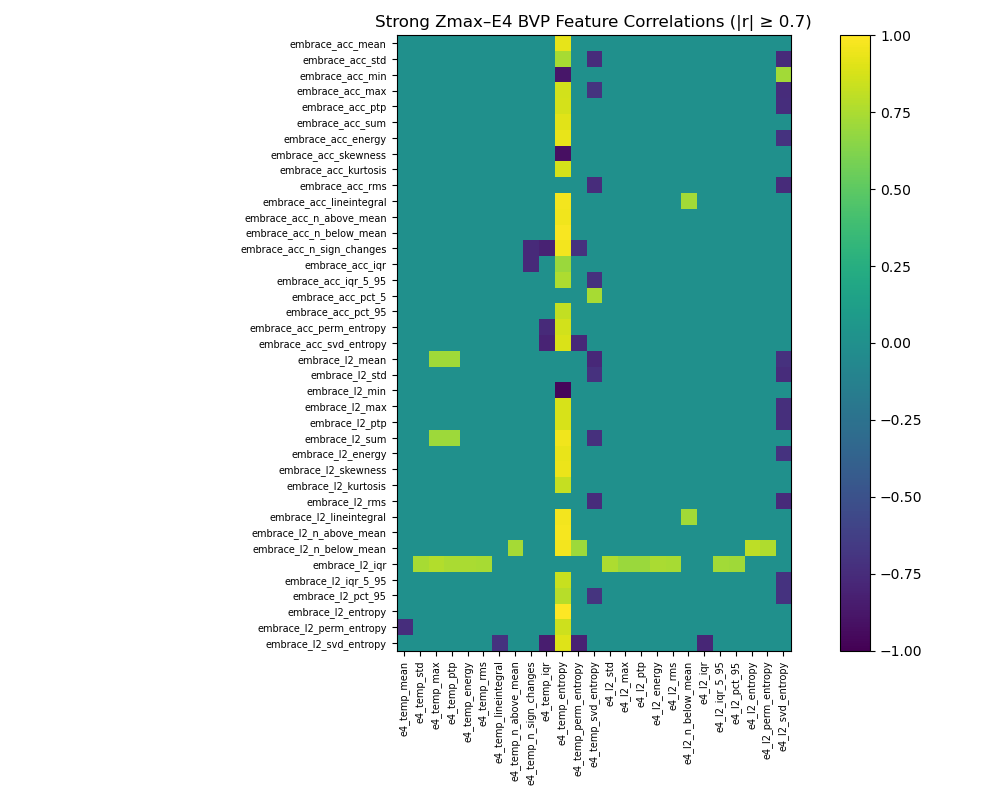
**

**F I G U R E E13 Correlation Matrix for the ACC-Z axis features of Zmax and Empatica E4.**

Prior to alignment, accelerometer Z-axis signals exhibited no meaningful correspondence between devices (Pearson r = −0.013; Spearman ρ = −0.012), confirming substantial temporal misalignment and asynchronous acquisition. After alignment, both linear and monotonic associations improved markedly, with a Pearson correlation of r = 0.61 and a Spearman correlation of ρ = 0.52. These values indicate moderate agreement and reflect successful recovery of temporal correspondence for vertical movement dynamics.

Compared to the X- and Y-axes, the Z-axis exhibited slightly stronger post-alignment correlations, consistent with its closer alignment to the gravitational axis and reduced sensitivity to device orientation differences during typical activities.

**
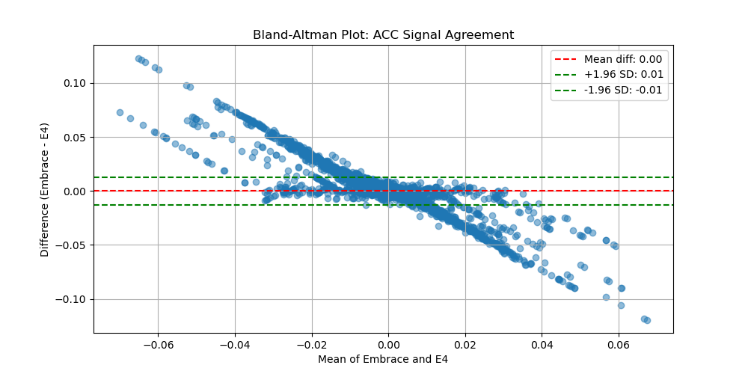
**

**F I G U R E E14 Bland-Altman Plots for the ACC-Z axis features of Zmax and Empatica E4.**

Bland–Altman analysis of the raw, sample-level Z-axis accelerometer signals demonstrated an essentially zero mean bias (bias ≈ 8.0 × 10⁻⁷), indicating the absence of systematic offset between devices. The limits of agreement were relatively narrow (±0.013 in normalized units), suggesting small average differences in vertical acceleration after alignment.

As observed for the other accelerometer axes, a clear proportional pattern was present, with larger discrepancies occurring during higher-magnitude acceleration events. This pattern reflects device-specific differences in sensitivity and dynamic range under increased movement intensity.

**
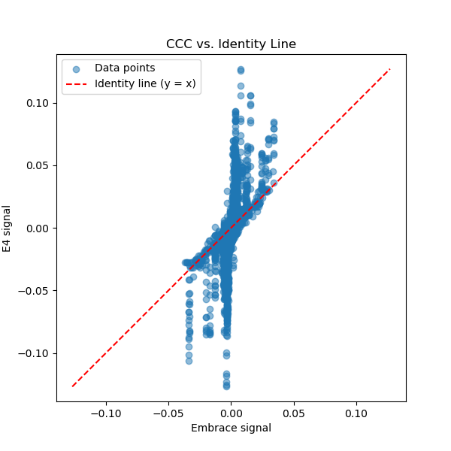

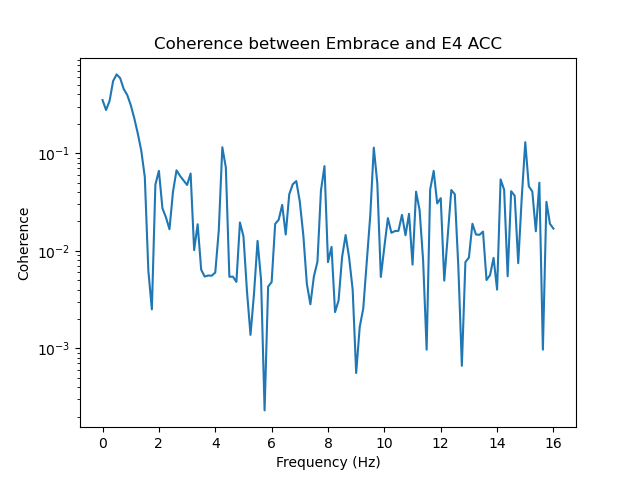
**

**F I G U R E E15 CCC Identity and Coherence Plots for the ACC-Z axis features of Zmax and Empatica E4.**

The concordance correlation coefficient for the aligned Z-axis signals was CCC = 0.61, consistent with the Pearson correlation and indicating moderate concordance. Identity plots revealed clustering of data points around the line of equality, with moderate dispersion reflecting residual amplitude scaling differences between devices. Compared to the X- and Y-axes, the Z-axis identity plot exhibited slightly tighter clustering, suggesting improved consistency in vertical acceleration measurements across devices. These findings indicate that, while absolute waveform interchangeability remains limited, relative changes in vertical acceleration are captured comparably following synchronization. Magnitude-squared coherence analysis produced a mean coherence of approximately 0.27, indicating modest frequency-domain agreement. Elevated coherence was primarily confined to low frequencies associated with slow vertical body movements and gravitational modulation. At higher frequencies, coherence decreased substantially and became noisy, reflecting transient, broadband motion components and device-specific filtering effects. Compared to the X- and Y-axes, the Z-axis exhibited slightly higher low-frequency coherence, consistent with its closer alignment to dominant movement directions and reduced sensitivity to rotational differences between devices.

**
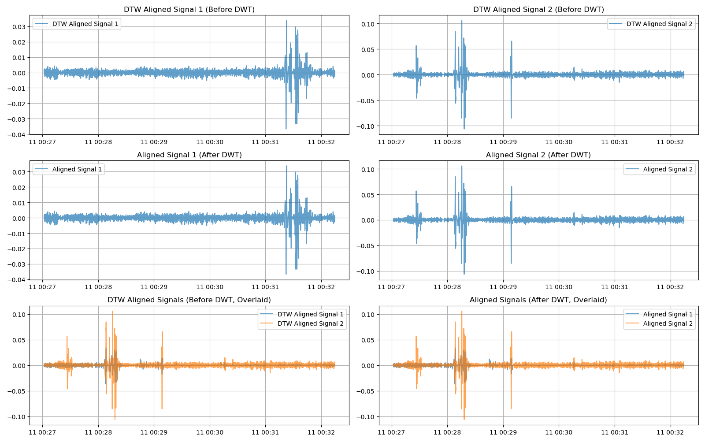

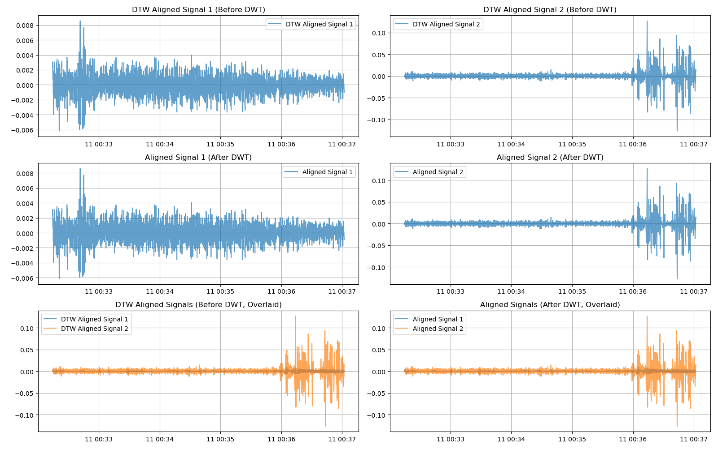
**

**F I G U R E E16 Examples to Final Aligned Signals for ACC Z-axis sensors of Zmax and Empatica E4.**

Overall, the accelerometer Z-axis demonstrated the strongest synchronizability among the three accelerometer axes. Alignment substantially improved temporal correspondence, yielding moderate correlations and concordance, minimal bias, and low absolute error. While waveform-level equivalence and broadband frequency coherence remained limited, as expected for accelerometer data, the Z-axis showed comparatively greater consistency due to its orientation relative to gravity and its role in capturing dominant vertical movement dynamics. These results support the use of synchronized Z-axis accelerometer data for feature-based analyses, activity segmentation, and movement intensity estimation across heterogeneous wearable devices. Among the accelerometer axes, the Z-axis exhibited the most consistent post-alignment agreement, with moderate correlation and concordance, minimal bias, and the lowest error and distributional divergence.

**E4. COMPARISON BETWEEN WITHIN VENDOR AND CROSS VENDOR RESULTS**

This section contrasts physiological signal agreement observed in within-vendor comparisons (Empatica E4 vs. EmbracePlus) with cross-vendor comparisons (Empatica E4 vs. Zmax), focusing specifically on Blood Volume Pulse (BVP) and Accelerometry (ACC), which were available in both analyses. While the same standardized synchronization and validation pipeline was applied in both cases, the experimental context differed substantially in terms of device manufacturer, sensor hardware, and body placement, allowing a principled comparison of how these factors influence cross-device interoperability. A key distinction between the two comparison scenarios is the degree of physical and technological similarity between devices:

**Within-vendor comparison (E4–EmbracePlus):**

- Both devices are manufactured by Empatica.
- Devices were worn simultaneously on the same wrist, with the EmbracePlus placed distally and the E4 proximally.
- Sensors share similar firmware design philosophy, preprocessing assumptions, and vendor-specific calibration procedures.

**Cross-vendor comparison (E4–Zmax):**

- Devices originate from different vendors, with independent hardware designs, sensor characteristics, and internal signal conditioning pipelines.
- Devices were worn on different body locations: the E4 on the wrist and the Zmax on the head.
- As a result, signals reflect not only device-specific differences but also distinct physiological measurement sites (peripheral vs. cranial).

These differences are fundamental when interpreting agreement metrics. In the within-vendor case, agreement reflects primarily sensor and placement effects, whereas in the cross-vendor case, agreement is constrained by anatomical, biomechanical, and physiological heterogeneity in addition to hardware differences.

While alignment substantially improved correlation relative to the pre-alignment values for the BVP signals, agreement remained well below within-vendor levels. This reduction is expected and physiologically meaningful: wrist-based BVP reflects peripheral vascular dynamics, whereas head-mounted sensors capture signals influenced by cranial vasculature, local pressure effects, and different motion artifacts. Thus, even when temporal patterns are partially synchronized, the underlying signals are not strictly equivalent.

Accelerometry exhibited the largest divergence between within-vendor and cross-vendor comparisons, underscoring the sensitivity of motion signals to placement, orientation, and device frame definitions. These findings indicate that when placement is shared, relative motion dynamics (timing and frequency structure) are preserved across devices, but absolute amplitudes depend on sensor gain, orientation, and local mechanics. The superior performance of the Z-axis likely reflects more stable device-frame dynamics normal to the skin surface. Even when synchronized in time, these signals reflect different movement generators (hand/arm gestures vs. postural or head movements), making strong agreement neither expected nor desirable.

Across both BVP and ACC, a consistent pattern emerges:

**Within-vendor, same-location comparisons** yield:

- High concordance
- Narrow limits of agreement
- Strong spectral alignment
- Practical interchangeability for many analyses

**Cross-vendor, different-location comparisons** yield:

- Moderate post-alignment correlation
- Reduced concordance
- Higher divergence in amplitude distributions
- Signals that are synchronized in time but not physiologically identical

Crucially, the reduced agreement in the cross-vendor case should not be interpreted as a failure of the synchronization pipeline. Rather, it reflects a realistic upper bound imposed by anatomical placement and vendor-specific sensor design. The pipeline successfully recovers shared temporal structure where it exists, while the remaining discrepancies correctly reflect true physiological and biomechanical differences. Within-vendor devices worn at the same anatomical site (E4–EmbracePlus) can often be treated as interchangeable after standardized preprocessing. In contrast, cross-vendor comparisons across different body locations (E4–Zmax) are best suited for trend-based, relative, or multimodal analyses, rather than absolute signal equivalence.

An important practical outcome of this comparison is that the proposed synchronization and validation pipeline does not merely quantify agreement, but also guides modality selection in cross-vendor deployments. Specifically, results from the E4–Zmax analysis indicate that BVP is a substantially more reliable signal than accelerometry for cross-vendor synchronization when devices are worn at different body locations. Across all evaluated metrics, BVP consistently demonstrated stronger post-alignment agreement than accelerometer signals. For E4–Zmax, BVP achieved moderate correlations and concordance (Pearson r ≈ 0.61; CCC ≈ 0.61), low absolute error (RMSE ≈ 0.0066; MAE ≈ 0.0024), moderate phase consistency (PLV ≈ 0.45), and lower distributional divergence (KL ≈ 1.63) relative to accelerometer axes. In contrast, accelerometry, particularly in the X and Y axes, exhibited lower concordance, higher KL divergence, fragmented frequency-domain coherence, and strong dependence on sensor placement and orientation.
