# Supplementary material for: Standardized synchronization and validation pipeline for physiological biomarkers across multiple devices
Source: Behav Res Methods. 2026 Jul 16;58(8):239. doi: 10.3758/s13428-026-03097-8 (PMC13375695; doi:10.3758/s13428-026-03097-8)
Supplement: Supplementary file 2 — Supplementary file2 (DOCX 4942 KB) [file 13428_2026_3097_MOESM2_ESM.docx]

**Supplementary Figures**

**(Can be provided as a separate files (.jpg/.png) if requested)**

**FLOWCHART OF THE PIPELINE**

**
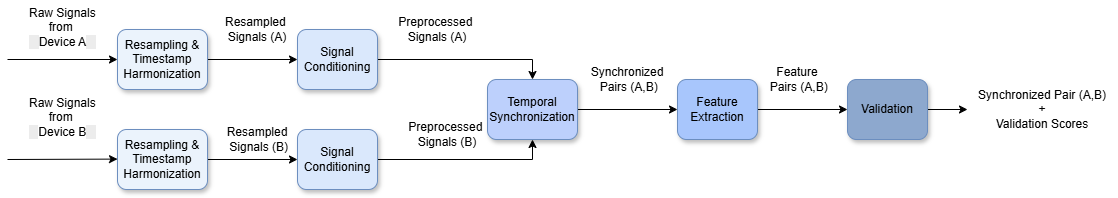
**

**CROSS-VENDOR ZMAX-EMPATICA ACC-X**

**
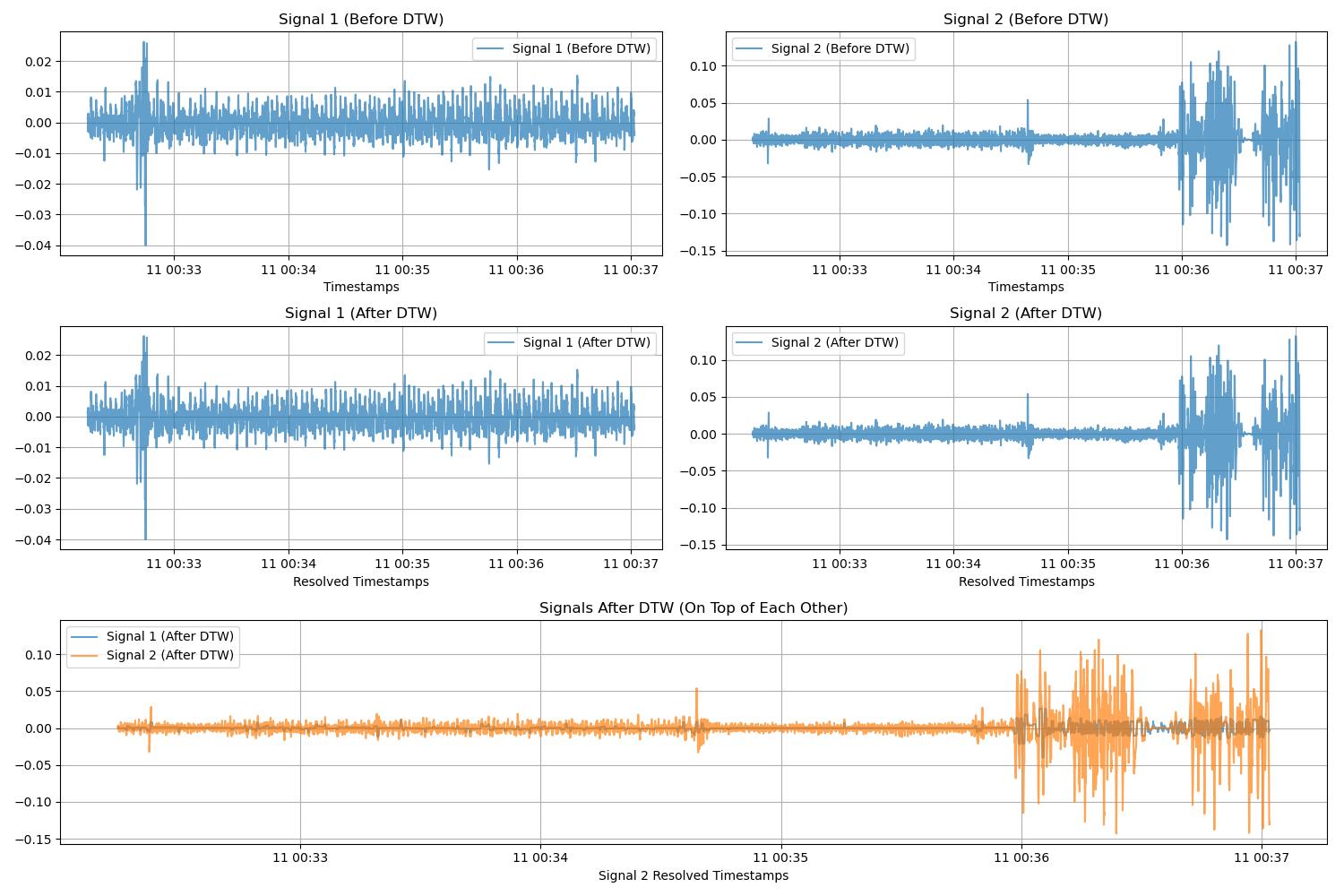

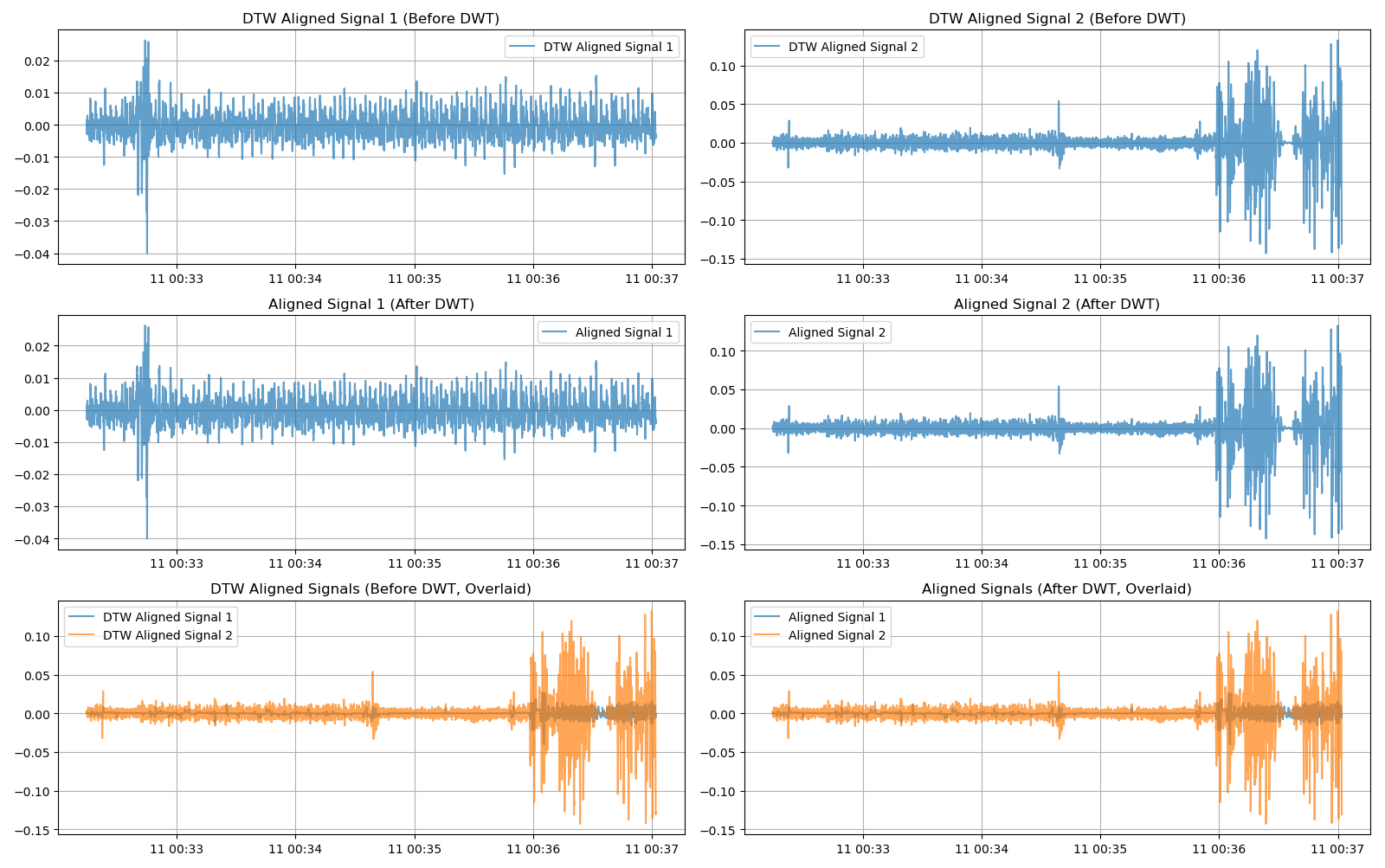

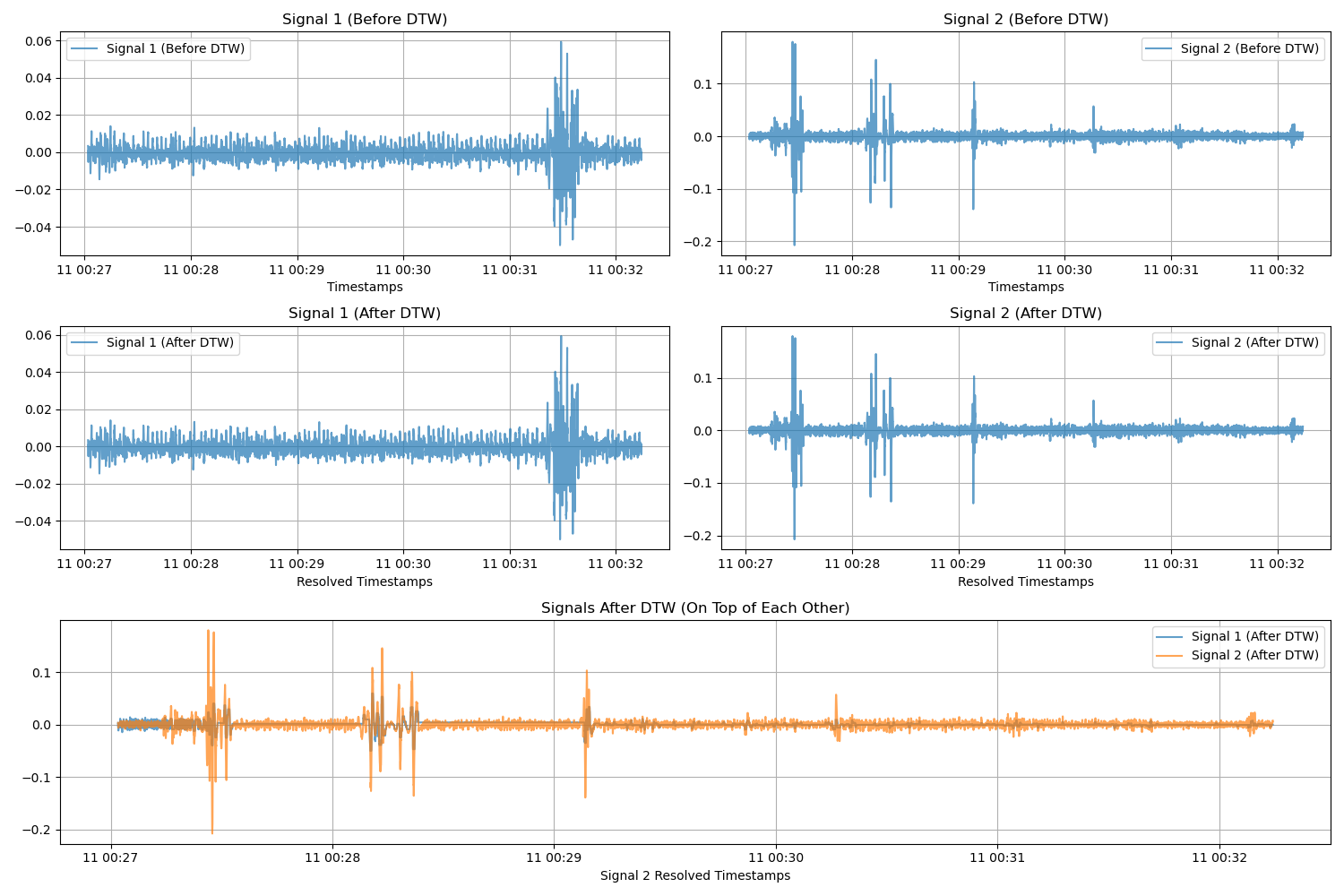

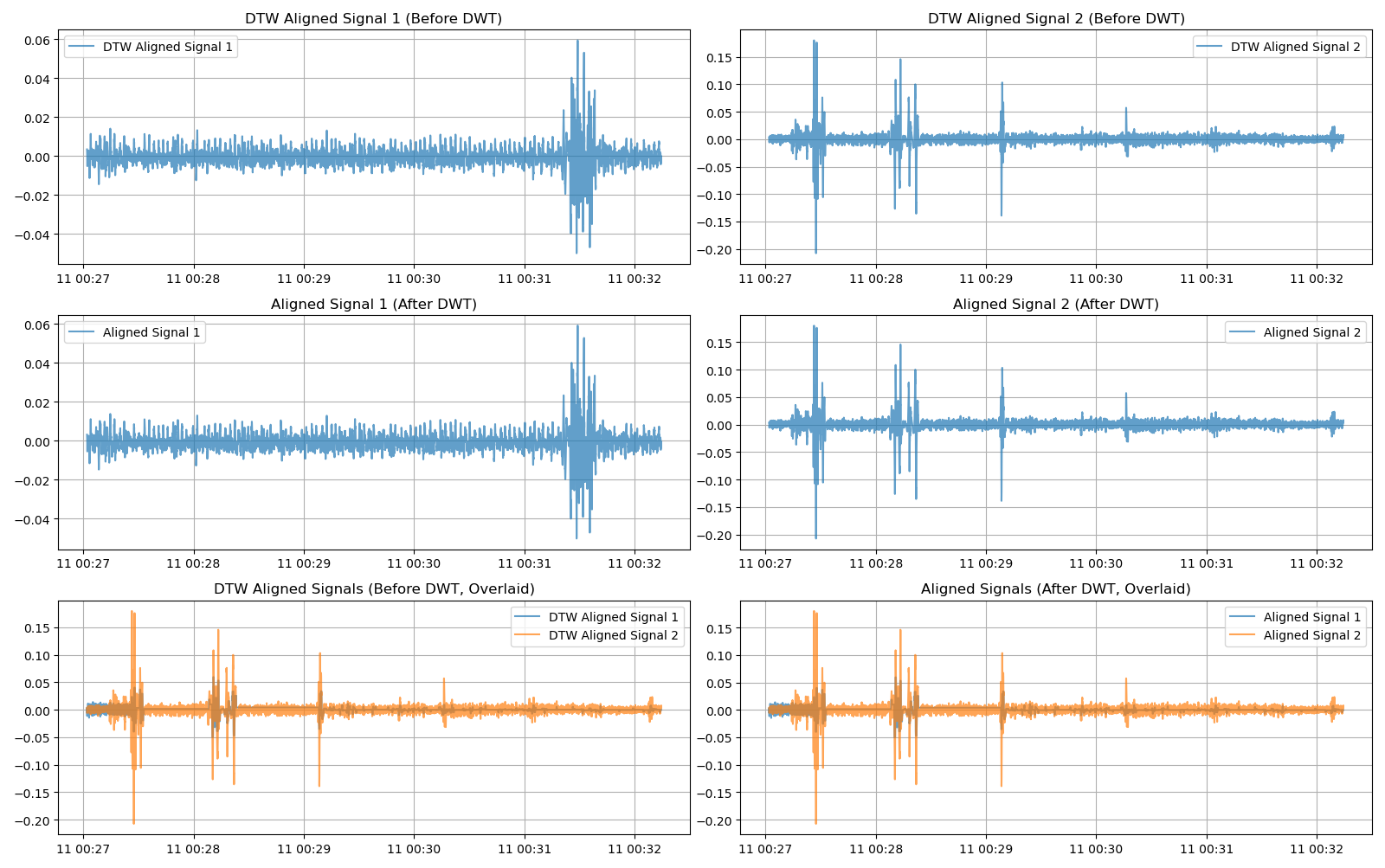

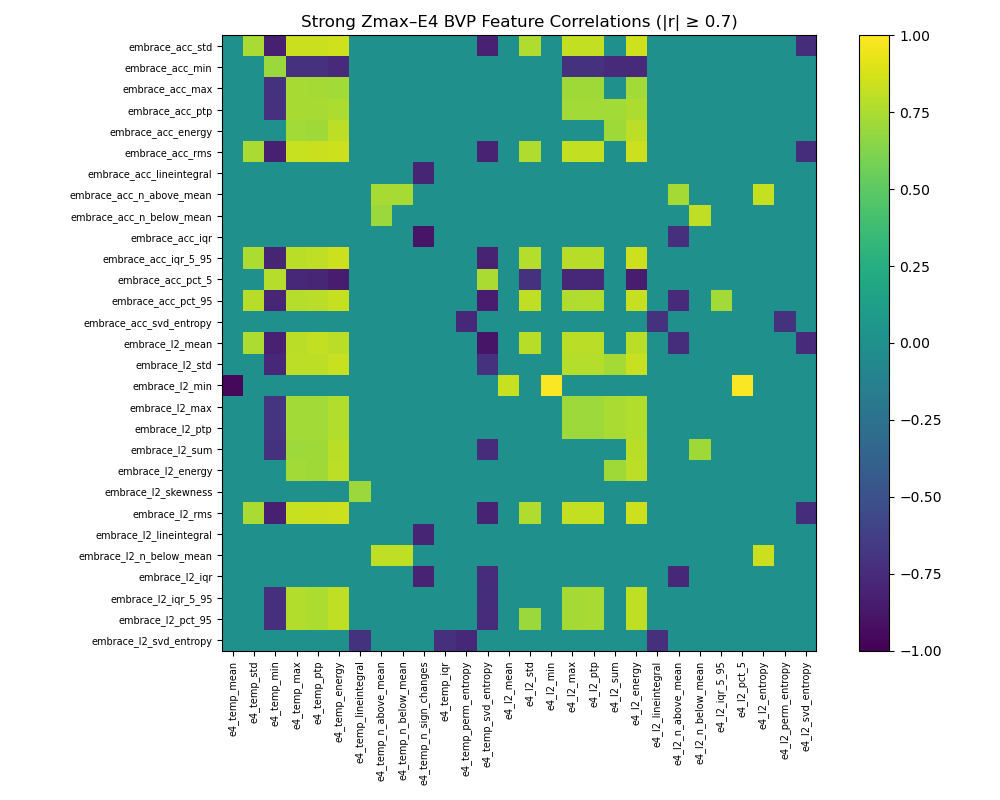

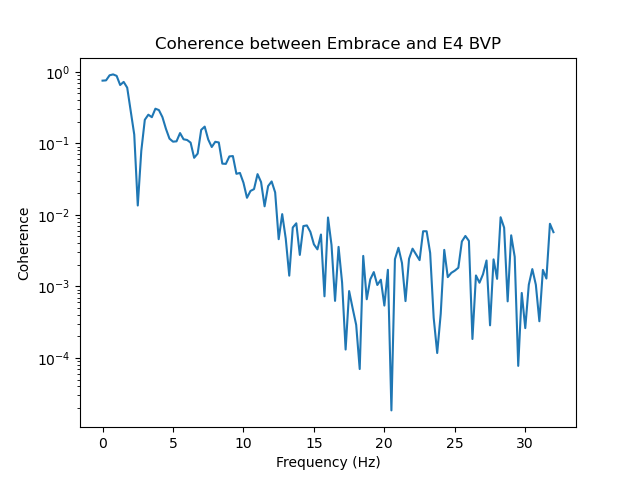

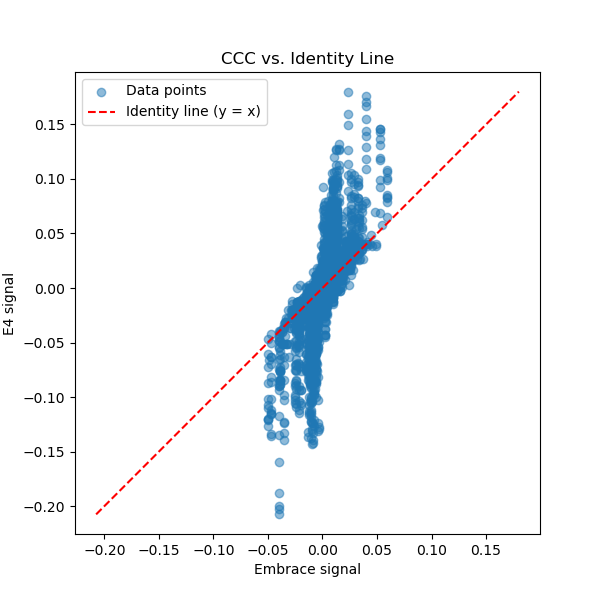

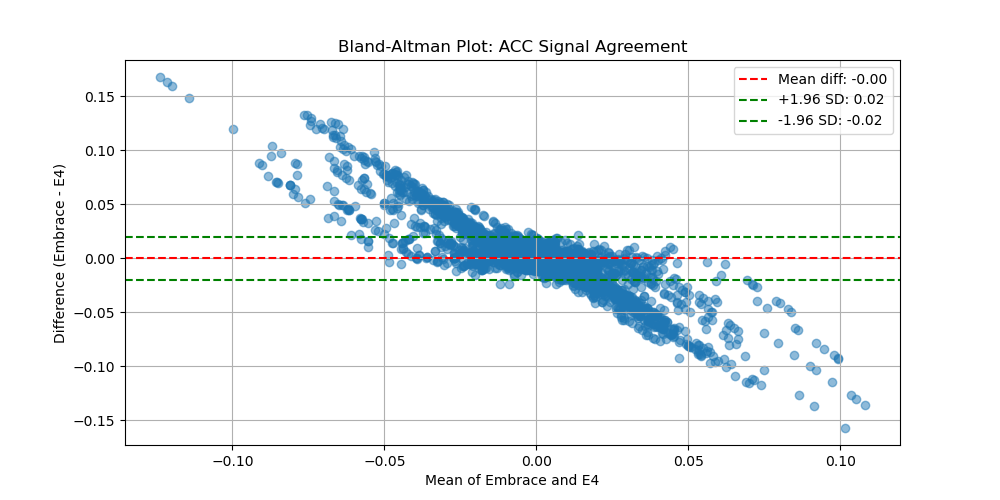
**

**CROSS-VENDOR ZMAX-EMPATICA ACC – Y**

**
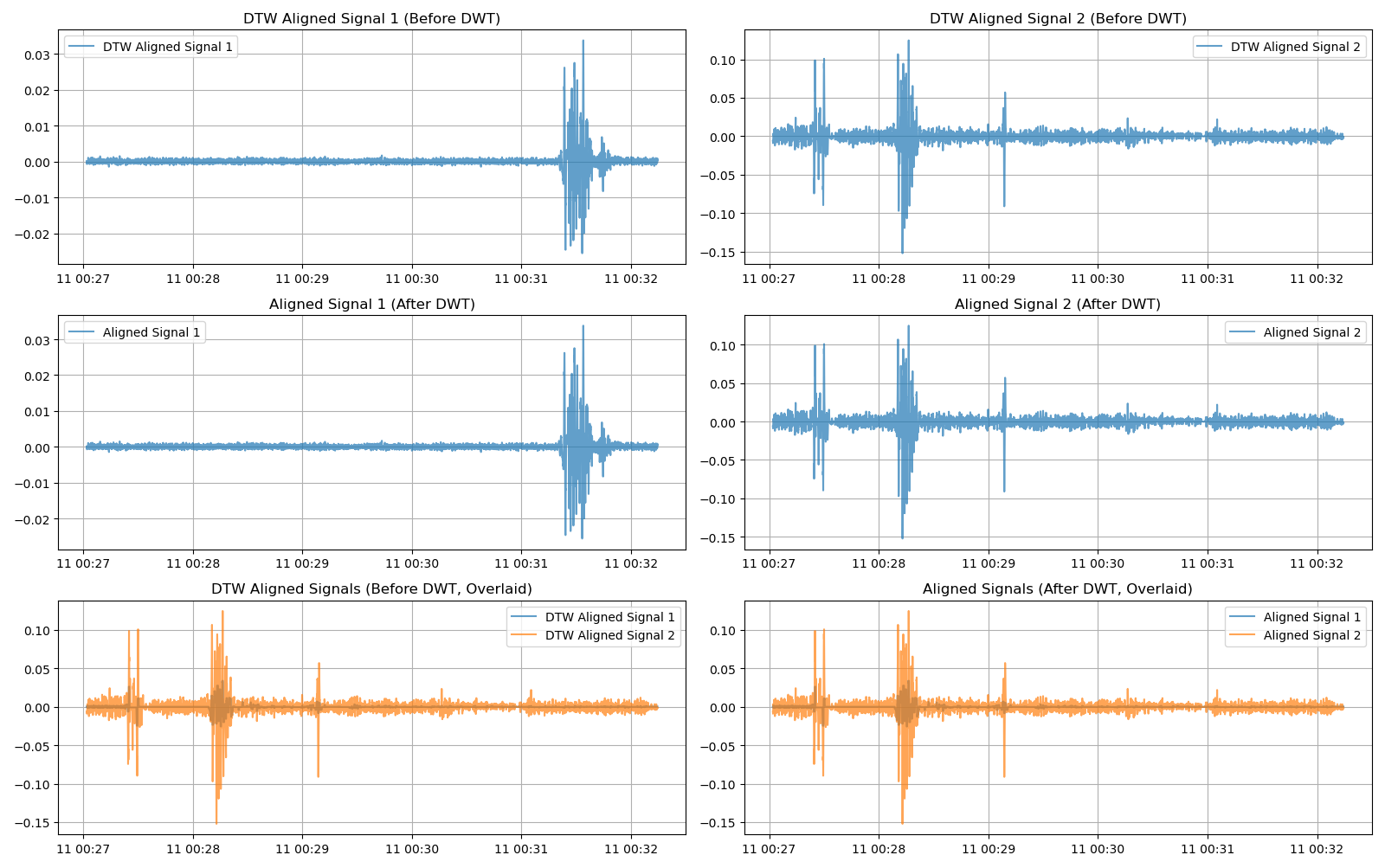

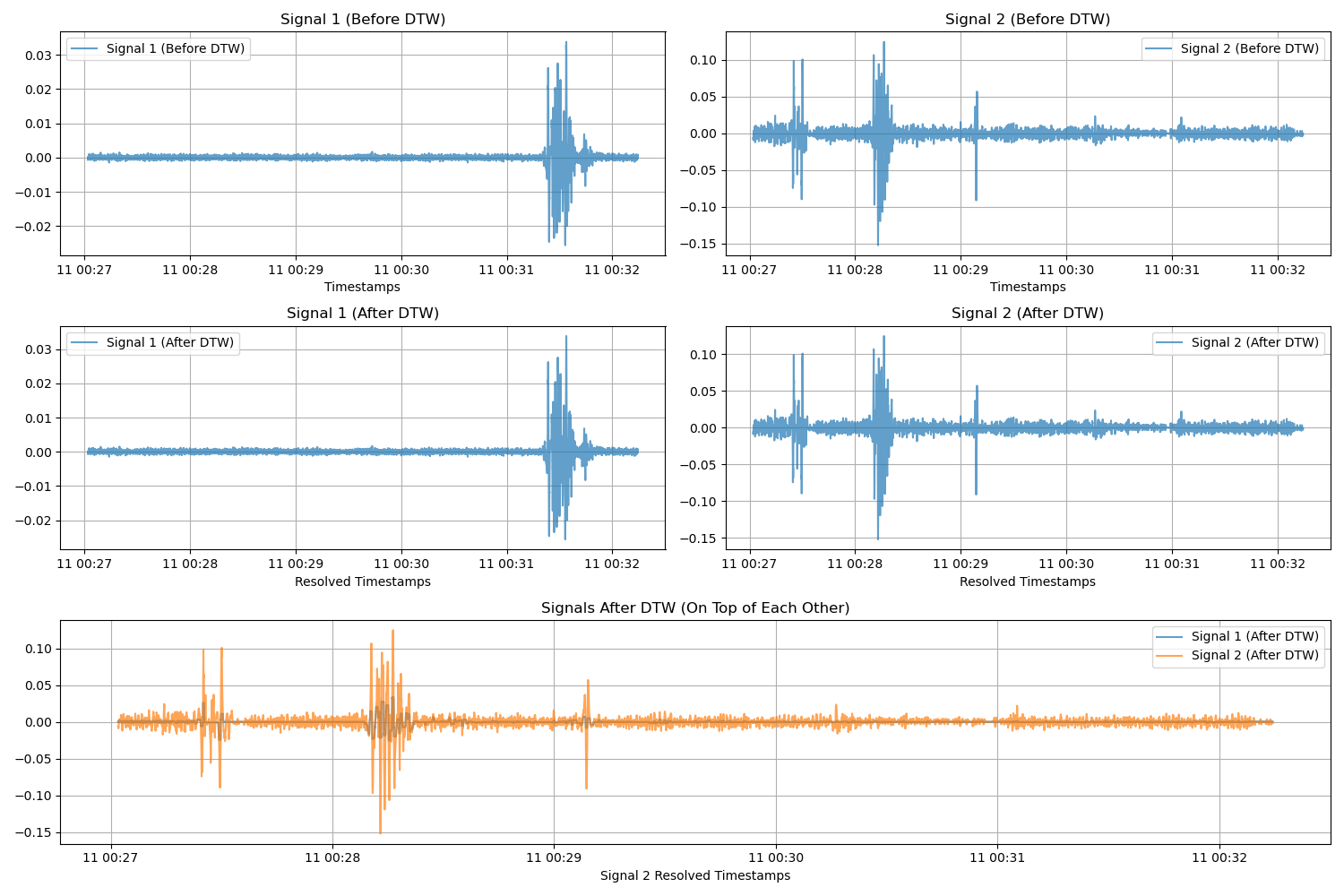

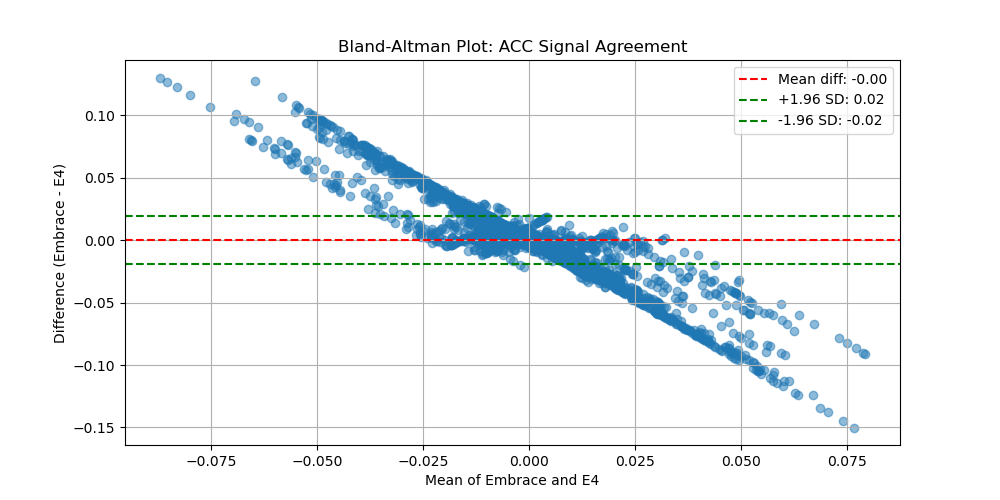

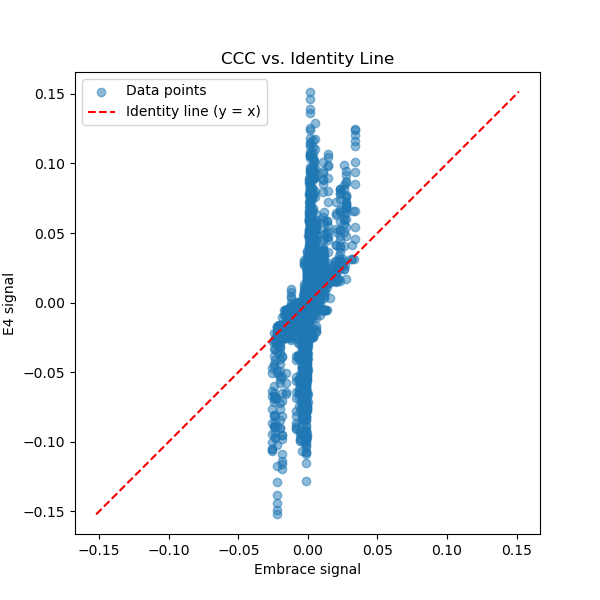

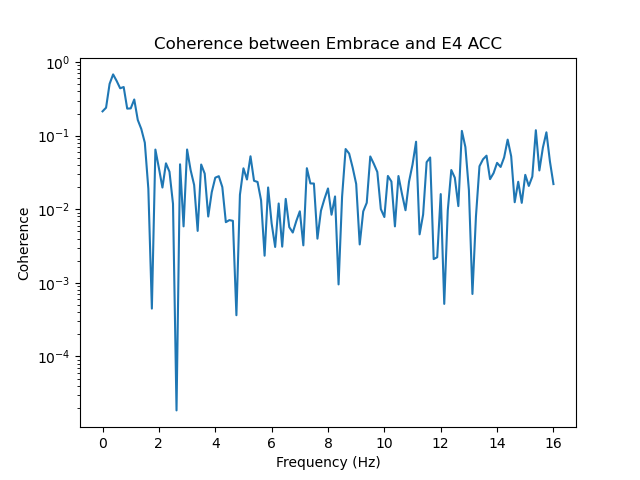

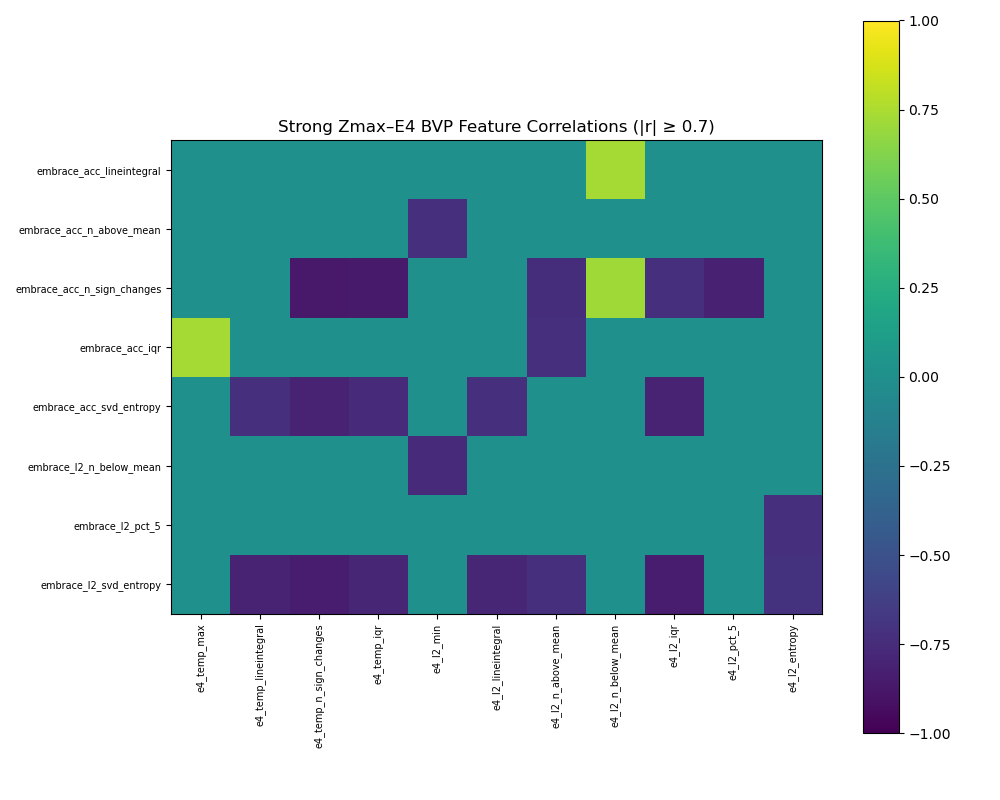

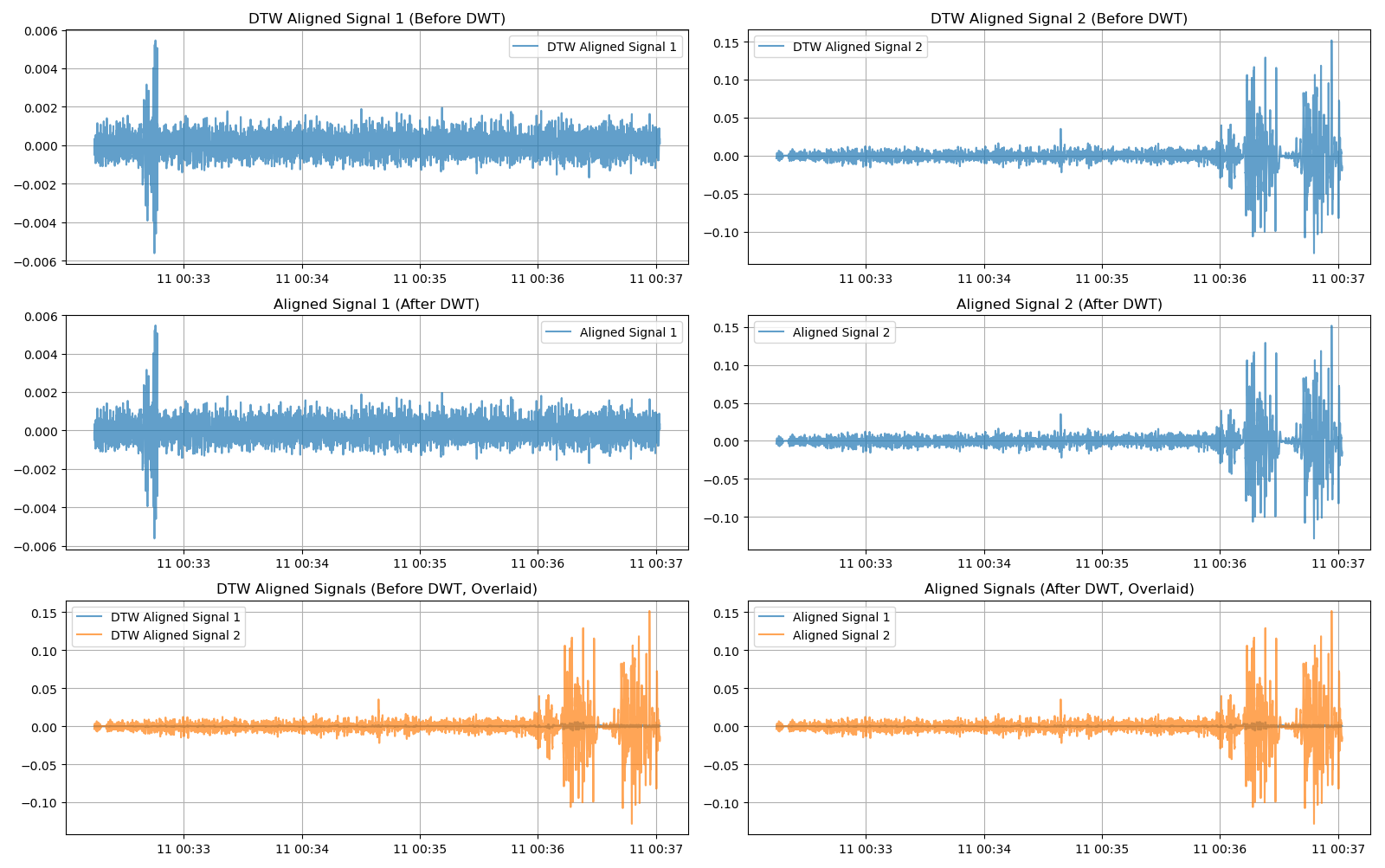
**

**CROSS-VENDOR ZMAX-EMPATICA ACC- Z**

**
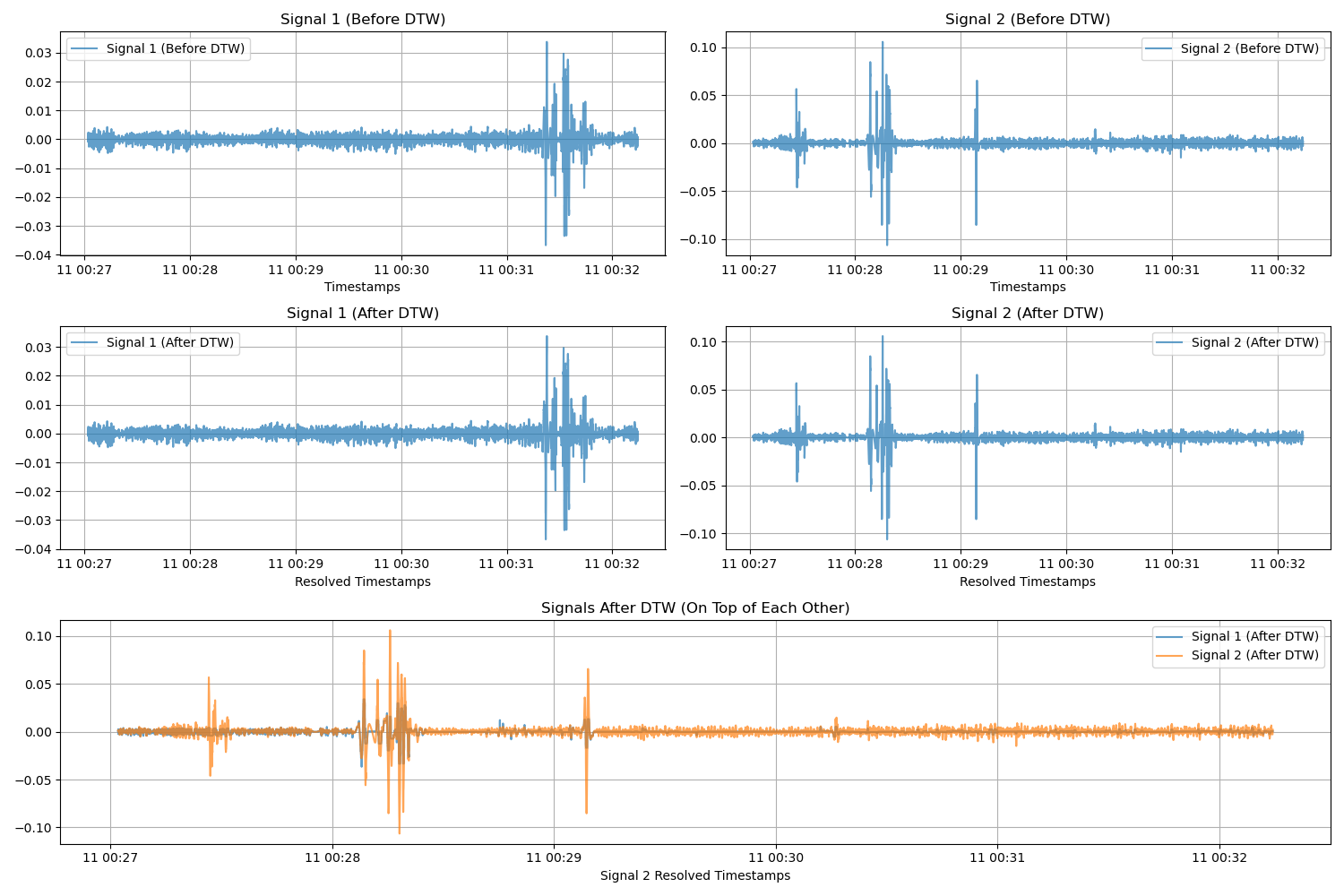

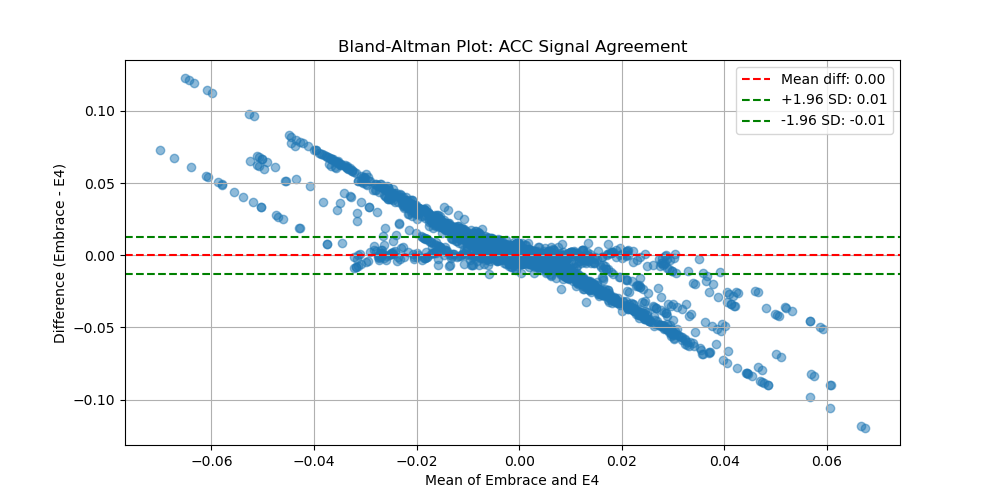

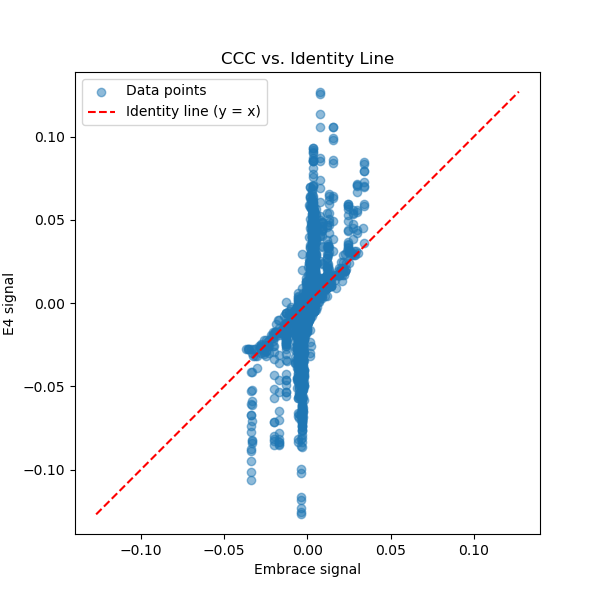

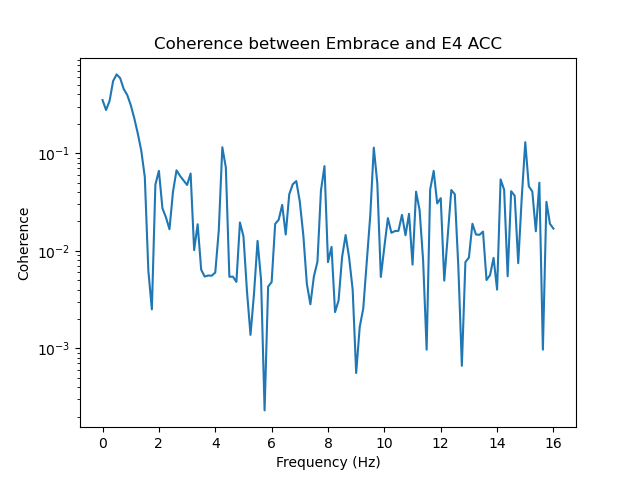

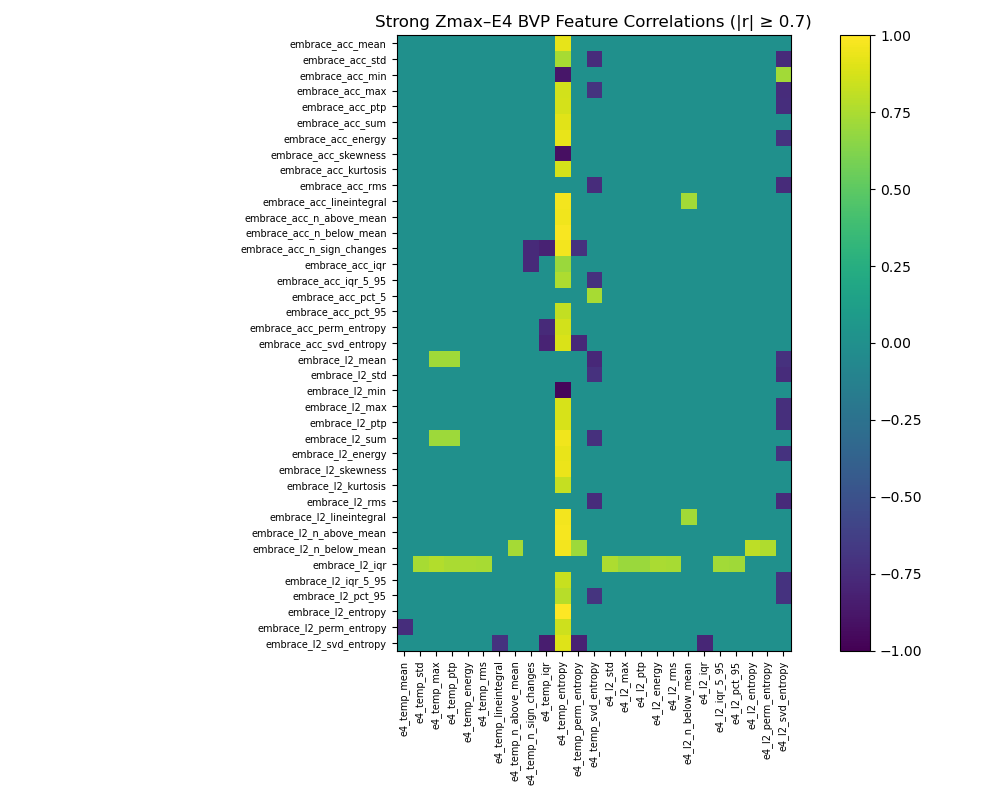

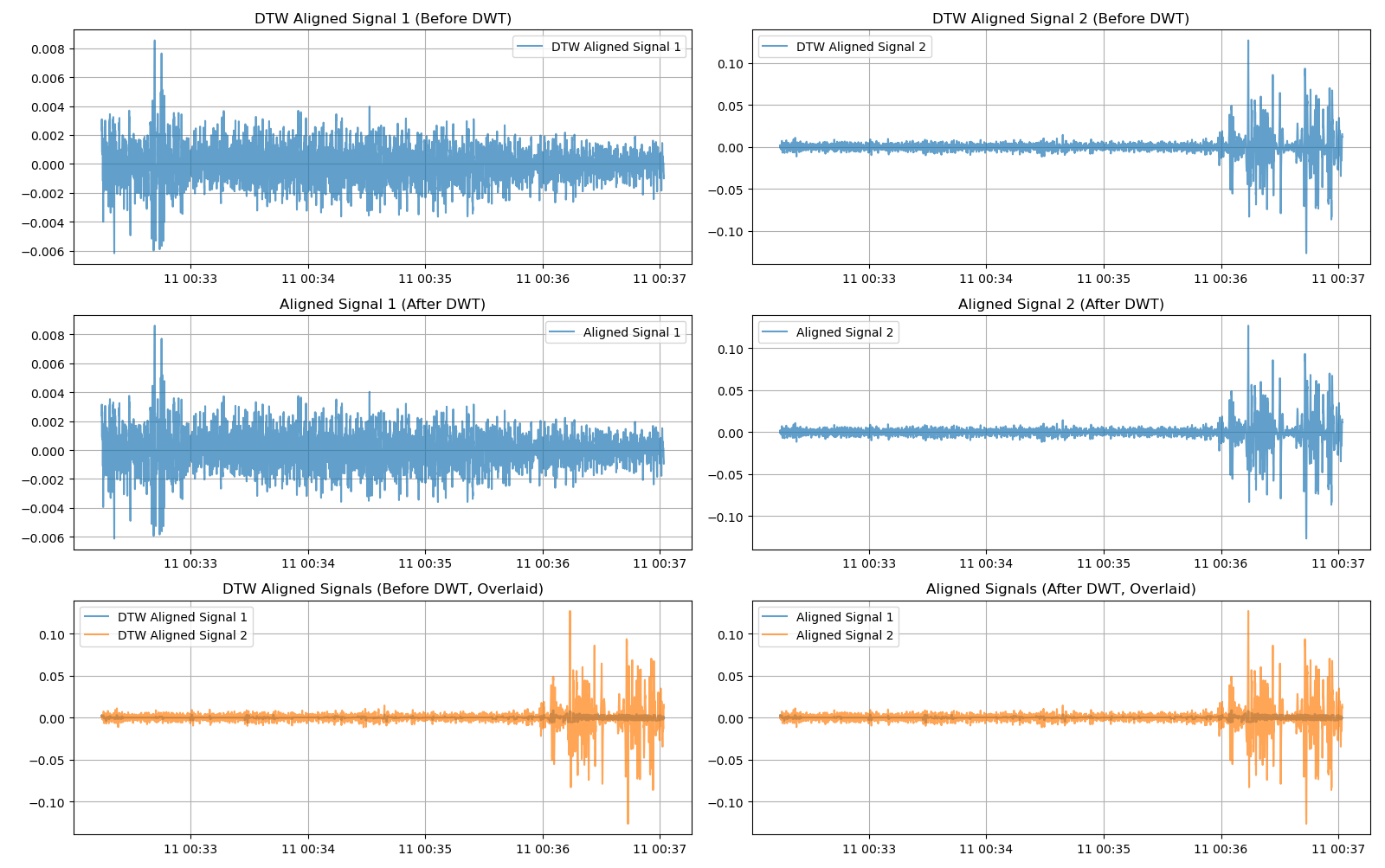

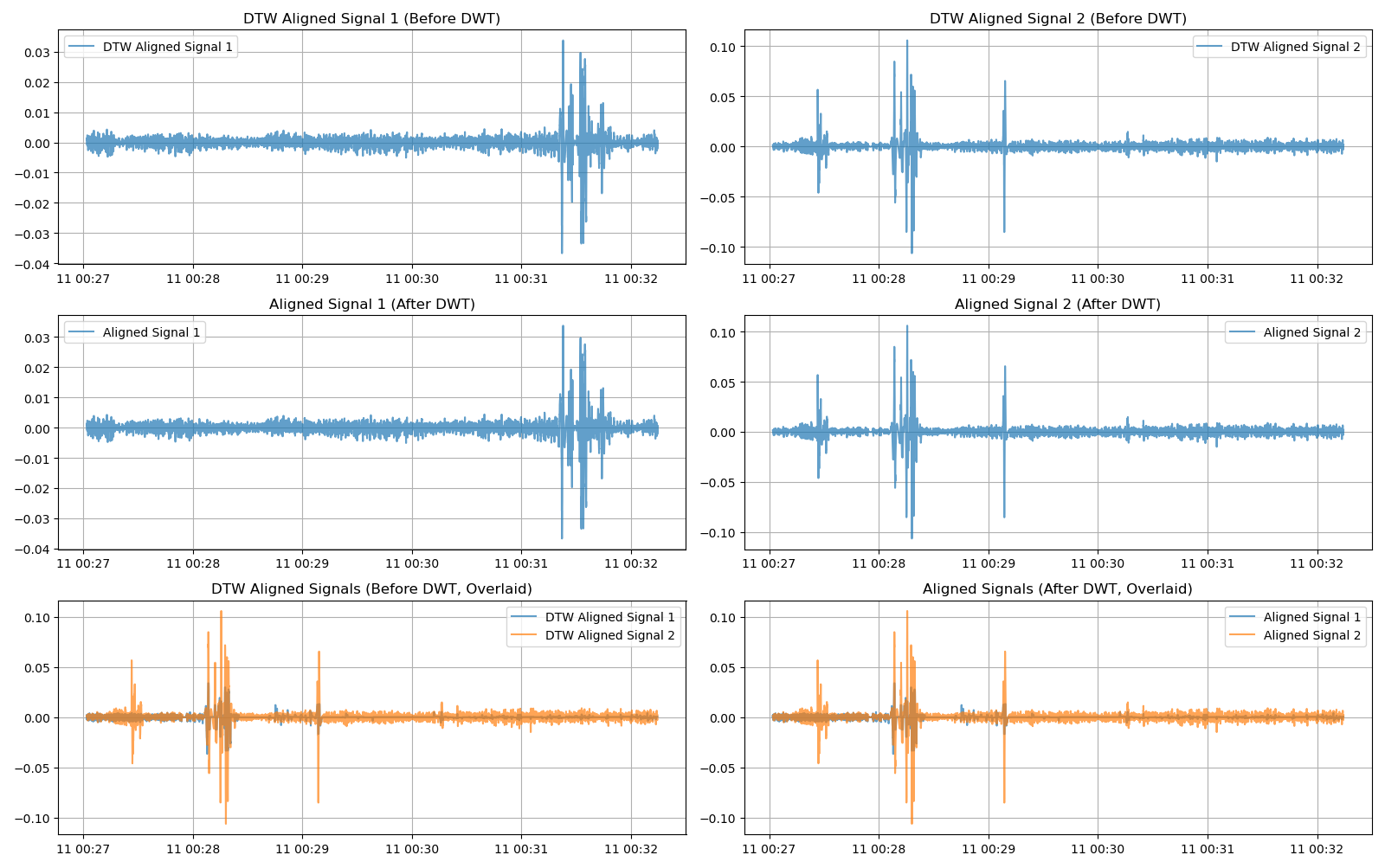
**

**CROSS-VENDOR ZMAX-EMPATICA BVP**

**
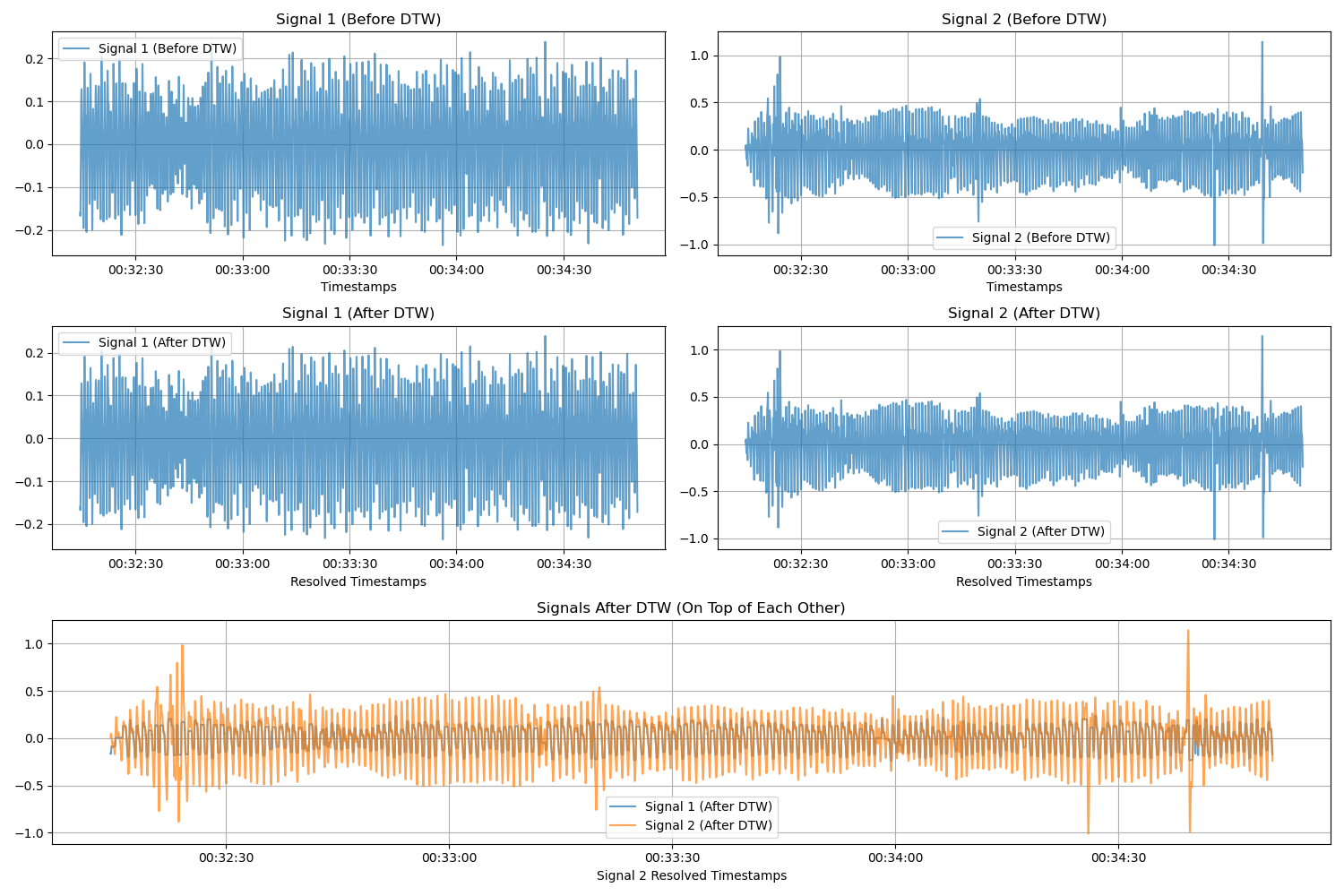

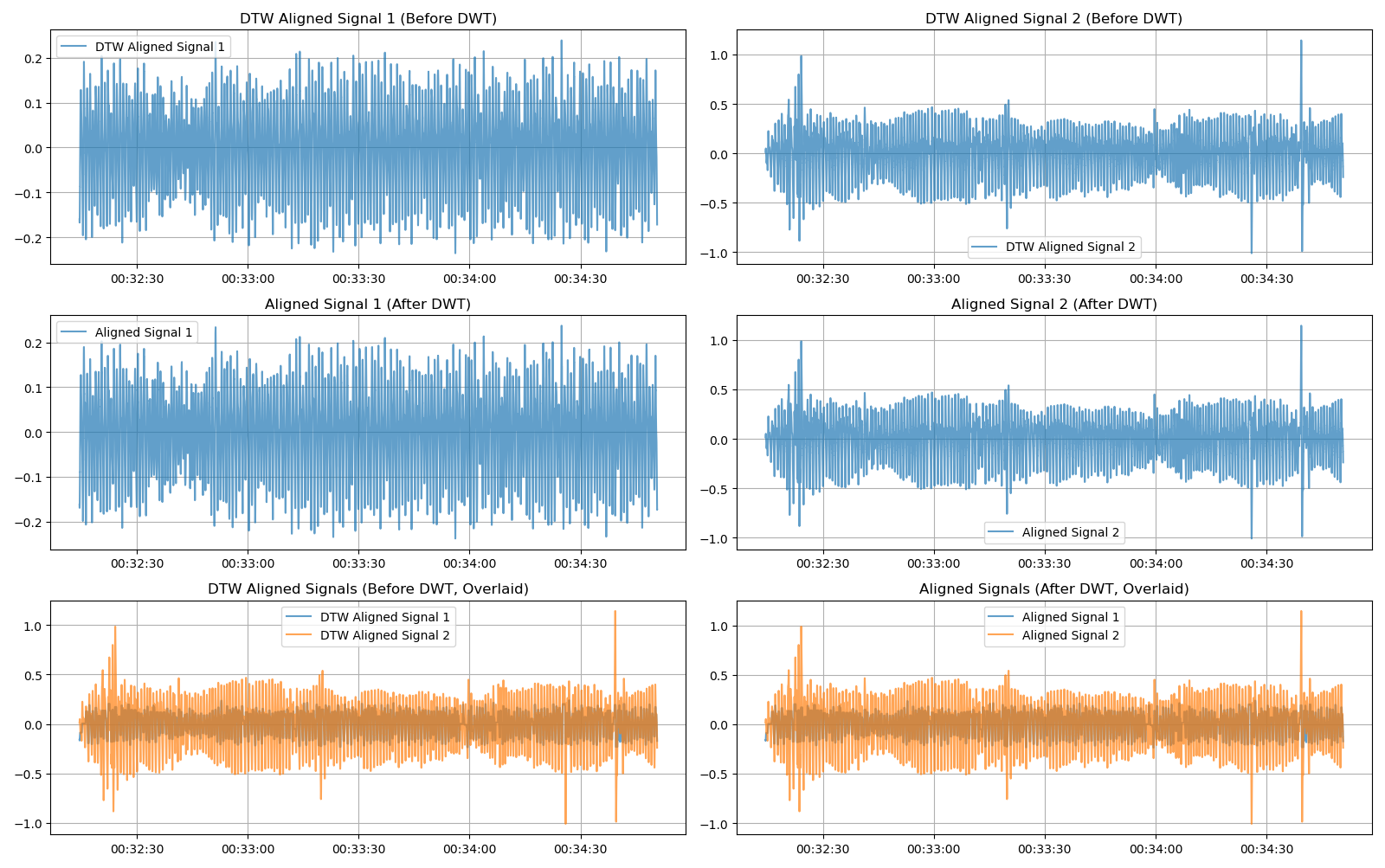

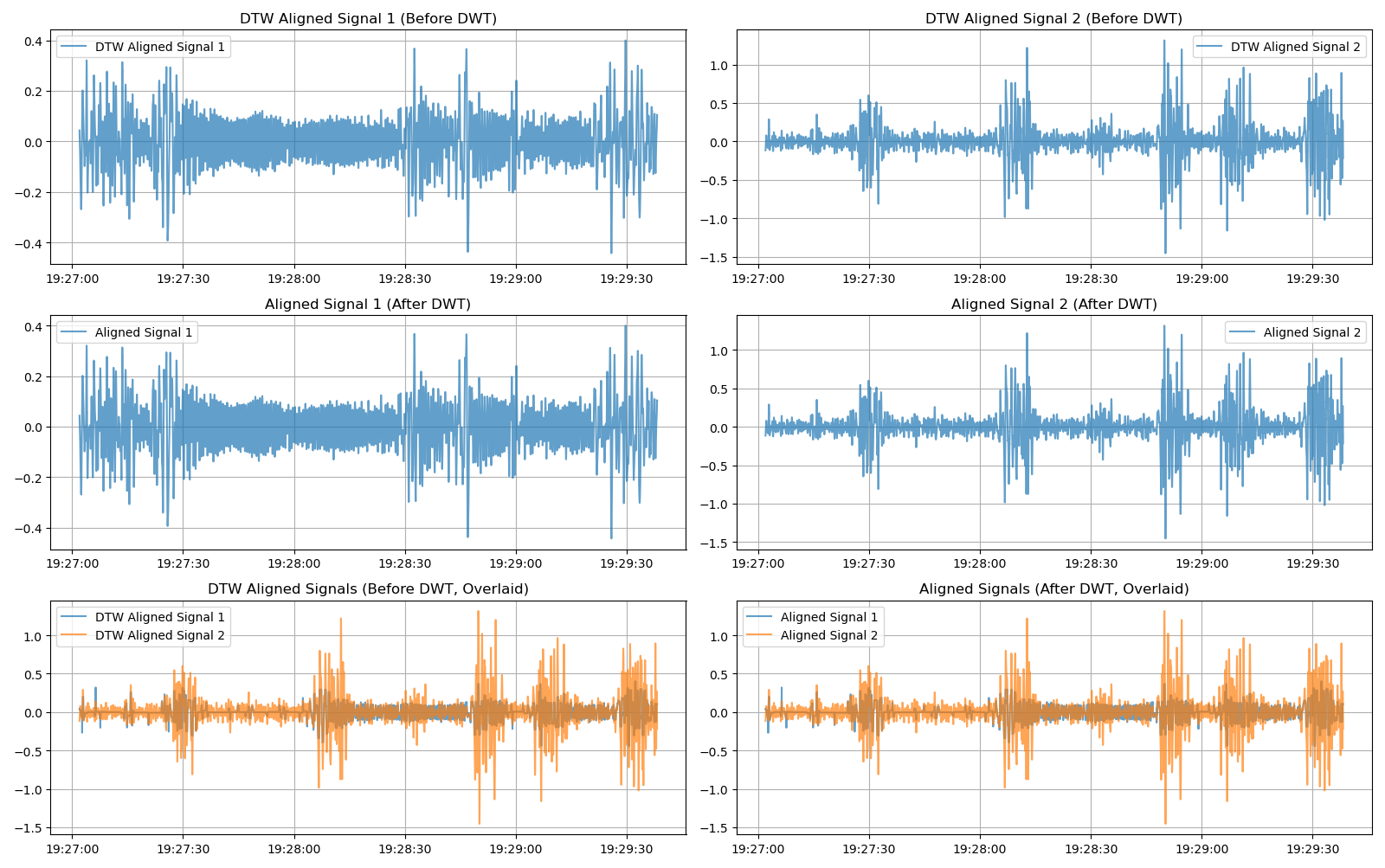

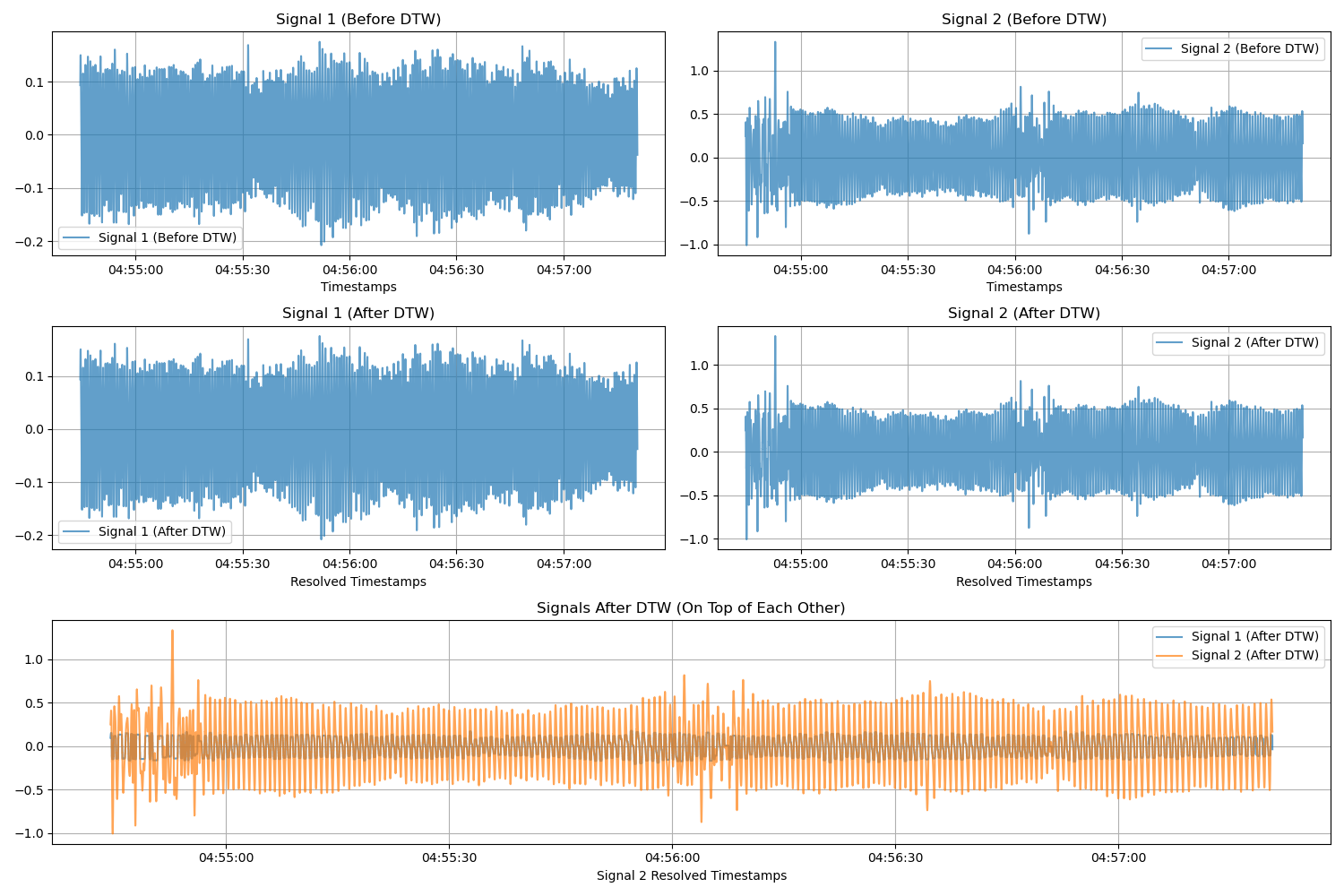

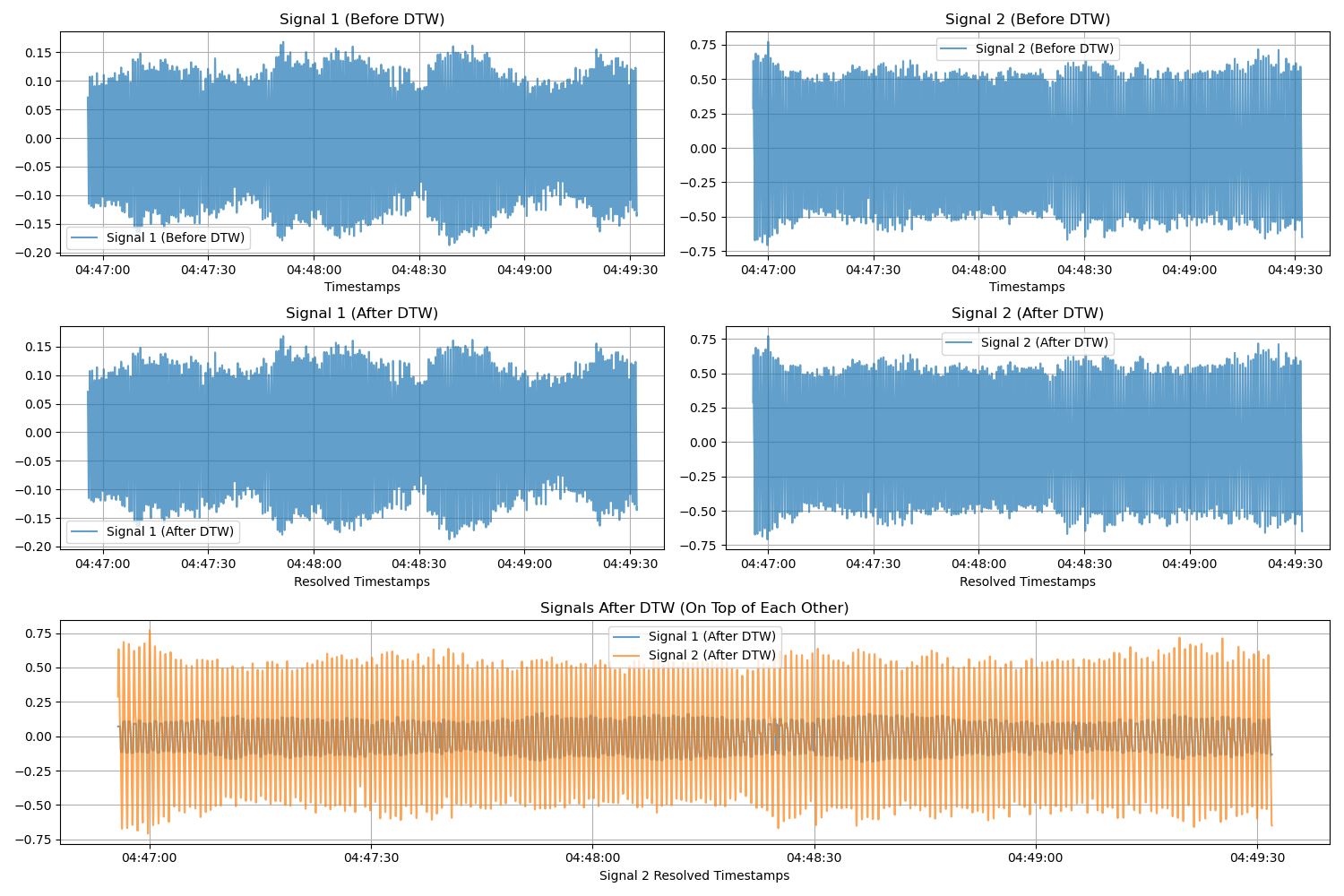

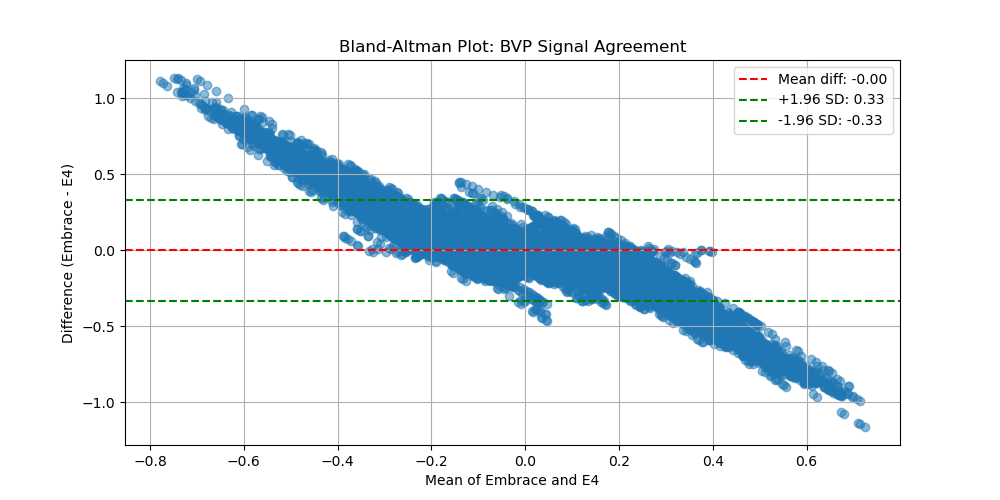

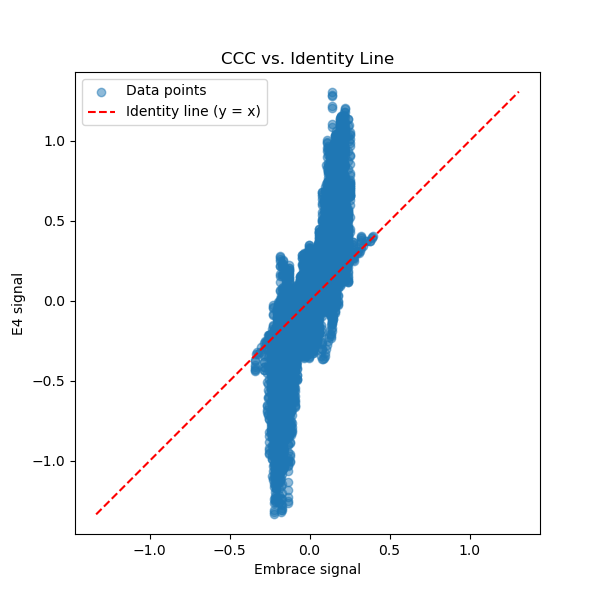

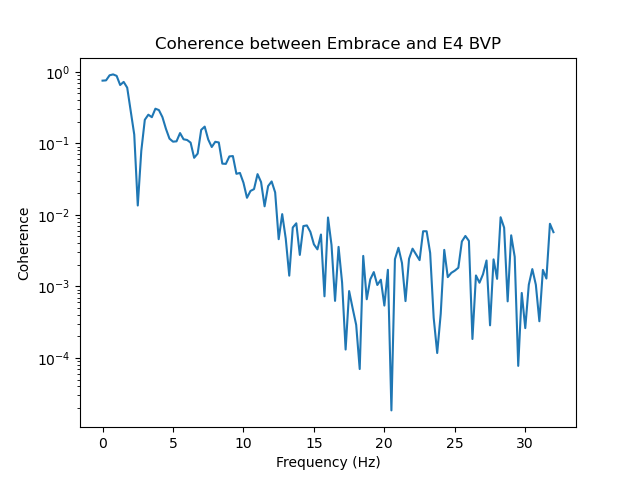

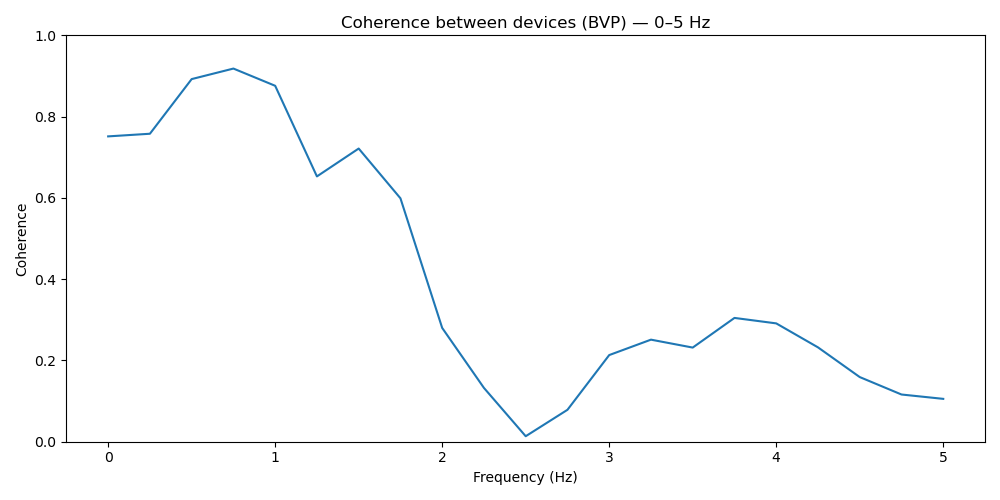

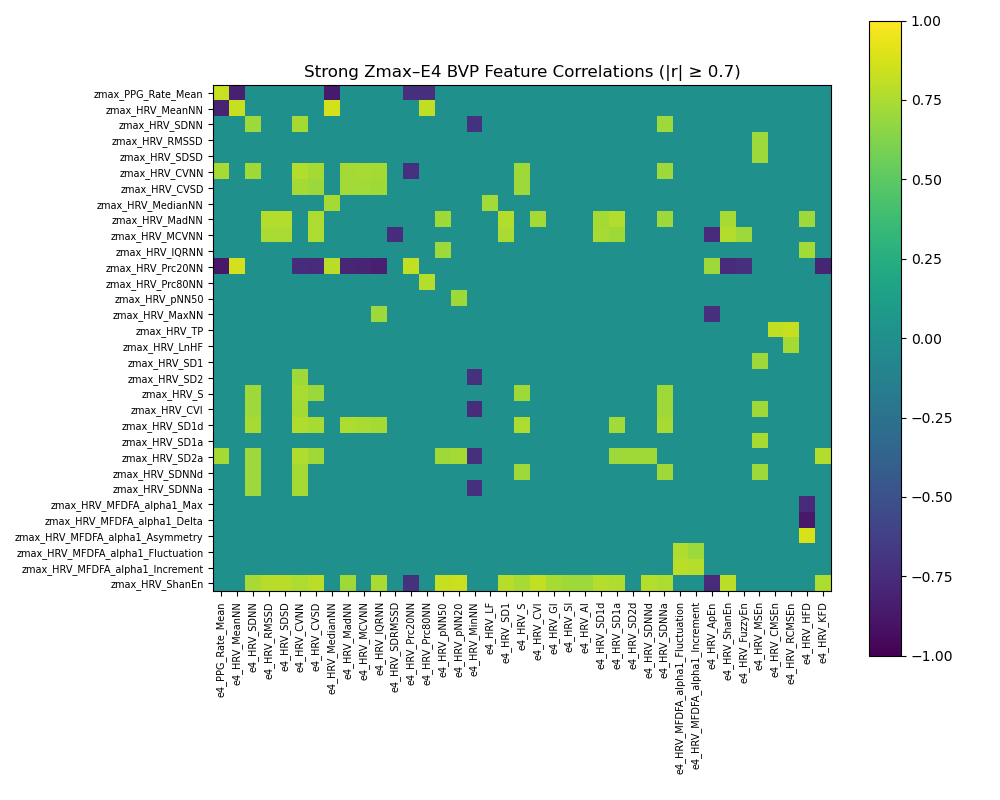

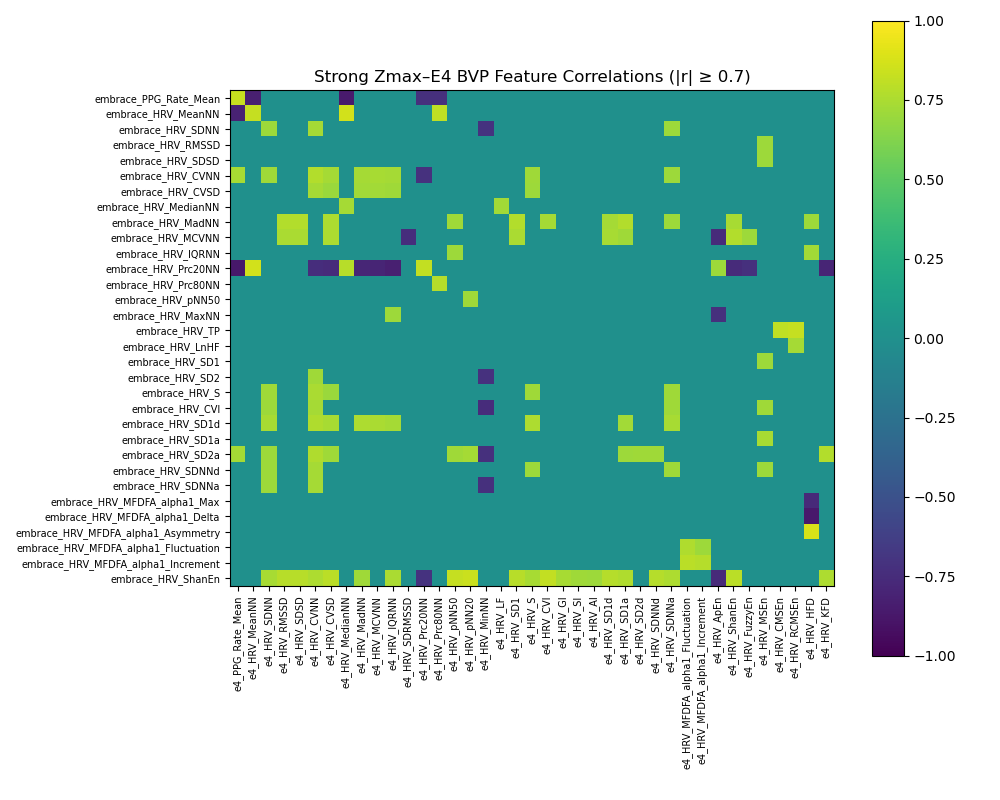
**
